# Fumarate-induced succination of A-kinase anchor protein 12 exacerbates renal inflammation and fibrosis

**DOI:** 10.1172/JCI200755

**Published:** 2026-06-30

**Authors:** Shuai Sun, Xu-yang Yan, Yu-hang Dong, Jian-min You, Zhen-yu Guo, Dong-xue Lv, Shuai-shuai Xie, Rui Hou, Xiang-yu Li, Ju-tao Yu, Xiao-yu Shen, Jie Wei, Zhen-yu Song, Zi-qi Chen, Yun-long Zhu, Xing-xin Xu, Juan Jin, Jia-gen Wen, Hao Wang, Xiao-ming Meng, Wei Wang

**Affiliations:** 1 Department of Urology, The Fifth Affiliated Hospital of Anhui Medical University, Fuyang, China.; 2 Department of Urology, The First Affiliated Hospital of Anhui Medical University, Hefei, China.; 3 Anhui Province Key Laboratory of Urological and Andrological Diseases Research and Medical Transformation, Institute of Urology;; 4 Inflammation and Immune Mediated Diseases Laboratory of Anhui Province, the Key Laboratory of Anti-inflammatory of Immune Medicines, Ministry of Education, Anhui Institute of Innovative Drugs, School of Pharmacy; and; 5 Department of Toxicology, School of Public Health, Anhui Medical University, Hefei, China.; 6 Department of Nephrology, The First Affiliated Hospital of Anhui Medical University, Hefei, China.; 7 Department of Urology, The Affiliated Jiangning Hospital with Nanjing Medical University, Nanjing, Jiangsu, China.

**Keywords:** Inflammation, Metabolism, Nephrology, Chronic kidney disease, Fibrosis

## Abstract

The inflammatory response resulting from the abnormal accumulation of metabolites has been implicated in the pathogenesis of organ fibrosis; however, its role and underlying mechanisms in renal fibrosis remain unclear. In this study, we observed a negative correlation between fumarate hydratase (FH) expression and the degree of renal fibrosis. Loss of FH function was associated with heightened inflammation and exacerbated tubulointerstitial damage in the kidney. Moreover, FH deficiency aggravated fibrosis in both the liver and lungs. Mechanistically, the depletion of FH in renal tubular cells led to fumarate accumulation. Fumarate directly succinated A-kinase anchoring protein 12 at cysteine 670, thereby diminishing its capacity to inhibit the activity of protein kinase Cζ (PKCζ). This process exacerbated renal inflammation and fibrosis by activating the downstream PKCζ/NF-κB and PKCζ/β-catenin pathways. Additionally, the upregulation of FH through adeno-associated virus 2/9–mediated FH overexpression markedly mitigated renal inflammation and fibrosis. These findings highlighted the important role of fumarate accumulation in the advancement of renal fibrosis, supporting FH as a potential therapeutic target in renal fibrosis.

## Abstract 2

A buildup of fumarate drives kidney inflammation and scarring, and restoring fumarate hydratase may help identify new treatment strategies for chronic kidney disease.

## Graphical abstract

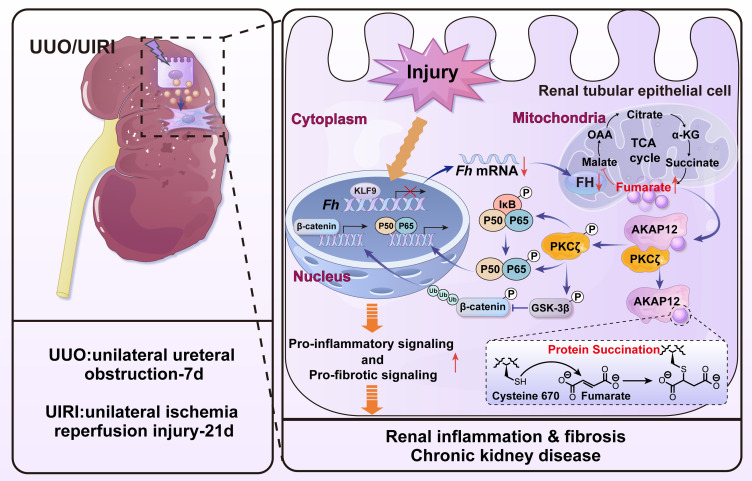

## Introduction

Chronic kidney disease (CKD) is characterized by a progressive impairment of renal structure and function, resulting from various etiologies and posing a substantial threat to public health worldwide (1, 2). Approximately 10% of the global population is affected by CKD, and it is projected to become the fifth leading cause of death globally by 2040 (3, 4). Many patients with CKD ultimately progress to end-stage renal disease, necessitating renal replacement therapy or kidney transplantation, which places a heavy burden on families and society (5). Renal fibrosis is a common pathological feature and the final manifestation of CKD (6–8). Previous studies have shown that the activation and persistence of inflammation following damage to tubular epithelial cells (TECs) contribute to the progression of renal fibrosis (9–11). Despite extensive exploration, the specific mechanisms remain unclear. Therefore, a deeper investigation into the mechanisms of inflammation activation and inadequate repair during renal fibrosis may provide promising treatment strategies to prevent or reverse kidney fibrosis.

Different from the previous perception that certain tricarboxylic acid (TCA) cycle metabolites primarily serve as intermediates in biosynthesis and energy supply, these metabolites also exert substantial biological effects in immune regulation and inflammatory responses by modulating transcription factors, modifying proteins, and altering cellular and molecular signaling pathways (12). Notable examples include succinate, which accumulates in the extracellular space and exhibits a pro-inflammatory role, thereby promoting liver fibrosis (13). In fibrotic kidneys, metabolic reprogramming in tubular cells leads to an energetic crisis and mitochondrial dysfunction, resulting in the abnormal accumulation of large quantities of metabolites (14, 15). However, the complex interplay between metabolite accumulation and inflammation regulation, as well as its implications for kidney fibrosis, remain largely unexplored.

Fumarate hydratase (FH) is an enzyme that plays multiple roles in the mitochondrial TCA cycle, the purine nucleotide cycle, DNA damage repair, and tumor suppression (16–18). In the TCA cycle, FH catalyzes the reversible conversion of fumarate to malate, and a deficiency in FH results in the abnormal accumulation of fumarate within cells (19). Loss-of-function mutations in FH are associated with hereditary leiomyomatosis and renal cell carcinoma (20). The role of fumarate as a modulator of inflammation has garnered increasing attention, with several studies highlighting its substantial involvement in innate immunity and inflammatory responses (21). In renal tubular cells, elevated levels of intracellular fumarate promote the release of mitochondrial DNA (mtDNA) into the cytoplasm, thereby activating the innate immune response (22). Clinical studies have shown that urinary fumarate levels are elevated in those with progressive CKD and may independently predict CKD progression beyond traditional cardiorenal risk factors (23). Moreover, in a mouse model of diabetic kidney disease, fumarate accumulated in both urine and kidney cortex. Treatment with a NOX1/NOX4 inhibitor increased FH expression, reduced fumarate accumulation, and attenuated diabetic glomerular dysfunction (24). However, the precise functions and mechanisms of fumarate in renal inflammation and fibrosis remain unclear.

In this study, we found that FH expression was reduced in fibrotic kidneys and was negatively correlated with CKD severity and the degree of renal fibrosis. In TECs, FH deficiency led to the accumulation of fumarate in the cytoplasm, which subsequently succinated A-kinase anchoring protein 12 (AKAP12) at cysteine 670. This process promoted the release and activation of protein kinase Cζ (PKCζ), thereby exacerbating renal inflammation and fibrosis through the PKCζ/NF-κB and PKCζ/β-catenin pathways. In preclinical models, adeno-associated virus 2/9–mediated (AAV2/9-mediated) FH overexpression markedly attenuated renal inflammation and fibrosis. We further found that FH deficiency promoted fibrosis in other organs. Together, these findings highlighted an important role for aberrant fumarate accumulation in the progression of renal inflammation and fibrosis and supported FH as a potential therapeutic target in renal fibrosis.

## Results

### Reduced FH expression is associated with renal fibrosis severity and kidney dysfunction.

**Figure 1:**
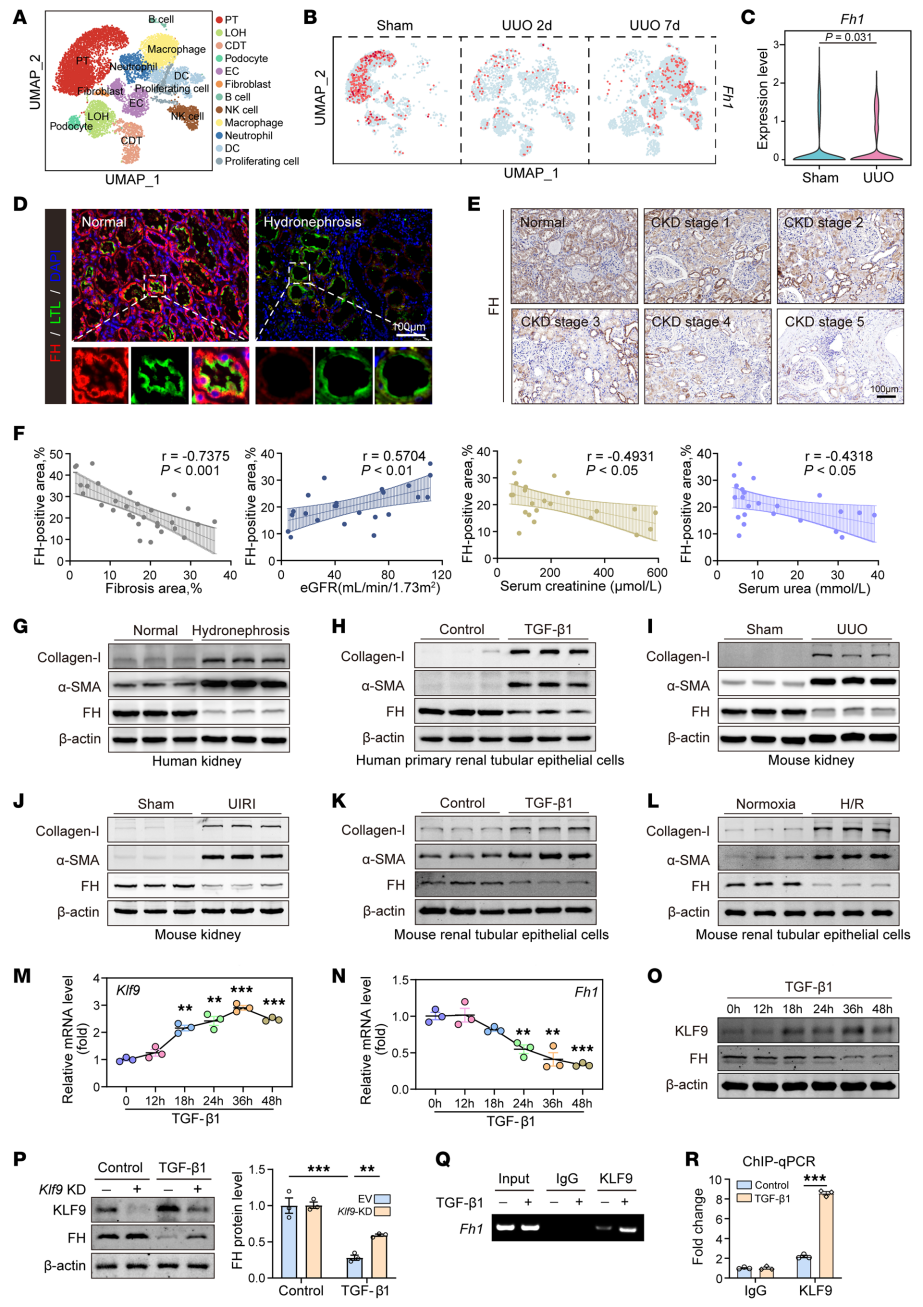
FH expression is reduced in CKD and is associated with KLF9-mediated repression. (**A**) Uniform manifold approximation and projection plot showing distinct renal cell clusters in the public single-cell RNA-seq dataset NCBI Gene Expression Omnibus (GEO) GSE140023. PT, proximal tubule; LOH, loop of Henle; CDT, convoluted distal tubule. (**B** and **C**) Feature plot and violin plot showing *Fh1* expression in renal proximal TECs under sham, UUO 2-day, and UUO 7-day conditions. (**D**) Representative IF staining of FH and LTL in normal kidney and hydronephrotic kidney tissue. Scale bar, 100 μm. (**E**) Representative IHC staining of FH in human kidney tissue from healthy controls and patients with CKD stages 1–5 (*n* = 30). Scale bar, 100 μm. (**F**) Correlation analyses showing negative associations of FH-positive area with renal fibrosis area, serum urea, and serum creatinine, and a positive association with eGFR in human kidney samples (*n* = 23/30). (**G**) Western blot analysis of FH, α–smooth muscle actin (α-SMA), and collagen I in kidney tissues from healthy controls and patients with hydronephrosis (*n* = 6). (**H**) Western blot analysis of α-SMA and collagen I in primary human renal TECs after TGF-β1 stimulation (*n* = 3). (**I** and **J**) Western blot analysis of FH, α-SMA, and collagen I in UUO and UIRI mouse models (*n* = 6). (**K** and **L**) Western blot analysis of FH, α-SMA, and collagen I in mTECs subjected to TGF-β1 or H/R treatment (*n* = 3). (**M** and **N**) qRT-PCR analysis of *Klf9* and *Fh1* mRNA expression at the indicated time points after TGF-β1 treatment (*n* = 3). (**O**) Western blot analysis of FH and KLF9 protein expression at the indicated time points after TGF-β1 treatment (*n* = 3). (**P**) Western blot analysis of FH protein expression after *Klf9* knockdown (KD) in TGF-β1–stimulated mTECs (*n* = 3). (**Q** and **R**) ChIP assay showing increased KLF9 occupancy at the upstream regulatory region of *Fh1* after TGF-β1 stimulation (*n* = 3). Data are shown as mean ± SEM. Statistical analysis was performed using Spearman’s correlation analysis (**F**), 1-way ANOVA followed by Tukey’s multiple comparisons test (**M** and **N**), and 2-way ANOVA followed by Tukey’s multiple comparisons test (**P** and **R**). **P < 0.01, and ***P < 0.001.

To investigate the involvement of FH in renal fibrosis, we initially conducted single-cell RNA-sequencing (RNA-seq) analysis using data from the NCBI Gene Expression Omnibus (GEO) database. Our analysis revealed that FH was downregulated in TECs in the unilateral ureteral obstruction (UUO) mouse model (Figure 1, A–C). To define the localization of FH expression in kidney tissue, we performed double immunofluorescence (IF) staining for FH and Lotus tetragonolobus lectin (LTL), a marker of proximal tubules. FH was predominantly expressed in proximal TECs and was reduced in kidney tissue from patients with hydronephrosis (Figure 1D). Immunohistochemistry (IHC) and Western blot analyses further showed that FH expression was markedly reduced in the kidney tissue from patients with hydronephrosis and CKD (Figure 1, E and G, and Supplemental Figure 1A; supplemental material available online with this article; https://doi.org/10.1172/JCI200755DS1). FH expression was positively correlated with the estimated glomerular filtration rate (eGFR) and negatively correlated with serum urea and creatinine levels, as well as the degree of renal fibrosis (Figure 1F and Supplemental Figure 1B).

Subsequently, we isolated primary human renal TECs from normal kidney tissue and found that TGF-β1 treatment reduced FH expression (Figure 1H and Supplemental Figure 1C). FH protein expression was also reduced in mouse models of renal fibrosis induced by UUO or unilateral ischemia/reperfusion injury (UIRI) (Figure 1, I and J, and Supplemental Figure 1, D–G). Similarly, in vitro fibrosis models induced by TGF-β1 or hypoxia/reoxygenation (H/R) showed reduced FH protein levels (Figure 1, K and L, and Supplemental Figure 1, H–J).

To identify candidate transcription factors potentially involved in *Fh1* regulation, we performed an exploratory inspection of the genomic region upstream of the mouse *Fh1* transcription start site using publicly available annotations in the UCSC Genome Browser. This inspection showed multiple annotated transcription factor–associated motifs across the upstream regulatory region (Supplemental Figure 2A). Among the annotated candidate transcription factors, B-cell lymphoma 6 (BCL6), paired box 3 (PAX3), and Krüppel-like factor 9 (KLF9) were selected for further analysis based on published evidence linking them to renal pathophysiology. Quantitative reverse transcription polymerase chain reaction (qRT-PCR) analysis conducted in both UUO and UIRI mouse models demonstrated that *Bcl6* expression was downregulated, *Pax3* expression remained unchanged, and *Klf9* expression was upregulated under injury conditions (Supplemental Figure 2, B–D). To prioritize these candidates functionally, we performed siRNA-mediated knockdown (KD) of *Bcl6* and *Klf9* in TGF-β1–treated TECs. Silencing *Bcl6* had no detectable effect on *Fh1* expression, whereas *Klf9* KD partially restored both FH protein and *Fh1* mRNA levels in the injured cells (Figure 1P and Supplemental Figure 2, E and F), supporting prioritization of KLF9 for further study.

KLF9 is a transcriptional repressor that can inhibit gene transcription by binding to GC-rich promoter elements (25, 26). In a TGF-β1–stimulated in vitro model, time course analyses demonstrated that *Klf9* mRNA and KLF9 protein levels increased before the decline in FH expression (Figure 1, M–O, and Supplemental Figure 2G), consistent with an upstream regulatory role. Consistently, single-cell RNA-seq analysis of UUO kidneys revealed increased *Klf9* expression specifically in TECs during fibrotic progression (Supplemental Figure 2, I and J), which was confirmed by increased KLF9 expression in fibrotic kidneys from UUO mice (Supplemental Figure 2H). Finally, chromatin immunoprecipitation (ChIP) assays demonstrated enhanced KLF9 occupancy at the upstream regulatory region of *Fh1* in fibrotic kidneys (Figure 1, Q and R), supporting a role for KLF9 as a transcriptional regulator of *Fh1* during renal fibrosis. In parallel, Western blot analyses revealed that KLF9 KD reduced the expression of multiple fibrosis- and inflammation-related markers (Supplemental Figure 2K), indicating that KLF9 plays a functional role in regulating renal inflammatory and fibrotic responses.

### FH deficiency exacerbates UUO- and UIRI-induced renal injury and fibrosis.

**Figure 2:**
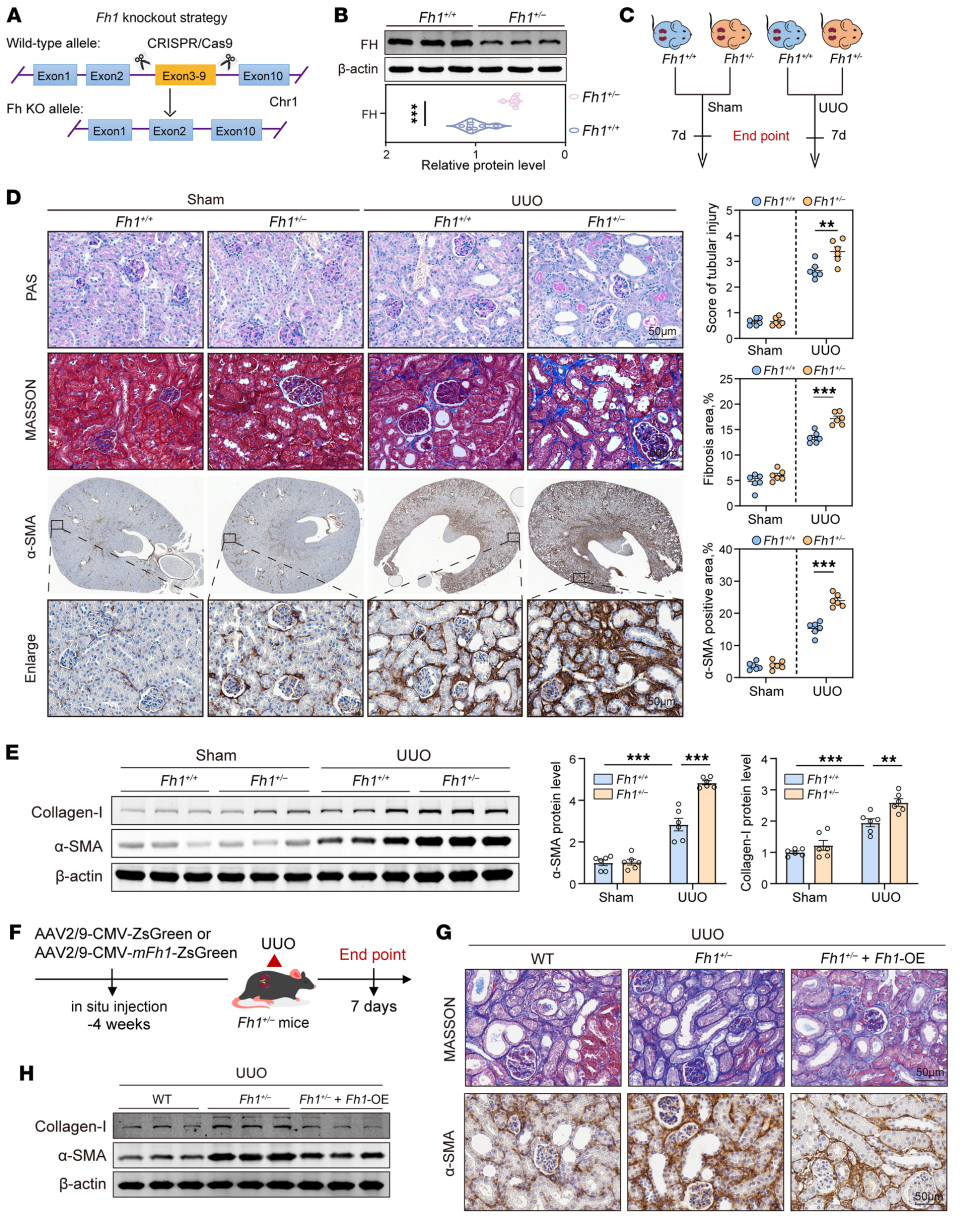
*Fh1^+/–^* mice show exacerbated renal injury and fibrosis. (**A**) Schematic illustration of the *Fh1*-knockout strategy. *Fh1*^+/–^, *Fh1* heterozygous. (**B**) Western blot analysis of FH in kidneys from *Fh1^+/+^* and *Fh1*^+/–^ mice. (**C**) Experimental design of the UUO model. (**D**) Representative PAS, Masson’s trichrome, and α-SMA IHC staining of kidneys from *Fh1^+/+^* and *Fh1*^+/–^ mice subjected to sham operation or UUO (*n* = 6). Scale bars, 50 μm. (**E**) Western blot analysis of α-SMA and collagen I in kidneys from *Fh1^+/+^* and *Fh1^+/–^* mice after sham operation or UUO (*n* = 6). (**F**) Experimental design for FH overexpression (OE) rescue in *Fh1^+/–^* mice. (**G**) Representative Masson’s trichrome and α-SMA IHC staining of kidneys from WT mice, *Fh1^+/–^* mice, and *Fh1^+/–^* mice with FH OE after UUO (*n* = 6). Scale bar, 50 μm. (**H**) Western blot analysis of α-SMA and collagen I in kidneys from WT mice, *Fh1^+/–^* mice, and *Fh1^+/–^* mice with FH OE after UUO (*n* = 6). Data are shown as mean ± SEM. Statistical analysis was performed using 2-tailed unpaired Student’s *t* test (**B**) and 2-way ANOVA followed by Tukey’s multiple comparisons test (**D** and **E**). **P < 0.01, and ***P < 0.001.

To investigate the functional role of FH in renal fibrosis, we generated *Fh1*-knockout mice using a CRISPR/Cas9 genome-editing strategy targeting exons 3–9 of *Fh1* (Figure 2A). Successful targeting was confirmed by PCR-based genotyping and agarose gel electrophoresis (Supplemental Figure 3, A and B). Consistent with Mouse Genome Informatics annotations, homozygous deletion of *Fh1* resulted in early embryonic lethality, with embryos failing to progress beyond the egg cylinder stage (approximately embryonic day 6.0). Therefore, all subsequent experiments were performed using *Fh1^+/–^* mice. Assessment of baseline renal phenotypes revealed no significant differences in renal function or histological features between *Fh1^+/–^* mice and their wild-type (WT) littermates (Supplemental Figure 3, D–F). In contrast, IHC staining and Western blot analyses confirmed a marked reduction in FH protein expression in the kidneys of *Fh1^+/–^* mice compared with WT controls (Figure 2B and Supplemental Figure 3C).

To assess the impact of the *Fh1* deficiency on renal injury and fibrosis, we established a 7-day UUO model (Figure 2C). Periodic acid–Schiff (PAS) and Masson’s trichrome staining showed that renal injury and fibrosis were more pronounced in *Fh1^+/–^* mice compared with WT littermates (Figure 2D). IHC staining and Western blot analyses demonstrated increased expression of α-SMA and collagen I in *Fh1^+/–^* mice (Figure 2, D and E, and Supplemental Figure 3G), confirming that the absence of FH aggravated UUO-induced fibrosis. Additionally, we conducted a 21-day UIRI study in *Fh1^+/–^* mice and observed similar results. *Fh1* deficiency exacerbated UIRI-induced kidney injury and fibrosis (Supplemental Figure 3H and Supplemental Figure 4A). To determine whether restoration of FH could rescue this phenotype, we utilized AAV2/9-mediated FH overexpression to restore FH expression in *Fh1^+/–^* mice and then induced UUO (Figure 2F). PAS, Masson’s trichrome, and IHC staining showed that restoration of FH reduced renal injury and fibrosis (Figure 2G and Supplemental Figure 4B). Western blot analyses further supported these findings (Figure 2H and Supplemental Figure 4C).

### Proximal tubule–specific Fh1 conditional knockin attenuates UUO- and UIRI-induced renal injury and fibrosis.

**Figure 3:**
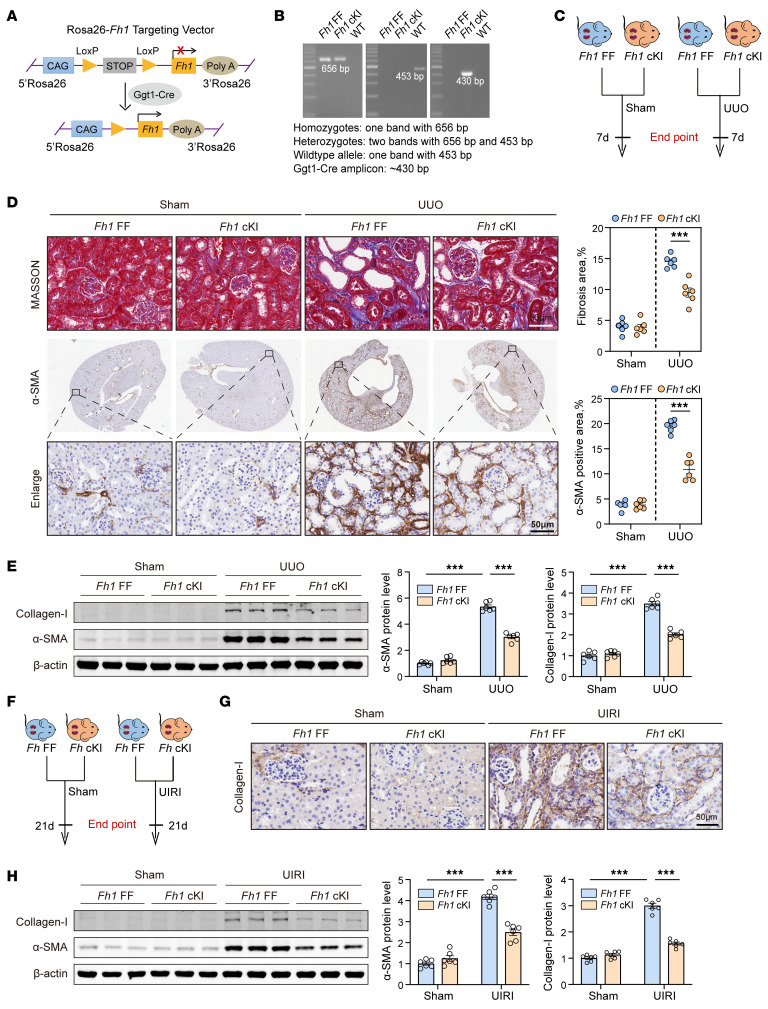
Conditional knockin of *Fh1* in renal proximal tubules attenuates renal injury and fibrosis. (**A**) Schematic illustration of the conditional knockin (cKI) strategy for *Fh1* expression in renal proximal tubules. (**B**) PCR-based genotyping of mice by agarose gel electrophoresis. (**C**) Experimental design for the UUO model. (**D**) Representative Masson’s trichrome and α-SMA IHC staining of kidneys from *Fh1* FF and *Fh1* cKI mice subjected to sham operation or UUO (*n* = 6). Scale bar, 50 μm. (**E**) Western blot analysis of α-SMA and collagen I in kidneys from *Fh1* FF and *Fh1* cKI mice after sham operation or UUO (*n* = 6). (**F**) Experimental design of the UIRI model. (**G**) Representative collagen I IHC staining of kidneys from *Fh1* FF and *Fh1* cKI mice subjected to sham surgery or UIRI (*n* = 6). Scale bar, 50 μm. (**H**) Western blot analysis of α-SMA and collagen I in kidneys from *Fh1* FF and *Fh1* cKI mice after sham operation or UIRI (*n* = 6). Data are shown as mean ± SEM. Statistical analysis was performed using 2-way ANOVA followed by Tukey’s multiple comparisons test (**D**, **E**, and **H**). ***P < 0.001.

To further elucidate the role of FH in proximal TECs during renal fibrosis, we generated a proximal tubule–specific *Fh1* conditional knockin (cKI) mouse model (Figure 3A). In this model, *Fh1* FF mice carried a homozygous ROSA26-targeted *Fh1* knock-in allele but did not express the transgene in the absence of Cre recombinase. In contrast, *Fh1* cKI mice expressed Ggt1-Cre, enabling proximal TEC-specific activation of the knock-in *Fh1* allele. All mice were genotyped using PCR followed by agarose gel electrophoresis (Figure 3B and Supplemental Figure 5A). IHC staining and Western blot analyses further confirmed successful generation of the *Fh1* cKI mice (Supplemental Figure 5, B and C). Notably, Western blot revealed 2 FH protein bands: one corresponding to endogenous FH and the other to the 3×FLAG-tagged knock-in FH, consistent with the design of the cKI construct. Importantly, UUO markedly reduced FH protein levels in *Fh1* FF kidneys. In contrast, FH expression in *Fh1* cKI kidneys remained relatively preserved after injury compared with littermate controls, despite the overall suppressive effect of UUO on FH expression (Supplemental Figure 5D).

In *Fh1* cKI mice, UUO-induced tubular injury and extracellular collagen deposition were attenuated compared with *Fh1* FF mice, as shown by hematoxylin and eosin (H&E) and Masson’s trichrome staining (Figure 3, C and D, and Supplemental Figure 5E). Western blot and IHC analyses further showed reduced expression of α-SMA and collagen I in *Fh1* cKI mice following UUO (Figure 3, D and E, and Supplemental Figure 5E). We next extended these findings to the UIRI model (Figure 3F). Consistently, proximal tubule–specific *Fh1* cKI attenuated UIRI-induced kidney injury and fibrosis (Figure 3, G and H, and Supplemental Figure 6A).

### FH regulates fibrotic responses in TGF-β1– and H/R-treated TECs.

TEC injury plays a crucial role in the pathogenesis of CKD (27). To validate the in vivo findings, we used siRNA transfection to silence *Fh1* in murine tubular epithelial cells (mTECs) (Supplemental Figure 7A). qRT-PCR analysis and Western blot analyses confirmed that *Fh1* siRNA reduced both *Fh1* mRNA and FH protein levels (Supplemental Figure 7, B and C). Following TGF-β1 treatment, *Acta2* and *Col1a1* mRNA levels were increased in *Fh1*-KD cells compared with those transfected with control siRNA (Supplemental Figure 7D). Western blot and IF staining further showed that *Fh1* KD increased α-SMA and collagen I protein expression (Supplemental Figure 7, E and F). Similar results were observed in the H/R-induced fibrogenesis model in mTECs (Supplemental Figure 7, G and H), supporting a role for *Fh1* KD in promoting renal fibrogenic responses under TGF-β1 and H/R stimulation.

Subsequently, we overexpressed *Fh1* in mTECs by plasmid transfection (Supplemental Figure 8A). qRT-PCR and Western blot analyses confirmed that *Fh1* OE increased *Fh1* mRNA and FH protein levels (Supplemental Figure 8, B and C). In parallel, qRT-PCR, Western blot, and IF staining showed that *Fh1* OE reduced *α-SMA* and *collagen I* expression in TGF-β1– and H/R-stimulated mTECs (Supplemental Figure 8, D–H). We next treated the cells with an FH inhibitor (Supplemental Figure 9A). Compared with cells treated with TGF-β1 alone, FH inhibitor treatment increased α-SMA and collagen I protein expression (Supplemental Figure 9, B and C).

### FH deficiency promotes renal inflammation.

**Figure 4:**
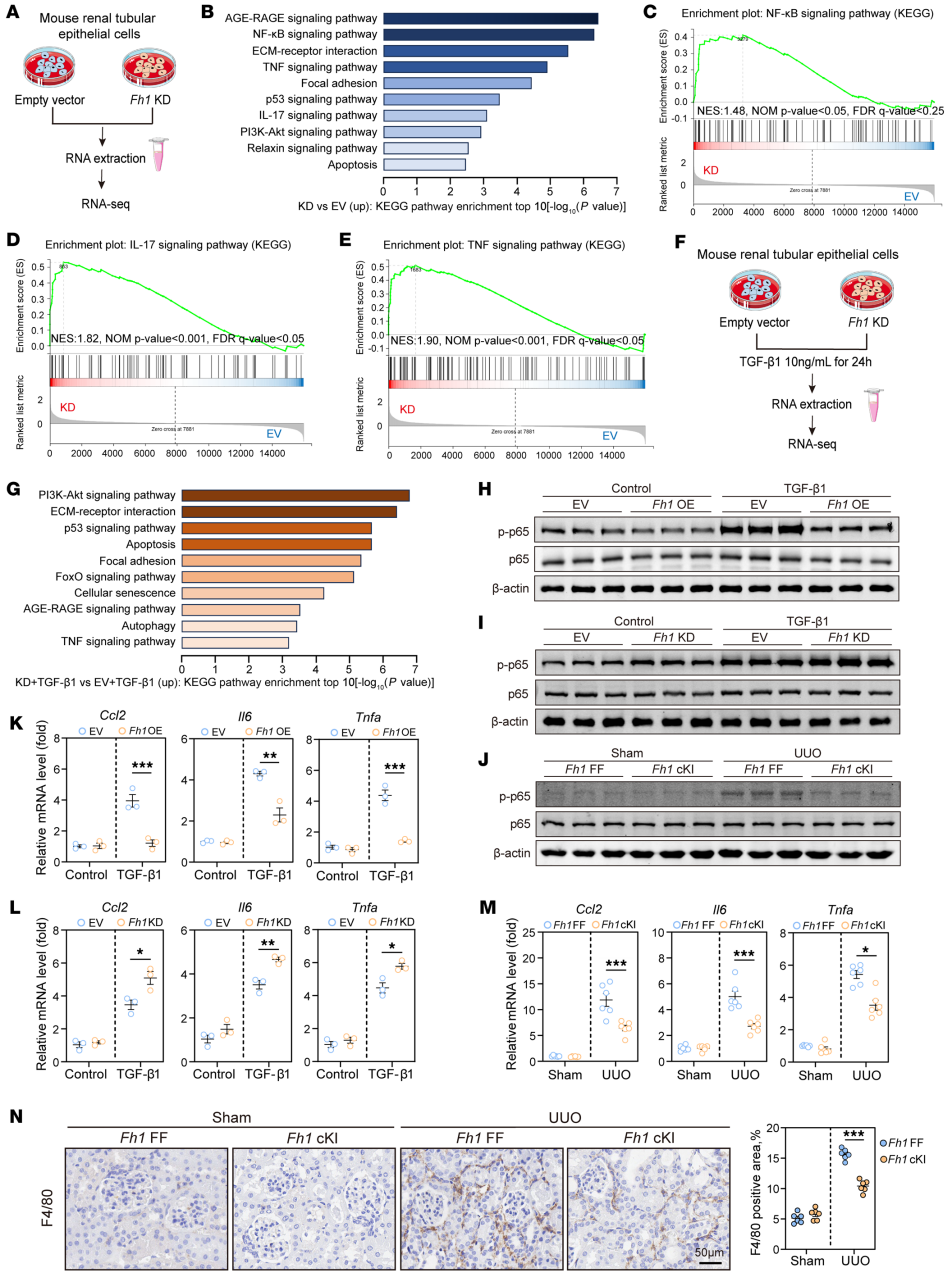
FH deficiency exacerbates renal inflammatory responses. (**A**) Schematic overview of the RNA-seq workflow. (**B**) KEGG pathway enrichment analysis showing the top 10 upregulated pathways in *Fh1*-KD cells. (**C**–**E**) GSEA showing enrichment of NF-κB, IL-17, and TNF, signaling pathways in *Fh1*-KD cells. (**F**) Schematic overview of the RNA-seq workflow following TGF-β1 stimulation. (**G**) KEGG pathway enrichment analysis showing the top 10 upregulated pathways in *Fh1*-KD cells after TGF-β1 treatment. (**H** and **I**) Western blot analysis of p-p65 in cells transfected with empty vector (EV), *Fh1*-OE, and *Fh1*-KD cells with or without TGF-β1 stimulation (*n* = 3). (**J**) Western blot analysis of p-p65 in kidneys from *Fh1* FF and *Fh1* cKI mice subjected to sham operation or UUO (*n* = 6). (**K**–**M**) qRT-PCR analysis of inflammatory gene expression in EV, *Fh1*-OE, and *Fh1*-KD cells with or without TGF-β1 treatment (*n* = 3) and in kidneys from *Fh1* FF and *Fh1* cKI mice after sham operation or UUO (*n* = 6). (**N**) Representative F4/80 IHC staining of kidneys from *Fh1* FF and *Fh1* cKI mice subjected to sham operation or UUO (*n* = 6). Scale bar, 50 μm. Data are shown as mean ± SEM. Statistical analysis was performed using 2-way ANOVA followed by Tukey’s multiple comparisons test (**K**–**N**). *P < 0.05, **P < 0.01, and ***P < 0.001.

Recent studies have highlighted an important role for FH in innate immunity and inflammation (21, 22, 28). To investigate the mechanisms through which FH influences renal fibrosis, we conducted RNA-seq analysis (Figure 4A). Kyoto Encyclopedia of Genes and Genomes (KEGG) enrichment analysis and Gene Set Enrichment Analysis (GSEA) showed that FH deficiency was associated with enrichment of inflammatory pathways, including NF-κB, tumor necrosis factor (TNF), and interleukin-17 (IL-17) signaling (Figure 4, B–E). We next performed RNA-seq in *Fh1*-KD cells treated with TGF-β1 (Figure 4F). KEGG enrichment analysis showed that pathways upregulated following *Fh1* KD in the fibrotic cell model were predominantly inflammatory, including PI3K/AKT and TNF signaling (Figure 4G and Supplemental Figure 10A). Together, these findings supported a role for FH loss in promoting inflammatory pathway activation during renal fibrosis.

To further examine the effect of FH on inflammatory signaling, we performed Western blot, which showed that *Fh1* OE reduced NF-κB p65 phosphorylation (Figure 4H and Supplemental Figure 10B). Consistent with these findings, qRT-PCR analysis revealed that chemokine ligand 2 (*Ccl2*), *Il6*, and *Tnfa* mRNA levels were reduced in *Fh1*-OE cells (Figure 4K). Conversely, *Fh1* KD increased NF-κB p65 phosphorylation and the expression of pro-inflammatory genes, including *Ccl2*, *Il6*, and *Tnfa* (Figure 4, I and L, and Supplemental Figure 10C). In the UUO model, *Fh1* cKI mice showed reduced macrophage infiltration in the renal interstitium, decreased p65 phosphorylation, and lower expression of inflammatory genes (Figures 4, J, M and N, and Supplemental Figure 10D). In contrast, *Fh1^+/–^* mice showed increased macrophage infiltration and inflammatory cytokine expression (Supplemental Figure 10, E and F).

### Fumarate accumulation promotes renal inflammation and fibrosis.

**Figure 5:**
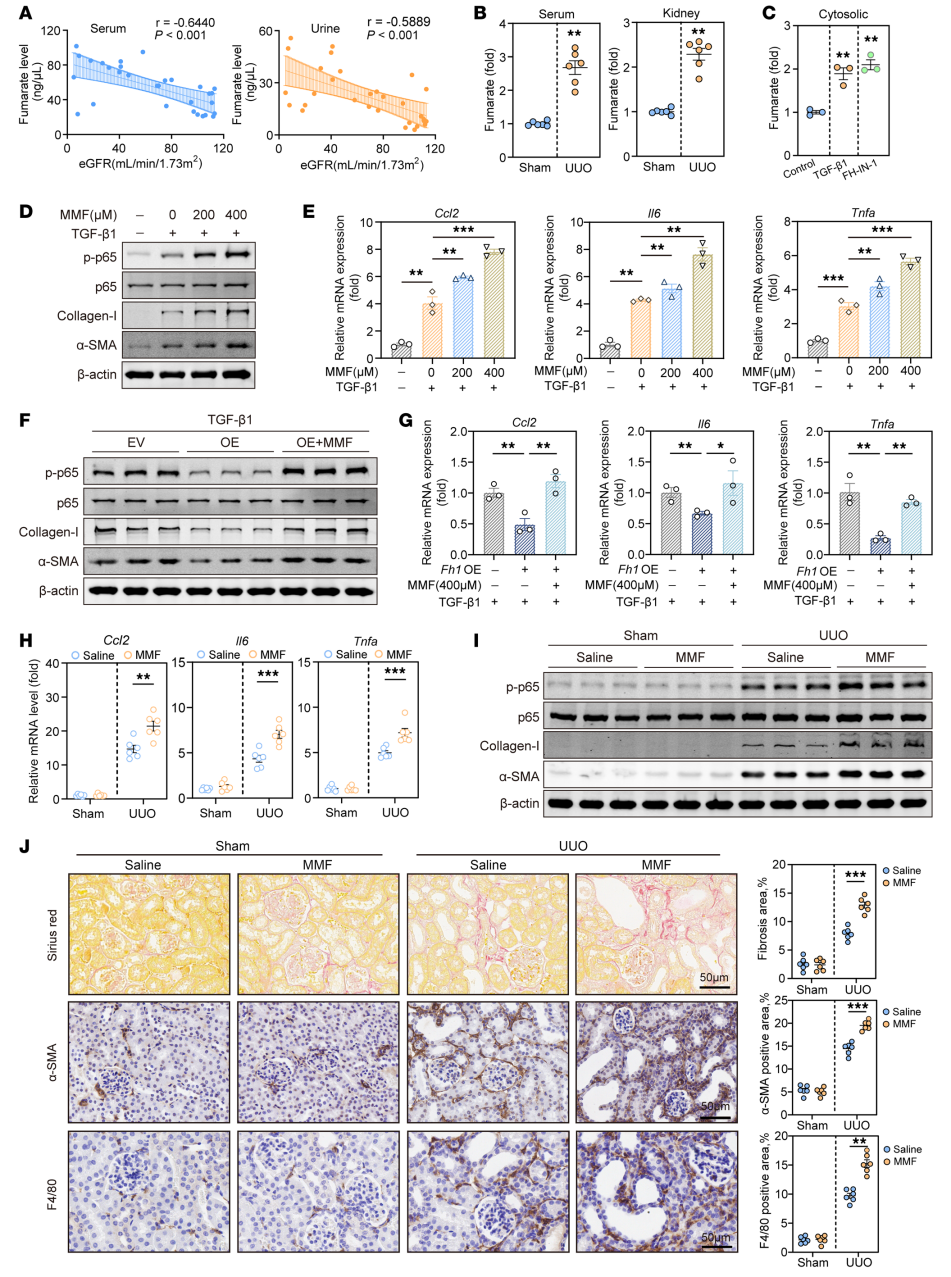
Fumarate accumulation promotes renal inflammation and fibrosis. (**A**) Spearman’s correlation analysis showing negative correlations between serum fumarate and eGFR (*n* = 30, *r* = –0.6440, *P* < 0.001) and between urinary fumarate and eGFR (*n* = 30, *r* = –0.5889, *P* < 0.001). (**B**) Fumarate levels in kidneys and serum from mice subjected to sham operation or UUO (*n* = 6). (**C**) Cystolic fumarate levels in mTECs treated with control, TGF-β1, or FH-IN-1 (*n* = 3). (**D**) Western blot analysis of α-SMA, collagen I, and p-p65 in cells treated with increasing concentrations of MMF with or without TGF-β1 stimulation (*n* = 3). (**E**) qRT-PCR analysis of *Ccl2*, *Il6*, and *Tnfa* expression under the same conditions as in **D** (*n* = 3). (**F**) Western blot analysis of α-SMA, collagen I, and p-p65 in EV, *Fh1*-OE, and *Fh1*-OE + MMF groups after TGF-β1 stimulation (*n* = 3). (**G**) qRT-PCR analysis of inflammatory gene expression in the same groups as in **F** (*n* = 3). (**H**) qRT-PCR analysis of inflammatory gene expression in kidneys from control and MMF-treated mice subjected to sham operation or UUO (*n* = 6). (**I**) Western blot analysis of α-SMA, collagen I, and p-p65 in kidneys from control and MMF-treated mice after sham operation or UUO (*n* = 6). (**J**) Representative Sirius red staining and IHC staining for α-SMA and F4/80 in kidneys from control and MMF-treated mice after sham operation or UUO (*n* = 6). Scale bar, 50 μm. Data are shown as mean ± SEM. Statistical analysis was performed using Spearman’s correlation analysis (**A**), 2-tailed unpaired Student’s *t* test (**B**), 1-way ANOVA followed by Tukey’s multiple comparisons test (**C**, **E**, and **G**), and 2-way ANOVA followed by Tukey’s multiple comparisons test (**H** and **J**). *P < 0.05, **P < 0.01, and ***P < 0.001.

FH catalyzes the reversible conversion of fumarate to malate (16). To determine whether FH deficiency was associated with fumarate accumulation during renal fibrosis, we measured fumarate levels in human samples and experimental models. In patients with CKD, serum and urinary fumarate levels were inversely correlated with eGFR (Figure 5A). Fumarate levels were also increased in the kidneys and serum of UUO mice (Figure 5B), as well as in an in vitro fibrosis model (Figure 5C). To test whether fumarate accumulation promoted renal inflammation and fibrosis, we treated cells with monomethyl fumarate (MMF), a cell-permeable fumarate derivative that increases intracellular fumarate levels without markedly altering its reactivity (22). In the fibrotic setting, MMF treatment increased α-SMA and collagen I protein expression and enhanced NF-κB p65 activation (Figure 5D and Supplemental Figure 11A). qRT-PCR analysis further showed that the mRNA levels of inflammatory genes increased with MMF treatment (Figure 5E). In TGF-β1–treated cells, *Fh1* OE reduced inflammatory gene expression, whereas MMF administration reversed this effect (Figure 5G). Western blot analyses further showed similar results (Figure 5F and Supplemental Figure 11B). In vivo, mice administered MMF and then subjected to UUO developed more severe renal fibrosis and inflammatory responses than saline-treated controls, as shown by PAS, Sirius red, and IHC staining, as well as Western blot and qRT-PCR analyses (Figure 5, H–J, and Supplemental Figure 11, C–E). Together, these findings indicated that fumarate accumulation resulting from FH deficiency promoted renal inflammation and fibrosis.

### Fumarate directly succinates AKAP12 at cysteine 670.

**Figure 6:**
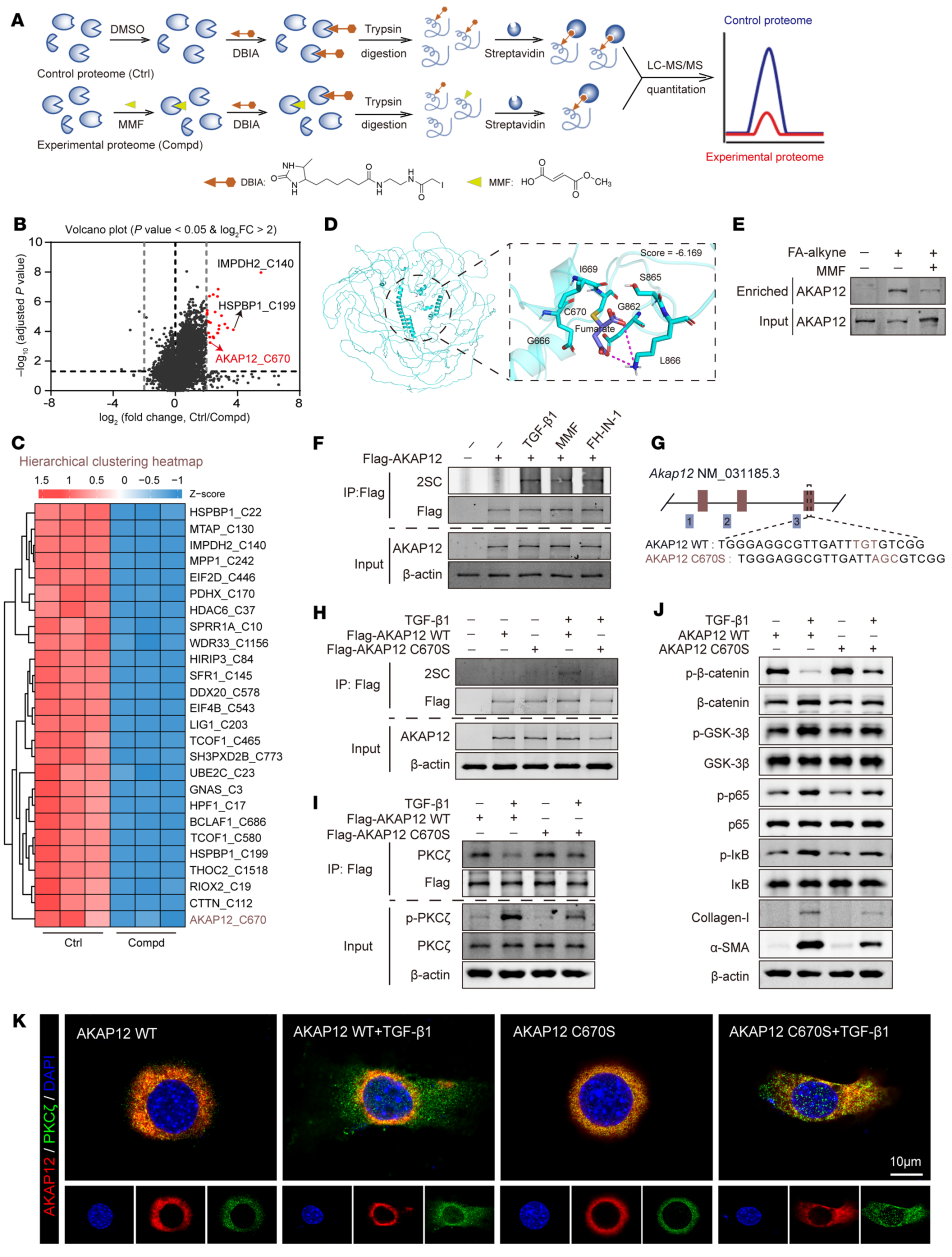
Fumarate promotes renal inflammation and fibrosis through succination of AKAP12 at C670. (**A**) Schematic illustration of the in vitro experimental design. (**B**) Volcano plot showing altered proteins and cysteine sites (Ctrl / Compd > 4, *P* < 0.05). Ctrl, control proteome; Compd, MMF-treated experimental proteome. (**C**) Hierarchical clustering heatmap showing differentially modified proteins and cysteine sites between control and fumarate-treated groups. (**D**) Molecular docking model predicting the interaction of fumarate with AKAP12 at C670 via Michael addition. (**E**) Western blot analysis showing reduced AKAP12 recovery in the FA-alkyne pulldown after MMF treatment (*n* = 3). (**F**) Western blot analysis of AKAP12 succination in cells treated with control, TGF-β1, MMF, or FH-IN-1 (*n* = 3). (**G**) Schematic diagram of plasmid construction for AKAP12 site-directed mutagenesis. (**H**) Western blot analysis of AKAP12 succination in AKAP12 WT and AKAP12 C670S cells with or without TGF-β1 treatment (*n* = 3). (**I**) Co-IP and Western blot analysis of AKAP12-PKCζ interaction and PKCζ phosphorylation in AKAP12 WT and AKAP12 C670S cells with or without TGF-β1 treatment (*n* = 3). (**J**) Western blot analysis of α-SMA, collagen I, p-IκB, p-P65, p-GSK-3β, and p-β-catenin in AKAP12 WT and AKAP12 C670S cells with or without TGF-β1 treatment (*n* = 3). (**K**) Representative IF images showing AKAP12–PKCζ colocalization in AKAP12 WT and AKAP12 C670S cells under the indicated conditions (*n* = 3). Scale bar, 10 μm.

As an electrophile metabolite, fumarate can undergo Michael addition with nucleophilic cysteine residues on proteins, resulting in formation of S-(2-succinyl) cysteine (2SC), a modification known as succination (29, 30). IHC staining showed increased 2SC levels in kidneys from *Fh1*-deficient mice subjected to UUO (Supplemental Figure 12A). We next used a desthiobiotin-iodoacetamide (DBIA) probe in a competitive cysteine-labeling strategy, coupled with liquid chromatography-tandem mass spectrometry (LC-MS/MS), to identify candidate cysteine sites potentially modified by fumarate (Figure 6A). This analysis identified 26 high-confidence modification sites across 24 proteins (Figure 6, B and C). Based on prior RNA-seq results and their potential relevance to inflammatory signaling, IMPDH2, HSPBP1, and AKAP12 were prioritized for further analysis (Supplemental Figure 12, B–E). We then individually knocked down *Impdh2*, *Hspbp1*, and *Akap12* by siRNA transfection (Supplemental Figure 12F). Among these candidates, *Akap12* knockdown produced the most pronounced effect on fibrogenic responses in vitro (Supplemental Figure 12, G–I).

AKAP12, also known as Src-suppressed C-kinase substrate, is a member of the A-kinase anchoring protein family (31, 32). It serves as a scaffold that anchors protein kinases, phosphatases, and other signaling molecules to specific subcellular compartments, thereby regulating intracellular signaling (33–35). However, its function in renal fibrosis remains unclear. Western blot and IF staining showed that *Akap12* KD enhanced TGF-β1–induced expression of fibrotic markers (Supplemental Figure 12, J and K). Conversely, *Akap12* OE attenuated these effects (Supplemental Figure 13, A and B). Molecular docking analysis using Schrödinger software predicted that fumarate could interact with AKAP12 at C670 via a Michael addition reaction, with a docking score of –6.169, supporting the possibility of succination at this site (Figure 6D). Consistently, exogenous fumarate treatment reduced AKAP12 enrichment in the FA-alkyne pulldown (Figure 6E). Moreover, treatment with TGF-β1, MMF, or FH-IN-1 increased AKAP12 succination, as assessed by 2SC immunoblotting (Figure 6F). Mutation of AKAP12 C670 from cysteine to serine markedly reduced its succination (Figure 6, G and H).

### AKAP12 succination promotes renal inflammation and fibrosis through PKCζ/NF-κB and PKCζ/β-catenin signaling.

AKAP12 has been reported to interact with PKCζ and restrain its activation (36, 37). PKCζ can promote NF-κB signaling through phosphorylation of IκB and p65 (38, 39). It has also been implicated in regulation of the GSK-3β/β-catenin axis during renal fibrosis (40). Western blot analyses showed that *Fh1* KD in mTECs increased PKCζ phosphorylation and β-catenin expression, whereas *Fh1* OE attenuated these changes (Supplemental Figure 13, C and D). Similar changes were observed in *Fh1^+/–^* and *Fh1* cKI mice (Supplemental Figure 13, E and F). Consistent with our earlier findings, FH deficiency was also associated with activation of NF-κB signaling (Figure 4, H–M, and Supplemental Figure 10, B–E). Co-immunoprecipitation (co-IP) assays showed that TGF-β1 stimulation reduced the interaction between AKAP12 and PKCζ while increasing PKCζ phosphorylation, and C670S mutation of AKAP12 partially reversed these effects (Figure 6I and Supplemental Figure 14A). Double IF staining further supported these findings (Figure 6K). Moreover, C670S mutation reduced phosphorylation of IκB, p65 and GSK-3β; promoted phosphorylation and degradation of β-catenin; and reduced expression of α-SMA and collagen I, as well as the expression of inflammatory genes (Figure 6J and Supplemental Figure 14, B–D). Together, these findings support a mechanism in which succination of AKAP12 at C670 weakens its interaction with PKCζ, thereby attenuating the inhibitory effect of AKAP12 on PKCζ activation and promoting PKCζ/NF-κB and PKCζ/β-catenin signaling, ultimately enhancing renal inflammation and fibrosis.

### Akap12 C670S mutation attenuates UUO- and UIRI-induced renal inflammation and fibrosis.

**Figure 7:**
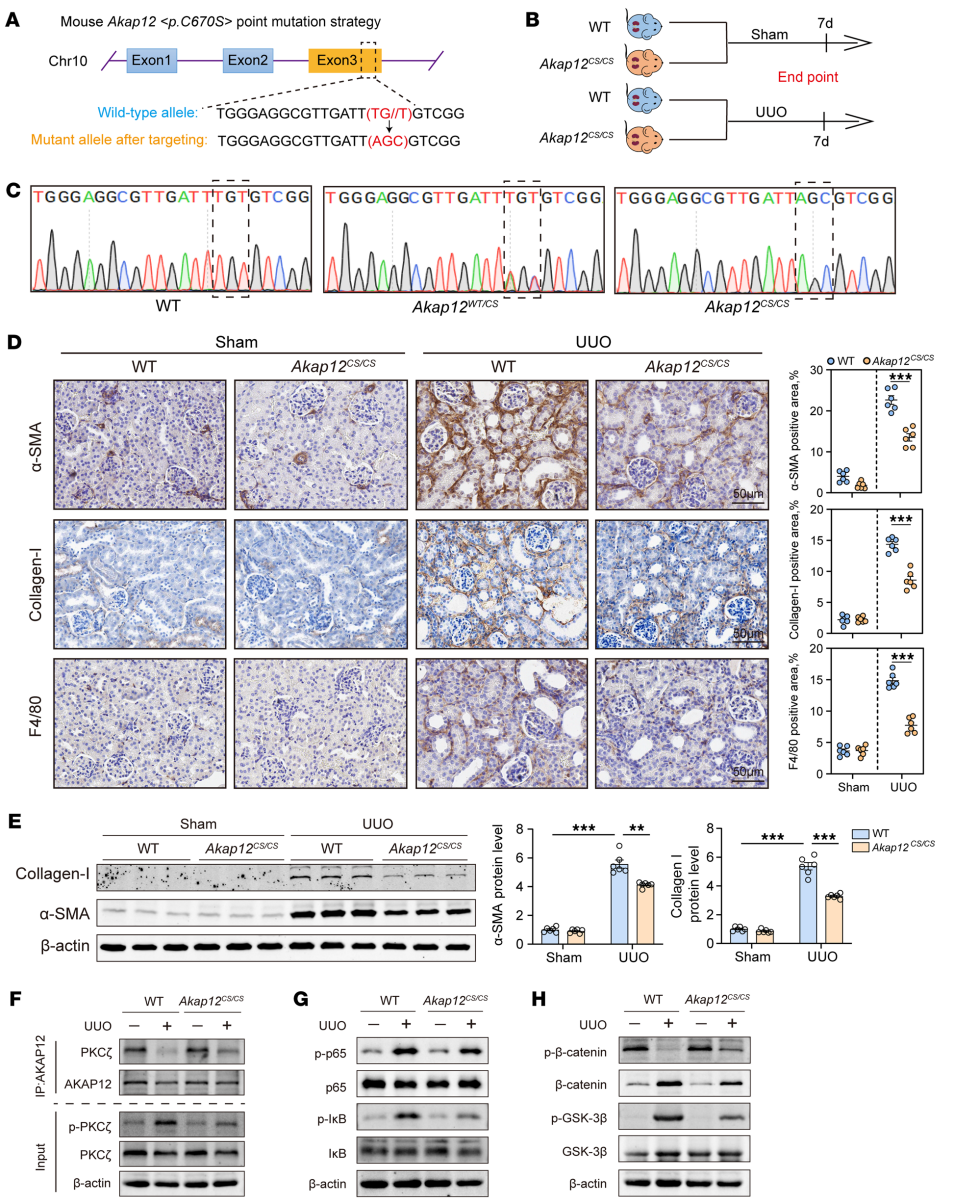
*Akap12^CS/CS^* mice are protected against UUO-induced renal inflammation and fibrosis. (**A**) Schematic illustration of the CRISPR/Cas9-mediated C670S mutation introduced into the mouse *Akap12* locus. (**B**) Experimental design of the UUO model. (**C**) Representative Sanger sequencing traces confirming the *Akap12^CS/CS^* genotype. (**D**) Representative IHC staining for α-SMA, collagen I, and F4/80 in kidneys from WT and *Akap12^CS/CS^* mice subjected to sham operation or UUO (*n* = 6). Scale bar, 50 μm. (**E**) Western blot analysis of α-SMA and collagen I in kidneys from WT and *Akap12^CS/CS^* mice after sham operation or UUO (*n* = 6). (**F**) Western blot and co-IP analyses of PKCζ phosphorylation and AKAP12-PKCζ interaction in kidneys from WT and *Akap12^CS/CS^* mice after sham operation or UUO (*n* = 6). (**G** and **H**) Western blot analysis of p-IκB, p-p65, p-GSK-3β, and p-β-catenin in kidneys from WT and *Akap12^CS/CS^* mice after sham operation or UUO (*n* = 6). Data are shown as mean ± SEM. Statistical analysis was performed using 2-way ANOVA followed by Tukey’s multiple comparisons test (**D** and **E**). **P < 0.01, and ***P < 0.001.

To further elucidate the functional role of the AKAP12 C670 site in renal inflammation and fibrosis, we generated a C57BL/6J mouse model harboring the *Akap12* C670S mutation, designated as *Akap12^CS/CS^*, using CRISPR/Cas9-mediated genome engineering (Figure 7, A and C). *Akap12^CS/CS^* mice showed no overt abnormalities compared with WT littermates. Assessment of baseline renal phenotypes revealed no significant differences in renal function or histological features between *Akap12^CS/CS^* mice and their WT littermates (Supplemental Figure 15, A–C).

Subsequently, we established a 7-day UUO model (Figure 7B). Compared with WT mice, *Akap12^CS/CS^* mice showed reduced renal injury, inflammation, and fibrosis, as shown by histological and molecular analyses (Figure 7, D and E, and Supplemental Figure 15, E and F). Immunoblot analysis revealed that AKAP12 protein levels were reduced following UUO-induced injury. Under identical injury conditions, AKAP12 protein abundance was comparable between *Akap12^CS/CS^* mice and WT littermates, suggesting that the C670S mutation did not affect overall AKAP12 expression (Supplemental Figure 15D). These findings suggest that the protective phenotype observed in *Akap12^CS/CS^* mice was not due to altered AKAP12 protein levels, but rather to the functional consequences of the C670S mutation. Similar results were observed in a 21-day UIRI model (Supplemental Figure 15, G and H, and Supplemental Figure 16, A and B). Co-IP analysis showed that UUO reduced the interaction between AKAP12 and PKCζ and increased PKCζ phosphorylation compared with sham controls. This injury-induced disruption of the AKAP12-PKCζ interaction was attenuated in *Akap12^CS/CS^* mice (Figure 7F and Supplemental Figure 16C). In parallel, *Akap12^CS/CS^* mice showed reduced phosphorylation of IκB, p65, and GSK-3β, together with enhanced phosphorylation and degradation of β-catenin, compared with WT mice subjected to UUO (Figure 7, G and H, and Supplemental Figure 16, D and E). Collectively, these findings indicate that the AKAP12 C670S mutation diminishes PKCζ/NF-κB and PKCζ/β-catenin signaling, thereby reducing UUO- and UIRI-induced renal inflammation and fibrosis.

### FH deficiency exacerbates liver and pulmonary fibrosis.

**Figure 8:**
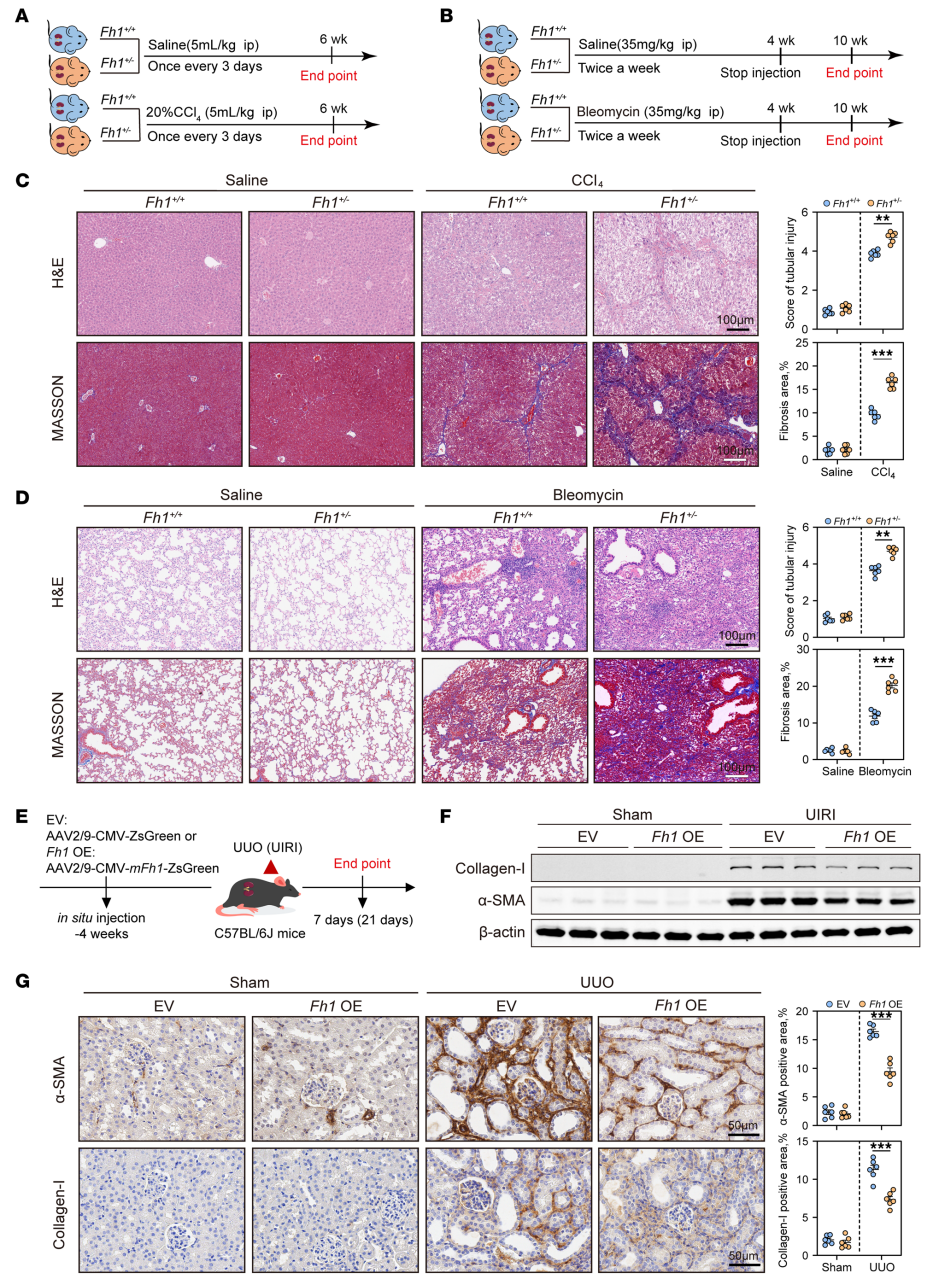
*Fh1^+/–^* mice show exacerbated liver and lung fibrosis, whereas AAV2/9-mediated FH OE alleviates renal fibrosis. (**A** and **B**) Experimental design for CCl_4_-induced liver fibrosis and bleomycin-induced pulmonary fibrosis models in *Fh1^+/+^* and *Fh1^+/–^* mice. (**C**) Representative H&E and Masson’s trichrome staining of liver sections from *Fh1*^+/+^ and *Fh1^+/–^* mice treated with vehicle or CCl_4_ (*n* = 6). Scale bar, 100 μm. (**D**) Representative H&E and Masson’s trichrome staining of lung sections from *Fh1*^+/+^ and *Fh1^+/–^* mice treated with vehicle or bleomycin (*n* = 6). Scale bar, 100 μm. (**E**) Experimental design of renal in situ delivery of AAV2/9 encoding *Fh1*. (**F**) Western blot analysis of α-SMA and collagen I in kidneys from EV and *Fh1*-OE mice after sham operation or UIRI (*n* = 6). (**G**) Representative IHC staining for α-SMA and collagen I in kidneys from EV and *Fh1*-OE mice subjected to sham operation or UUO (*n* = 6). Scale bar, 50 μm. Data are shown as mean ± SEM. Statistical analysis was performed using 2-way ANOVA followed by Tukey’s multiple comparisons test (**C**, **D**, and **G**). **P < 0.01, and ***P < 0.001.

Next, we investigated the role of FH in fibrosis in other organs. We established a liver fibrosis model by intraperitoneal injection of CCl_4_ and a pulmonary fibrosis model by bleomycin administration in *Fh1^+/–^* mice (Figure 8, A and B). In both the liver fibrosis and pulmonary fibrosis models, H&E and Masson’s trichrome staining showed that *Fh1^+/–^* mice developed more severe fibrosis than WT mice (Figure 8, C and D). IHC staining further showed increased α-SMA, collagen I, F4/80, and 2SC signals in the liver and lung of *Fh1^+/–^* mice, consistent with increased fibrosis, inflammation, and succination (Supplemental Figure 17, A and B).

### Renal in situ delivery of AAV2/9 encoding Fh1 alleviates renal fibrosis.

To investigate the therapeutic potential of FH, we induced FH OE by renal in situ delivery of recombinant AAV2/9 vectors encoding *Fh1* (Figure 8E and Supplemental Figure 18, A–C). PAS and Masson’s trichrome staining showed that, compared with WT mice, *Fh1*-OE mice exhibited reduced renal injury and fibrosis following UUO or UIRI (Supplemental Figure 18, E and G). These findings were further supported by Western blot and IHC analyses of fibrotic markers (Figure 8, F and G, and Supplemental Figure 18, D–F).

## Discussion

Persistent inflammatory responses driven by TEC damage and maladaptive repair are recognized as important contributors to the progression of renal fibrosis (41). However, the underlying mechanisms remain incompletely understood. Metabolites are widely recognized as intermediates in biosynthesis and energy metabolism, but our findings support an additional role for these molecules as regulators of inflammation. In this study, we showed that aberrant fumarate accumulation contributes to renal inflammation and fibrosis, with succination of AKAP12 at C670 suggesting a molecular link between metabolic disturbance and inflammatory activation. We found that FH abundance was closely associated with CKD severity. In addition, serum and urinary fumarate levels in patients with CKD were inversely correlated with eGFR, suggesting that fumarate may have potential as a noninvasive biomarker in CKD.

Functionally, our data showed that FH deficiency promoted renal fibrosis in association with enhanced inflammatory responses in renal TECs. Recent studies have suggested that fumarate can accumulate as a result of metabolic adaptation and exert functions beyond metabolism, particularly in the regulation of immunity and inflammation (42). In renal epithelial cells, intracellular FH downregulation has been reported to result in fumarate accumulation, which subsequently drives mitochondrial network remodeling and mitochondrial-derived vesicle formation, ultimately leading to mtDNA release and inflammatory activation (22). In addition, FH has been implicated in regulating macrophage inflammatory and interferon responses. FH downregulation in macrophages inhibits mitochondrial respiration, increases mitochondrial membrane potential, and enhances interferon-β production (28). In our study, RNA-seq analysis showed that FH deficiency in renal TECs during fibrosis was primarily associated with activation of inflammatory signaling pathways. Thus, our findings highlight a role for FH deficiency in TEC-associated inflammatory responses during renal fibrosis. These results indicate that FH may have diverse roles in regulating inflammation across different cell types under distinct pathological conditions.

Mechanistically, fumarate acts as an electrophile and can undergo Michael addition with nucleophilic cysteine residues on proteins, resulting in succination (43). To identify candidate fumarate-sensitive cysteine sites, we used a DBIA probe in a competitive cysteine-labeling strategy coupled with LC-MS/MS. AKAP12 C670 emerged as a strong candidate succination site, and immunoprecipitation analyses further supported increased succination of AKAP12 in the fibrotic cell model, which was attenuated by the C670S mutation. AKAP12 is a multifunctional scaffolding protein that binds PKCζ and restrains its activation (36). Recent studies have also implicated AKAP12 in inflammatory regulation (44). However, its role in renal fibrosis remains incompletely understood. Our data support that, in renal fibrosis, succination of AKAP12 weakens its interaction with PKCζ, thereby reducing AKAP12-mediated restraint of PKCζ activation and promoting PKCζ/NF-κB and PKCζ/β-catenin signaling. Consistently, mutation of AKAP12 C670 from cysteine to serine attenuated TGF-β1–induced activation of these pathways and reduced fibrotic responses. Together, these findings suggest that FH deficiency in renal TECs leads to fumarate accumulation, which promotes succination of AKAP12 at C670 and thereby enhances renal inflammation and fibrosis.

A limitation of the present study is that prior work has shown that increased fumarate levels in renal TECs can promote mtDNA release and thereby amplify inflammatory responses (22), whereas our findings support an additional mechanism centered on AKAP12 succination. Thus, the relative contribution of mtDNA release to the amplification of inflammatory responses during renal fibrosis remains to be defined. In addition, although our data suggest that FH contributes to liver and pulmonary fibrosis, whether the underlying mechanisms in these organs are the same as those identified in the kidney remains unclear. A further limitation is that, in the examined upstream region of the human *FH* locus, we did not identify corresponding predicted KLF9-associated motifs matching those observed upstream of mouse *Fh1*. While our data support a functional role for KLF9 in repressing *Fh1* expression in murine renal TECs under injury conditions, they do not define the cis-regulatory mechanism governing FH downregulation in human kidneys. Given that species-specific divergence of transcription factor occupancy and *cis*-regulatory architecture is a common feature of gene regulation (45, 46), we restrict our mechanistic conclusion to the murine system and consider the relevance of KLF9-dependent FH regulation in human kidney disease to be an important question for future investigation.

In conclusion, our results support a role for fumarate accumulation as an important mediator of renal inflammation and fibrosis and show that fumarate levels were closely associated with the severity of renal dysfunction in CKD. In the murine system, FH downregulation during renal fibrosis was associated with KLF9-mediated repression of *Fh1*. FH deficiency in renal TECs led to cytoplasmic fumarate accumulation, which promoted succination of AKAP12 at C670 and weakened its interaction with PKCζ. This reduced AKAP12-mediated restraint of PKCζ activation and enhanced PKCζ/NF-κB and PKCζ/β-catenin signaling, thereby exacerbating renal inflammation and fibrosis. AAV2/9-mediated FH OE markedly attenuated fibrosis progression in preclinical models. Together, these findings support an important role for aberrant fumarate accumulation in renal fibrosis and suggest that targeting fumarate accumulation may represent a therapeutic strategy in CKD.

## Methods

### Sex as a biological variable.

In clinical studies involving human participants, data from male and female patients with CKD were pooled for analysis. Sex was not included as a prespecified biological variable because the sample size was not sufficient to support adequately powered sex-stratified analyses. In the animal studies, male mice were used to reduce variability related to known sex differences in CKD susceptibility (47, 48) and to maintain consistency across experimental models. Potential sex-specific effects were not assessed in the present study and should be examined in future work.

### Human clinical samples.

Human kidney tissue sections (3–4 μm), serum, and urine samples were obtained from The First Affiliated Hospital of Anhui Medical University (Hefei, China). Tumor-free renal tissues from patients undergoing nephrectomy for renal cell carcinoma were used as control kidney tissues (*n* = 7; 5 females and 2 males), and renal tissues from patients with CKD were analyzed in parallel (*n* = 23; 8 females and 15 males). Serum and urine samples from individuals undergoing routine health examinations were used as controls (*n* = 7; 3 females and 4 males) for comparison with serum and urine from patients with CKD (*n* = 23; 8 females and 15 males). Detailed clinical information for the patients with CKD is provided in Supplemental Table 1.

### Animal studies.

Male C57BL/6J mice (6–8 weeks old, 20–22 g) were used in this study. WT and Fh1 knockout mice were generated by GemPharmatech. Fh1 conditional knock-in (cKI) mice and Akap12 C670S mutant mice were generated by Cyagen. For UUO, the left ureter was ligated, and mice were analyzed 7 days later. For UIRI, the left renal pedicle was clamped with a noninvasive microaneurysm clip for 45 minutes, followed by reperfusion, and mice were analyzed 21 days later. Liver fibrosis was induced by intraperitoneal injection of 20% CCl_4_ (5 mL/kg) every 3 days for 6 weeks. Pulmonary fibrosis was induced by intraperitoneal injection of bleomycin (35 mg/kg) twice weekly for 4 weeks, followed by a 6-week recovery period.

### Cell culture, transfection, and treatments.

mTECs were provided by Huiyao Lan (The Chinese University of Hong Kong, Hong Kong, China) and maintained as previously described (49). Cells were cultured in DMEM/F12 medium (HyClone) supplemented with 5% fetal bovine serum (Biochannel) at 37°C in a humidified incubator with 5% CO_2_. For gain- and loss-of-function experiments, cells were transfected with plasmids or siRNAs, together with the corresponding control vectors or control siRNAs (Hanbio Tech), using jetPRIME (Polyplus-transfection) according to the manufacturer’s instructions. For TGF-β1 stimulation, cells were serum-starved overnight in DMEM/F12 containing 0.5% fetal bovine serum and then treated with TGF-β1 (10 ng/mL) for 24 hours. For the H/R model, cells were subjected to 3 cycles of H/R, each consisting of 9 hours of hypoxia followed by 3 hours of reoxygenation. For FH-IN-1 treatment, cells were serum-starved overnight in DMEM/F12 containing 0.5% fetal bovine serum, pretreated with FH-IN-1 (10 μM) for 3 hours, and cotreated with TGF-β1 (10 ng/mL) for 24 hours. siRNA sequences are provided in Supplemental Table 2.

### Statistics.

Statistical analyses were performed using GraphPad Prism 9.0 (GraphPad Software). Data are presented as mean ± SEM. Comparisons between 2 groups were performed using 2-tailed unpaired Student’s *t* tests. Comparisons among multiple groups were performed using 1-way or 2-way ANOVA followed by Tukey’s multiple comparisons test, as appropriate and as specified in the figure legends. Correlations were assessed using Spearman’s correlation analysis. A *P* value less than 0.05 was considered statistically significant.

### Study approval.

Human studies were performed after written informed consent was obtained from all participants. All procedures involving human participants were conducted in accordance with the principles of the Declaration of Helsinki and were approved by the Clinical Research Ethics Committee of The First Affiliated Hospital of Anhui Medical University (approval no. PJ20241040). All animal experiments were performed in accordance with the National Institutes of Health *Guide for the Care and Use of Laboratory Animals* (National Academies Press, 2011) and were approved by the Laboratory Animal Ethics Committee of Anhui Medical University (approval no. LLSC20242194).

### Data availability.

Raw RNA-sequencing data have been deposited in the GEO database (https://www.ncbi.nlm.nih.gov/geo). The accession number is GSE293941. The mass spectrometry proteomics data have been deposited to the ProteomeXchange Consortium (https://proteomecentral.proteomexchange.org) via the iProX partner repository with the dataset identifier PXD063006. Values for all data points found in graphs are in the Supporting Data Values file. Additional details on methods can be found in the Supplemental Methods.

## Author contributions

WW, XMM, and SS conceived and designed the study. SS, XYY, YHD, and JMY performed most of the experiments. SS, XYY, XYL, JTY, and XYS performed the cell experiments. SS, YHD, JW, ZYS, ZQC, and YLZ performed the animal experiments. JMY, ZYG, and DXL collected the clinical samples and performed the related analyses. SSX and RH performed the GEO dataset analysis and molecular docking. SS, XYY, and YHD analyzed the data and drafted the manuscript. XXX, JJ, JGW, and HW critically revised the manuscript and polished the language. WW supervised the study, interpreted the data, critically revised the manuscript, and approved the final version.

## Conflict of interest

The authors have declared that no conflict of interest exists.

## Funding support

- National Natural Science Foundation of China (82270737 and 82470732, WW).
- Outstanding Youth Project of the Natural Science Foundation for Universities in Anhui Province (2023AH020047, WW).
- Key Project for Cultivating Outstanding Young Teachers in Universities of Anhui Province (YQZD2023022, WW).

## Supplementary Material

Supplemental data

Unedited blot and gel images

Supporting data values

## Version 1 06/30/2026

In-Press Preview

## Version 2 07/15/2026

Electronic publication
