# Supplementary material for: Fumarate-induced succination of A-kinase anchor protein 12 exacerbates renal inflammation and fibrosis
Source: J Clin Invest. 2026 Jun 30;136(14):e200755. doi: 10.1172/JCI200755 (PMC13367978; doi:10.1172/JCI200755)
Supplement: Supplemental data [file jci-136-200755-s074.pdf]

Supplemental Materials

Fumarate-induced succination of A-kinase anchor protein 12 exacerbates renal inflammation and fibrosis

Authors: Shuai Sun[1,2,3]†, Xu-yang Yan[2,3]†, Yu-hang Dong[4,5]†, Jian-min You[2,3]†, Zhen-yu Guo[2,3], Dong-xue Lv[4], Shuai-shuai Xie[4], Rui Hou[4], Xiang-yu Li[4], Ju-tao Yu[4], Xiao-yu Shen[4], Jie Wei[4], Zhen-yu Song[2,3], Zi-qi Chen[2,3], Yun-long Zhu[2,3], Xing-xin Xu[6], Juan Jin[4], Jia-gen Wen[4], Hao Wang[7], Xiao-ming Meng[4]*, Wei Wang[2,3]*

Inventory for Supplemental Materials

I. Supplemental Methods

II. Supplemental Figures

III. Supplemental Tables

Supplemental Methods

Study design

This study examined the role of FH in renal fibrosis and the underlying molecular mechanisms. Human kidney biopsy specimens, serum, and urine samples were obtained from patients with CKD at the First Affiliated Hospital of Anhui Medical University (Hefei, China). Tumor-free renal tissue from patients undergoing nephrectomy for renal cell carcinoma was used as the control for renal FH expression analysis, and serum and urine from individuals undergoing routine health examinations served as controls for fumarate measurements. Written informed consent was obtained from all participants. For animal studies, we generated *Fh1* knockout mice, proximal tubule–specific *Fh1* conditional knock-in mice, and $Akap12^{CS/CS}$ mutant mice. Littermate mice were used for randomization and as controls. Sample size was determined on the basis of ANOVA and prior experience with similar models; 6 mice were included per group unless otherwise indicated in the figure legends. Renal fibrosis was induced by UUO for 7 days or UIRI for 21 days. Carbon tetrachloride–induced liver fibrosis and bleomycin-induced pulmonary fibrosis models were also established. To assess the therapeutic potential of FH, AAV2/9-mediated FH overexpression was performed *in vivo*. In cell studies, FH knockdown and overexpression experiments in tubular epithelial cells were performed in 3 independent experiments, as indicated in the figure legends. Statistical methods for each experiment are specified in the corresponding figure legends.

Reagents and materials

The following antibodies were used in this study: anti-FH (Abcam, ab233394), anti-α-SMA (Abcam, ab124964), anti-Collagen-I (Proteintech, 66761-1-Ig), anti-phospho-NF-κB p65 (Cell Signaling Technology, #3033), anti-NF-κB p65 (Cell Signaling Technology, #8242), anti-F4/80 (Cell Signaling Technology, #70076), anti- phospho-PKCζ (Cell Signaling Technology, #2060), anti-PKCζ (Proteintech, 26899-1-AP), anti-phospho-IκB (Cell Signaling Technology, #2859), anti-IκB (Cell Signaling Technology, #4812), anti-phospho-β-catenin (Cell Signaling Technology, #9561), anti-β-catenin (Cell Signaling Technology, #8480), anti-phospho-GSK-3β (Cell Signaling Technology, #5558), anti-GSK-3β (Cell Signaling Technology, #12456), HRP-conjugated goat anti-rabbit/mouse secondary antibody (Cell Signaling Technology, #7074/7076), anti-KLF9 (GeneTex, GTX129316), anti-AKAP12 (Proteintech, 25199-1-AP), anti-DYKDDDDK tag (Proteintech, 20543-1-AP), anti-2SC (Biosynth, CRB2005017), anti-β-actin (Abclonal, AC026), fluorescein-labeled LTL (Vector laboratories, #FL-1321) and IRDye 800CW goat anti-rabbit/mouse secondary antibody (LI-COR Biosciences; #926-32211/926-32210). The following reagents were used: TGF-β1 (MedChemExpress, HY-P7117 and HY-P70543), fumarate hydratase-IN-1 (MedChemExpress, HY-100004), monomethyl fumarate (Sigma-Aldrich, 651419), carbon tetrachloride (Macklin, C805325), and bleomycin sulfate (Solarbio, IB0871).

Generation and validation of *Fh1* knockout mice

Fh1 knockout mice were generated by GemPharmatech Co., Ltd (Nanjing, China) using CRISPR/Cas9-mediated genome editing on a C57BL/6J background. Two single-guide RNAs (sgRNAs) targeting exons 3 to 9 of the *Fh1*-201 transcript (sgRNA1: 5'-TGTG CTCCCACACTCACGCT-3', PAM: CGG; sgRNA2: 5'-CCTGTAAGACCCTCCCTCGT-3', PAM: TGG) were designed to induce a large genomic deletion. Cas9 mRNA and the sgRNAs were co-microinjected into fertilized mouse zygotes. Founder mice were identified through PCR-based genotyping and were subsequently bred to establish stable lines. Given that homozygous deletion of *Fh1* results in embryonic lethality, heterozygous $Fh1^{+/-}$ mice were utilized for all experiments. The primer sequences employed for PCR genotyping are provided in Supplemental Figure 3A.

Generation and validation of *Fh1* conditional knock-in mice

Fh1 conditional knock-in (cKI) mice were generated by Cyagen Company (Suzhou, China) utilizing CRISPR/Cas9-mediated genome editing on a C57BL/6J background. The targeting vector comprised a CAG promoter, a loxP-flanked STOP cassette, the Kozak-Mouse *Fh1* coding sequence (CDS) tagged with 3×FLAG, a woodchuck hepatitis virus post-transcriptional regulatory element (WPRE), and a polyA signal. This complete construct was integrated into the Gt(ROSA)26Sor locus between exon 1 and exon 2. A sgRNA targeting the ROSA26 locus (5'-CTCCAGTCTTTCTAGAAGAT-3', PAM: GGG)

was designed. Cas9 protein, sgRNA, and the targeting vector were co-microinjected into fertilized mouse zygotes. Founder mice were identified through PCR-based genotyping and subsequently bred to establish stable lines. The Cre-mediated excision of the STOP cassette facilitated the conditional expression of *Fh1*. The primer sequences used for PCR genotyping are provided in Supplemental Figure 5A.

Generation and validation of *Akap12* C670S mutation mice

The *Akap12* C670S mutation mice were generated by Cyagen Company (Suzhou, China) utilizing CRISPR/Cas9-mediated genome editing on a C57BL/6J background. Cas9 protein, along with one gRNA (5’-TGGGAGGCGTTGATTTGTGT-3’, PAM: CGG), and a donor oligonucleotide containing the C670S mutation (TGT to AGC) were co-injected into fertilized eggs. The embryos were subsequently transferred to recipient female mice to obtain F0 mice. The genotype of the point mutation mice was confirmed by PCR using a pair of primers (F1: 5’-AAGACCTTCTGAGAGCGACAAG-3’, R1: 5’-TTCGGCTCTCT CTTCCAGTTTTG-3’) and sequencing. F0 founder mice were then crossed with C57BL/6J mice to produce heterozygous F1 offspring.

Construction and intrarenal delivery of recombinant AAV2/9 vectors

A chimeric adeno-associated virus serotype 2/9 (AAV2/9) system was utilized for *in vivo* gene delivery. The vectors employed AAV2 inverted terminal repeats packaged within an

AAV9 capsid. To facilitate the overexpression of *Fh1*, an AAV2/9 vector encoding mouse *Fh1*, along with ZsGreen under the control of a CMV promoter, was constructed. Control mice received an AAV2/9 vector expressing ZsGreen alone, which lacked the *Fh1* coding sequence. All viral vectors were produced and purified to high titers by Hanbio Tech (Shanghai, China). For *in vivo* delivery, mice were anesthetized, and the left kidney was surgically exposed. AAV2/9 viral solution (100 μL per mouse, 1.8×10^{12} vg/mL; total dose of 1.8×10^{11} vg) was slowly injected directly into the renal parenchyma using a microsyringe under visual guidance. To ensure broad and uniform distribution within the kidney, injections were performed at no fewer than five evenly distributed sites across the cortical regions of the unilateral kidney. Recombinant AAV2/9 vectors encoding *Fh1* were administered four weeks prior to modeling in the mice.

Isolation of primary human renal tubular epithelial cells

Normal renal cortical tissue was collected from tumor-adjacent, nonneoplastic regions. The renal cortex was finely minced and suspended in low-glucose culture medium, followed by centrifugation at 1500 rpm for 5 minutes. Tissue pellets were then digested with type II collagenase with gentle pipetting to facilitate dissociation and incubated in a rotary mixer at 70 rpm and 37°C for 20 minutes. The digested suspension was sequentially filtered through 70-μm and 40-μm cell strainers. Complete medium was added to terminate digestion. Cells were collected by centrifugation at 400 × g for 10 minutes, resuspended in fresh medium,

and seeded into culture vessels. Cultures were left undisturbed for the first 12 hours. The medium was first changed after 48 hours and then replaced daily thereafter.

Western blot

Kidney tissues and cultured cells were lysed in radioimmunoprecipitation assay (RIPA) buffer supplemented with protease inhibitors (Solarbio, Beijing, China). Equal amounts of protein were separated by SDS-PAGE and transferred to nitrocellulose membranes (Pall Corporation, Port Washington, New York, USA). Membranes were blocked with 5% nonfat milk and incubated overnight at 4°C with the indicated primary antibodies. After washing, membranes were incubated with IRDye-conjugated secondary antibodies (1:10,000; LI-COR Biosciences) for 1 hour at room temperature. Protein bands were detected using an Odyssey Imaging System (LI-COR Biosciences) and quantified using ImageJ (National Institutes of Health, Bethesda, MD, USA).

RNA isolation and qRT-PCR

Total RNA was extracted using TRIzol reagent (Invitrogen, Thermo Fisher Scientific), and RNA quality was assessed before analysis. cDNA was synthesized from 1 μg of total RNA using the HiScript cDNA synthesis kit with gDNA Eraser (Vazyme Biotech, Nanjing, China). qRT-PCR was performed using Universal SYBR qPCR master mix (Vazyme

Biotech, Nanjing, China). Gene expression was normalized to *Actb*. Primer sequences are provided in Supplemental Table 3.

Histology, immunohistochemistry, and tubular injury scoring

Tissues were fixed in 4% paraformaldehyde, embedded in paraffin, and sectioned at 4 μm. H&E, PAS, Masson's trichrome, and Sirius red staining were performed according to the manufacturers' instructions using commercially available kits (Solarbio: H&E, G1120; PAS, G1281; Modified Masson's Trichrome, G1346; Modified Sirius Red, G1472). For IHC, tissue sections were incubated with the indicated primary antibodies, including anti-FH, anti-alpha-SMA, anti-collagen I, and anti-F4/80, followed by HRP-conjugated secondary antibodies. Staining was visualized with DAB substrate, and sections were counterstained with hematoxylin. Images were acquired using a light microscope (Leica Microsystems, Wetzlar, Germany). Tubular injury was assessed on H&E- and PAS-stained kidney sections using a semiquantitative scoring system based on the percentage of injured tubules: 0, no injury; 1, 1%–10%; 2, 11%–25%; 3, 26%–50%; 4, 51%–75%; 5, 76%–90%; and 6, >90%. Tubular injury was defined by tubular dilation, protein cast formation, loss of brush borders, and tubular epithelial cell sloughing. Scoring was performed independently by 3 experienced renal pathologists blinded to the experimental groups. Scores were recorded to 1 decimal place, and the final score for each sample was calculated as the mean of the 3 observers' scores.

Immunofluorescence staining

Tissue sections were deparaffinized, rehydrated, and subjected to heat-mediated antigen retrieval in Tris-EDTA buffer (pH 9.0; Servicebio, Wuhan, China) at 95°C for 20 minutes. After blocking with 5% BSA (Sigma-Aldrich), sections were incubated overnight at 4°C with the indicated primary antibodies. Alexa Fluor 488– or Alexa Fluor 594–conjugated secondary antibodies (Servicebio, Wuhan, China) were then applied for 1 hour at room temperature in the dark. Nuclei were counterstained with DAPI (Servicebio, Wuhan, China), and sections were mounted with antifade mounting medium. Images were acquired using a fluorescence microscope (Carl Zeiss, Oberkochen, Germany).

Cell immunofluorescence staining

Cultured cells were fixed with 4% paraformaldehyde for 15 minutes at room temperature, permeabilized with 0.1% Triton X-100 for 10 minutes, and blocked with 5% BSA for 1 hour at room temperature. Cells were then incubated overnight at 4°C with the indicated primary antibodies. The following day, fluorophore-conjugated secondary antibodies were applied, and nuclei were counterstained with DAPI. Images were acquired using a fluorescence microscope (Carl Zeiss, Oberkochen, Germany).

ChIP assay

ChIP assays were performed using the SimpleChIP Enzymatic Chromatin IP Kit (Cell Signaling Technology) according to the manufacturer's instructions. KLF9-associated chromatin was immunoprecipitated using an anti-KLF9 antibody, with normal rabbit IgG used as a negative control. Enriched DNA fragments were analyzed by qPCR using mouse *Fh1* primers to assess relative enrichment at the indicated genomic region. The mouse *Fh1* primer sequences were as follows: forward, 5′-ACATTTCGGGCAGAAGATACACT-3′; reverse, 5′-AGTAGTTGGTGAAAGGTAGAACCA-3′.

Measurement of fumarate content

Fumarate levels were measured using a Fumarate Assay Kit (Sigma-Aldrich, MAK060) according to the manufacturer's instructions.

RNA-seq

RNA-seq library preparation, high-throughput sequencing, and bioinformatic analysis were performed by BGI Genomics (Shenzhen, China). Total RNA was extracted from TECs using TRIzol reagent and treated with DNase I. mRNA was enriched using Oligo(dT) magnetic beads, fragmented, and reverse transcribed into cDNA using random primers. The resulting cDNA was amplified, end repaired, ligated, and PCR amplified. PCR products were recovered and circularized to generate single-stranded circular DNA libraries. After removal of uncircularized linear DNA, qualified libraries were amplified by rolling circle

replication to generate DNA nanoballs, which were then sequenced on the BGISEQ platform.

Competitive DBIA labeling and LC-MS/MS analysis

Cell lysates were collected and disrupted by sonication, followed by centrifugation at 20,000 × g for 30 minutes at 4°C. The supernatants were adjusted to a final protein concentration of 2 mg/mL. Samples were then incubated with dimethyl sulfoxide or 1 mM monomethyl fumarate at 25°C for 16 hours. Proteins were denatured with urea and reduced with dithiothreitol. Samples were then incubated with DBIA for 1 hour at room temperature in the dark. Carboxylated magnetic beads were used for protein precipitation, and bead-bound proteins were subjected to overnight enzymatic digestion. The resulting digests were enriched using streptavidin beads with rotation at 25°C for 4 hours. After enrichment, samples were washed 3 times with PBS containing 1 M NaCl to remove nonspecifically bound peptides. Peptides were then eluted, combined, dried, and resuspended in 0.1% formic acid before LC-MS/MS analysis. LC-MS/MS was performed using a Vanquish Neo liquid chromatography system coupled to an Orbitrap Exploris 480 mass spectrometer (Thermo Fisher Scientific) under low-pH chromatographic conditions.

Molecular docking

The fumarate structure was obtained from the PubChem database (CID 444972; CAS 110-17-8). The AKAP12 protein sequence was retrieved from the UniProt database (accession no. Q9WTQ5). Because no experimentally resolved structure of AKAP12 was available in the Protein Data Bank (PDB), the AlphaFold2-predicted 3-dimensional structure was used for docking analysis. Ligand preparation was performed using the LigPrep module in Schrödinger with the OPLS4 force field. Possible stereoisomers and protonation states were generated using the Epik module. Covalent docking was performed using the Schrödinger covalent docking module, with Cys670 designated as the reactive site and Michael addition selected as the reaction type. Docking scores were interpreted according to the Schrödinger scoring system, in which lower scores indicate stronger predicted binding.

Co-IP assay

Cells were washed 3 times with ice-cold PBS and lysed in NP-40 buffer supplemented with protease and phosphatase inhibitors (Beyotime Biotechnology). Lysates were clarified by centrifugation, and protein concentrations were determined. Equal amounts of protein were incubated with the indicated primary antibody or control IgG, followed by incubation with protein A/G agarose beads (Bio-linkedin, China). Bound proteins were eluted in SDS sample buffer, separated by SDS-PAGE, and analyzed by western blot.

Supplemental Figures

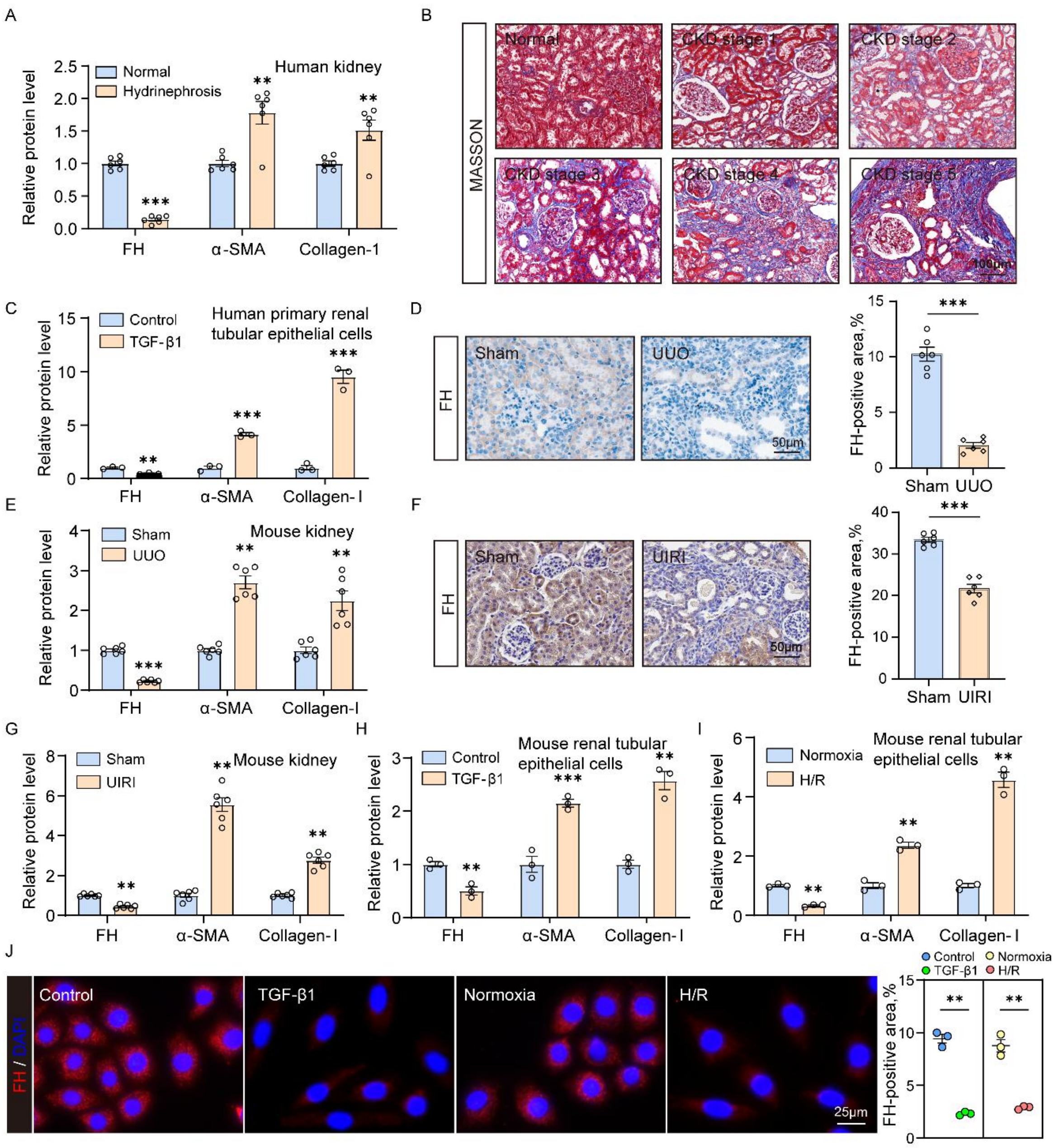

Supplemental Figure 1. FH expression is reduced in CKD kidneys and experimental models of renal fibrosis.

(**A**) Densitometric quantification of FH, α-SMA, and collagen-I protein levels from the

corresponding immunoblots using ImageJ. (**B**) Representative Masson's trichrome staining of human kidney tissues from normal controls and patients with CKD stages 1-5 (n = 30). Scale bar, 100 μm. (**C**) Densitometric quantification of FH, α-SMA, and collagen-I protein levels from the corresponding immunoblots using ImageJ. (**D** and **F**) Representative IHC staining for FH in kidneys from WT mice subjected to sham operation or UUO/UIRI (n = 6) Scale bar, 50 μm. (**E** and **G–I**) Densitometric quantification of FH, α-SMA, and collagen-I protein levels from the corresponding immunoblots using ImageJ. (**J**) Representative IF staining showing FH expression in mTECs treated with TGF-β1 or H/R (n = 3). Scale bar, 25 μm. Data are shown as mean ± SEM. Statistical analysis was performed using 2-tailed unpaired Student's *t* test (**A** and **C–J**). **P* < 0.05, ***P* < 0.01, and ****P* < 0.001.

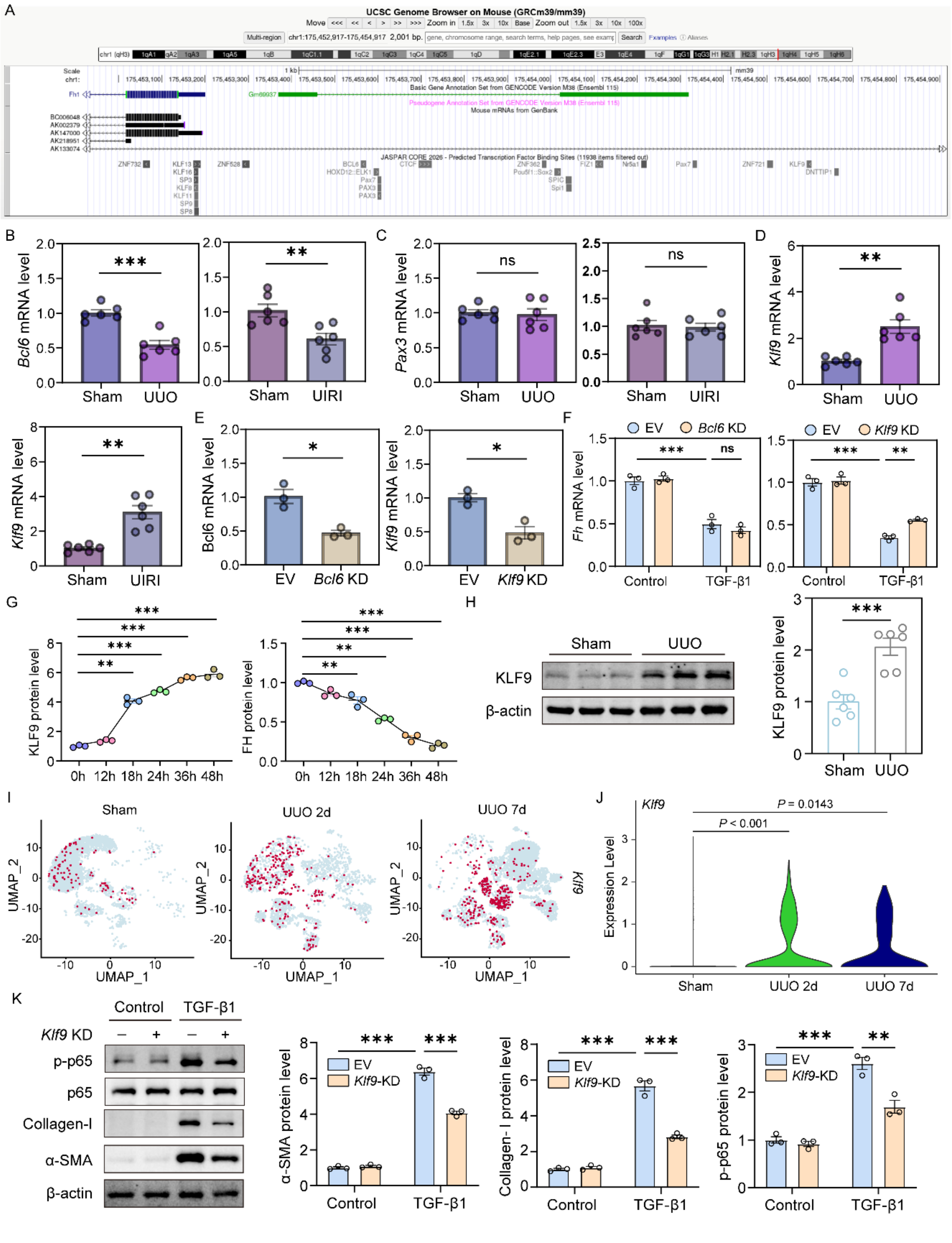

Supplemental Figure 2. KLF9 regulates FH expression during renal fibrosis.

(**A**) UCSC Genome Browser view of the upstream regulatory region of mouse *Fh1* showing

annotated transcription factor-associated motifs. (**B–D**) qRT-PCR analysis of *Bcl6*, *Pax3*, and *Klf9* mRNA expression in kidneys from mice subjected to sham operation, UUO, or UIRI (n = 6). (**E**) qRT-PCR analysis of *Bcl6* and *Klf9* knockdown efficiency. (**F**) qRT-PCR analysis of *Fh1* mRNA levels in EV and BCL6- or KLF9-knockdown cells (n = 3). (**G**) Densitometric quantification of FH and KLF9 protein levels from the corresponding immunoblots using ImageJ. (**H**) Representative immunoblot showing KLF9 protein levels in kidneys from mice subjected to sham operation or UUO (n = 6). (**I** and **J**) Feature plot and violin plot showing *Klf9* expression in renal proximal tubular epithelial cells under sham, UUO 2-day, and UUO 7-day conditions in GSE140023. (**K**) Immunoblot analysis of α-SMA, collagen-I, and p-p65 in EV and *Klf9*-knockdown cells with or without TGF-β1 stimulation (n = 3). Data are shown as mean ± SEM. Statistical analysis was performed using 2-tailed unpaired Student's *t* test (**B–E** and **H**), 1-way ANOVA followed by Tukey's multiple-comparisons test (**G**), and 2-way ANOVA followed by Tukey's multiple-comparisons test (**F** and **K**). *$P < 0.05$, **$P < 0.01$, and ***$P < 0.001$.

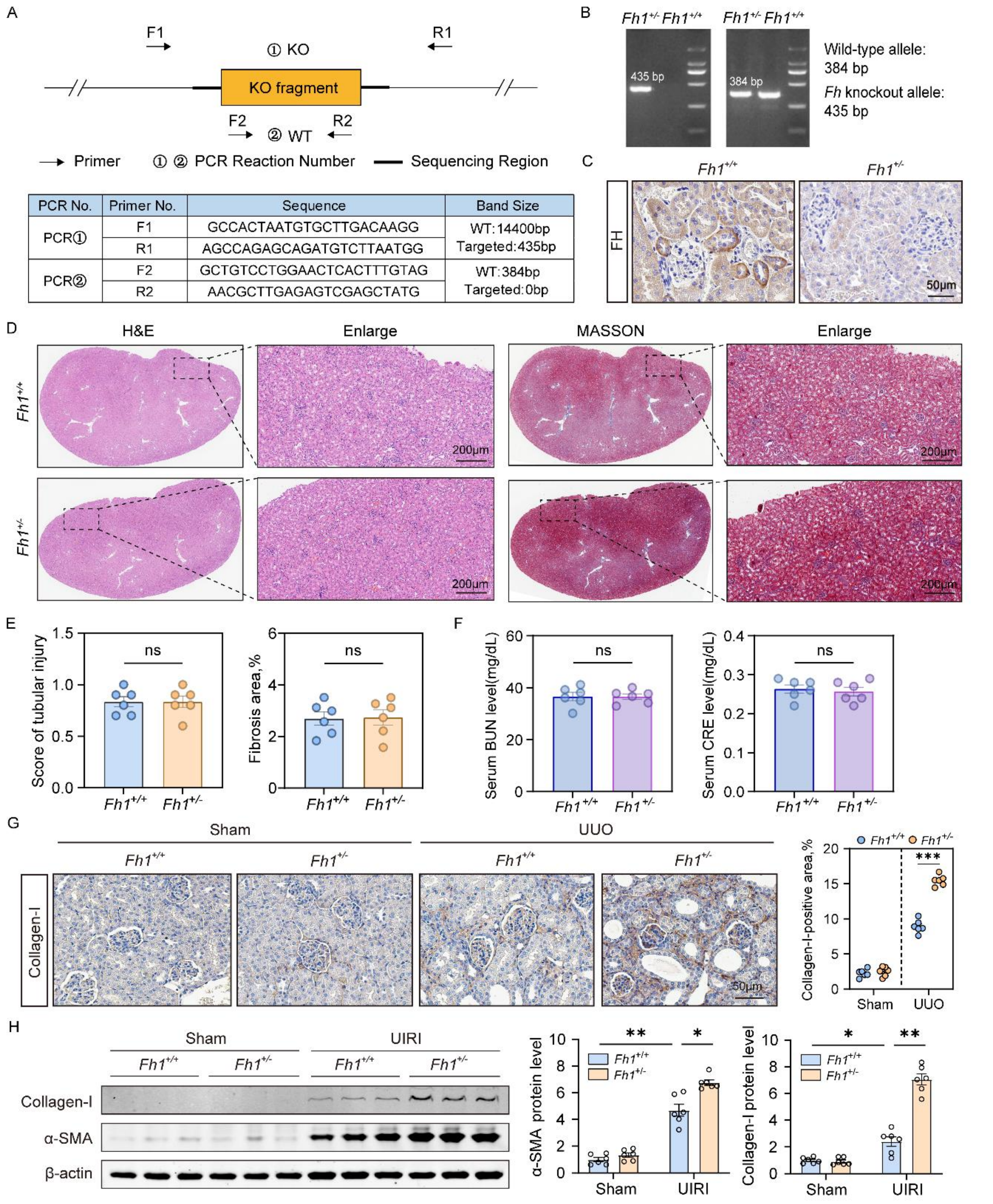

Supplemental Figure 3. *Fh1*$^{+/-}$ mice show exacerbated UUO- and UIRI-induced renal fibrosis.

(**A**) Schematic illustration of the *Fh1* knockout genotyping strategy. (**B**) PCR-based

genotyping of mice by agarose gel electrophoresis. (**C**) Representative IHC staining for FH in kidneys from *Fh1*$^{+/+}$ and *Fh1*$^{+/-}$ mice (n = 6). Scale bar, 50 µm. (**D** and **E**) Representative H&E and Masson's trichrome staining of kidneys from *Fh1*$^{+/+}$ and *Fh1*$^{+/-}$ mice (n = 6). Scale bar, 200 µm. (**F**) Serum BUN and CRE levels in *Fh1*$^{+/+}$ and *Fh1*$^{+/-}$ mice. (**G**) Representative IHC staining for collagen-I in the kidneys of *Fh1*$^{+/+}$ and *Fh1*$^{+/-}$ mice after sham operation or UUO (n = 6). Scale bar, 50 µm. (**H**) Immunoblot analysis of α-SMA and collagen-I in kidneys from *Fh1*$^{+/+}$ and *Fh1*$^{+/-}$ mice after sham operation or UIRI (n = 6). Data are shown as mean ± SEM. Statistical analysis was performed using 2-tailed unpaired Student's *t* test (**E** and **F**) and 2-way ANOVA followed by Tukey's multiple-comparisons test (**G** and **H**). *$P < 0.05$, **$P < 0.01$, and ***$P < 0.001$.

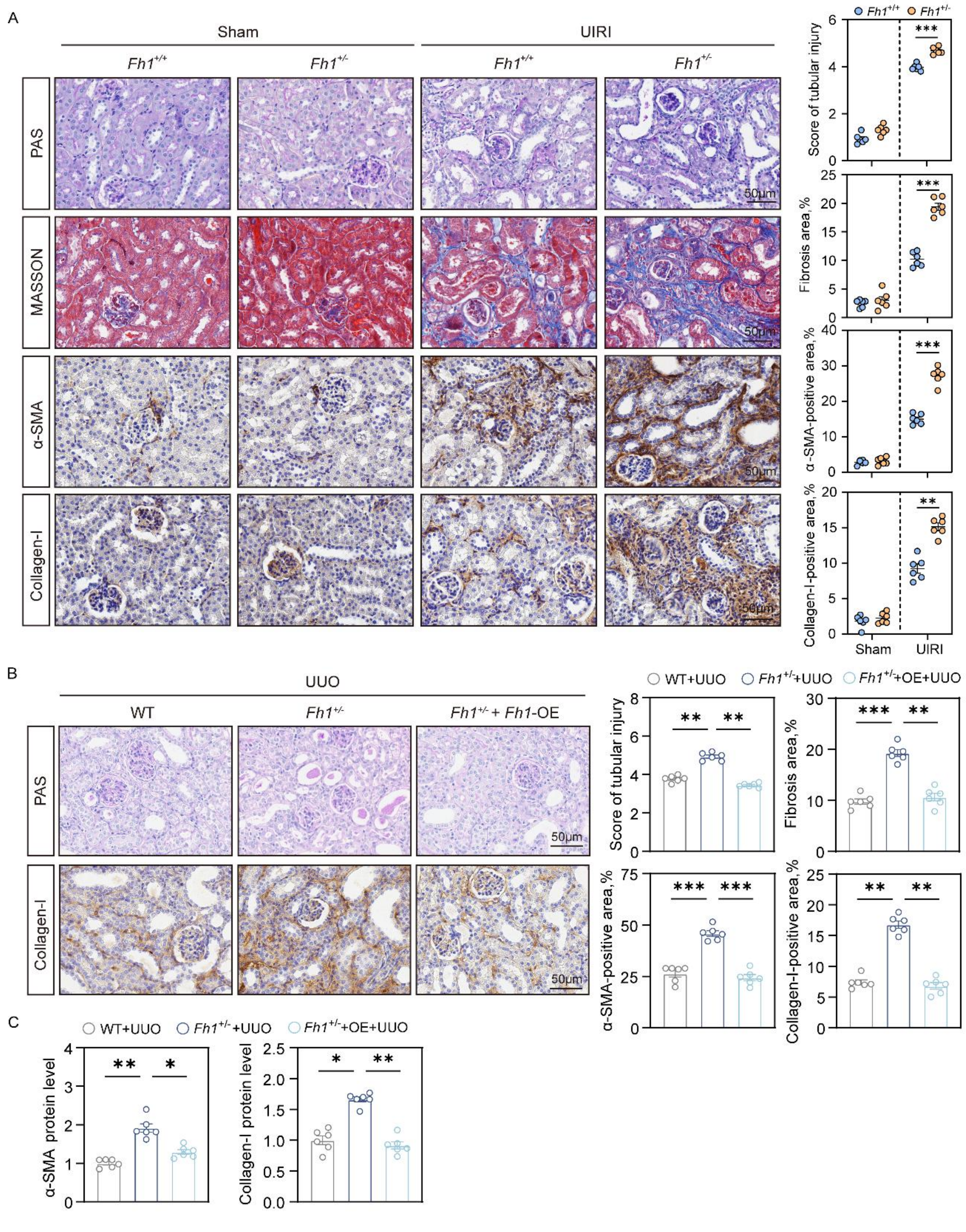

Supplemental Figure 4. Restoration of FH expression attenuates UUO-induced renal fibrosis in *Fh1*$^{+/-}$ mice.

(**A**) Representative PAS, Masson's trichrome, and IHC staining for α-SMA and collagen-I

in kidneys from *Fh1*$^{+/+}$ and *Fh1*$^{+/-}$ mice after sham operation or UIRI (n = 6). Scale bar, 50 μm. (**B**) Representative PAS and IHC staining for collagen-I in kidneys from WT mice, *Fh1*$^{+/-}$ mice, and *Fh1*$^{+/-}$ mice with FH overexpression after UUO (n = 6). Scale bar, 50 μm. (**C**) Densitometric quantification of α-SMA and collagen-I protein levels from the corresponding immunoblots using ImageJ. Data are shown as mean ± SEM. Statistical analysis was performed using 1-way ANOVA followed by Tukey's multiple-comparisons test (**B** and **C**) and 2-way ANOVA followed by Tukey's multiple-comparisons test (**A**). *$P < 0.05$, **$P < 0.01$, and ***$P < 0.001$.

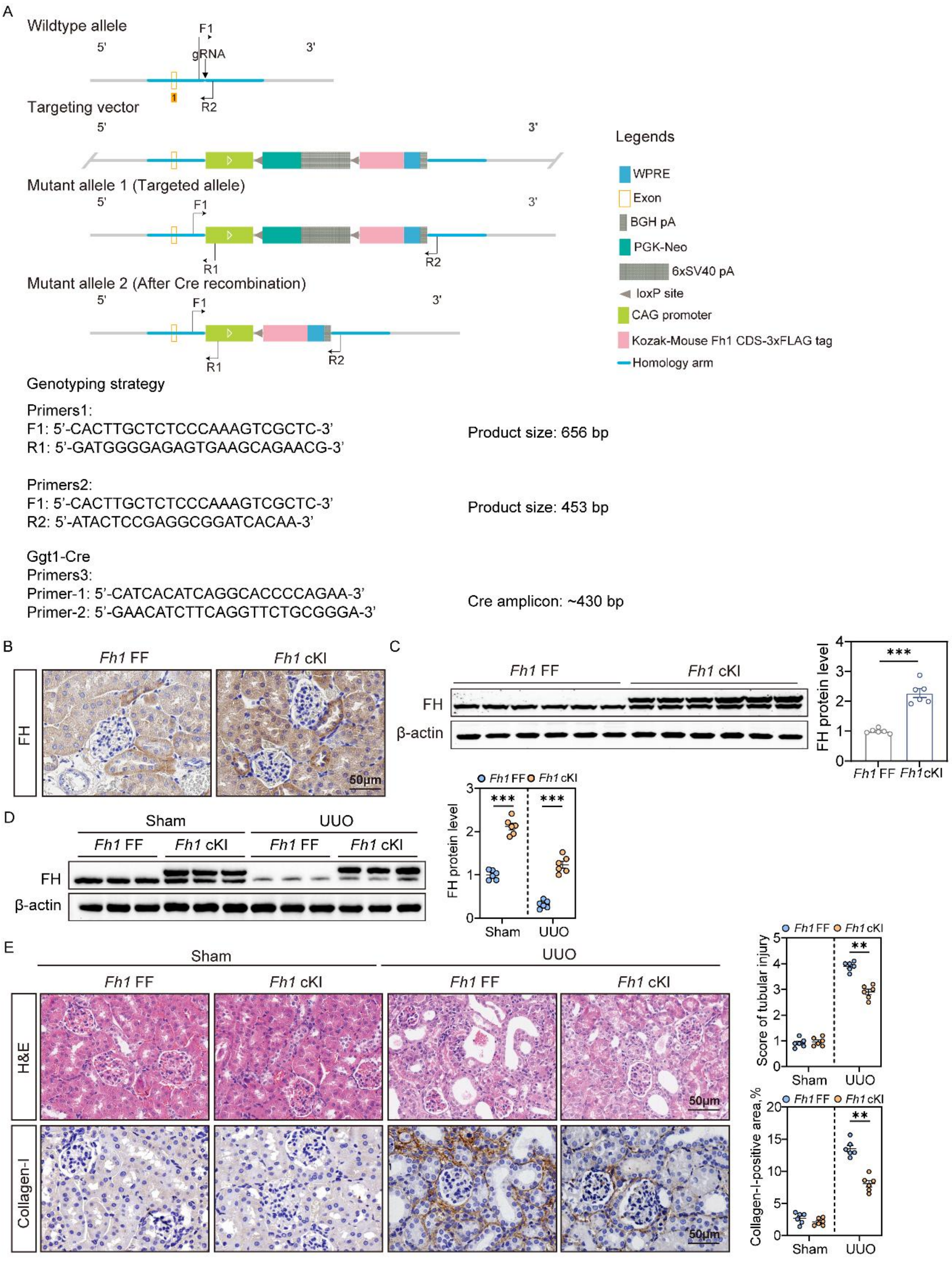

Supplemental Figure 5. Conditional knock-in of *Fh1* attenuates UUO-induced renal

fibrosis.

(**A**) Genotyping strategy for *Fh1* cKI mice. (**B**) Representative IHC staining for FH in the kidneys from *Fh1* FF and *Fh1* cKI mice (n = 6). Scale bar, 50 µm. (**C**) Immunoblot analysis of FH protein levels in kidneys from *Fh1* FF and *Fh1* cKI mice (n = 6). (**D**) Immunoblot analysis of FH protein levels in kidneys from *Fh1* FF and *Fh1* cKI mice after sham operation or UUO (n = 6). (**E**) Representative H&E and IHC staining for collagen-I in kidneys from *Fh1* FF and *Fh1* cKI mice after sham operation or UUO (n = 6). Scale bar, 50 µm. Data are shown as mean ± SEM. Statistical analysis was performed using 2-tailed unpaired Student's *t* test (**C**) and 2-way ANOVA followed by Tukey's multiple-comparisons test (**D** and **E**). *$P < 0.05$, **$P < 0.01$, and ***$P < 0.001$.

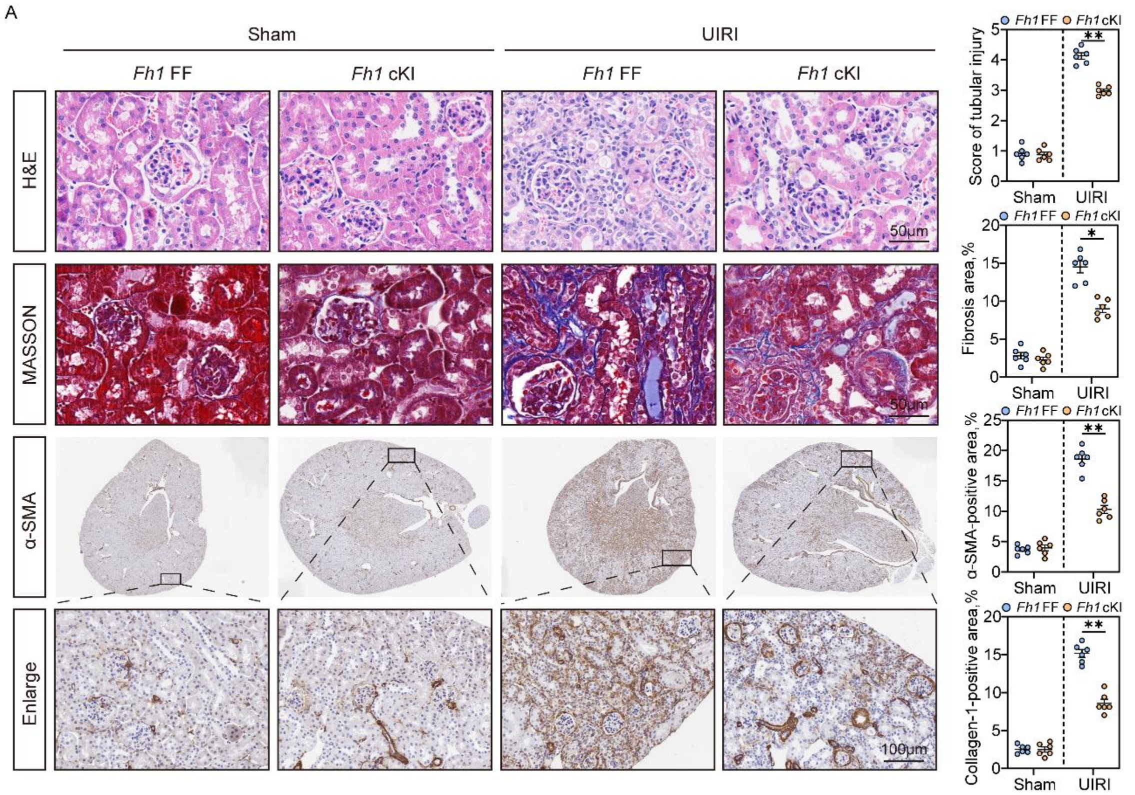

Supplemental Figure 6. Conditional knock-in of *Fh1* attenuates UIRI-induced renal fibrosis.

(**A**) Representative H&E, Masson's trichrome, and IHC staining for α-SMA in kidneys from *Fh1* FF and *Fh1* cKI mice after sham operation or UIRI (n = 6). Scale bar, 50/100 μm. Data are shown as mean ± SEM. Statistical analysis was performed using 2-way ANOVA followed by Tukey's multiple-comparisons test (**A**). **P* < 0.05, ***P* < 0.01, and ****P* < 0.001.

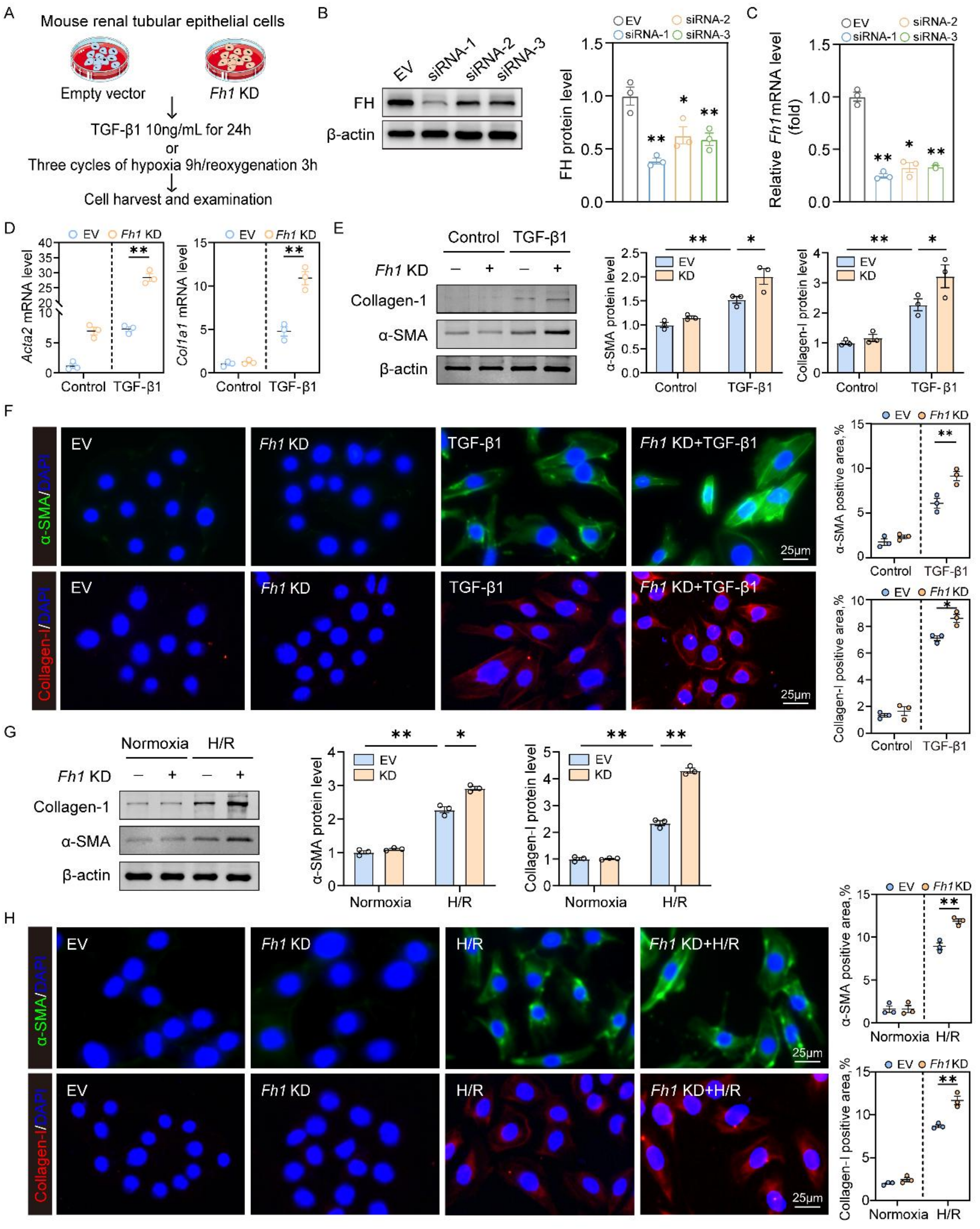

Supplemental Figure 7. FH silencing promotes fibrotic responses in TGF-β1– and H/R-treated TECs.

(**A**) Schematic overview of the experimental design. (**B** and **C**) Western blot and qRT-PCR analyses confirming FH knockdown in EV and *Fh1* KD TECs (n = 3). (**D** and **E**) qRT-PCR and immunoblot analyses of fibrotic markers in EV and *Fh1* KD TECs with or without TGF-β1 treatment (n = 3). (**F**) Representative IF staining for α-SMA and collagen-I in EV and *Fh1* KD TECs with or without TGF-β1 treatment (n = 3). Scale bar, 25 μm. (**G** and **H**) Immunoblot and IF analyses of α-SMA and collagen-I in EV and *Fh1* KD TECs with or without H/R treatment (n = 3). Scale bar, 25 μm. Data are shown as mean ± SEM. Statistical analysis was performed using 1-way ANOVA followed by Tukey's multiple-comparisons test (**B** and **C**) and 2-way ANOVA followed by Tukey's multiple-comparisons test (**D**–**H**). *$P < 0.05$, **$P < 0.01$, and ***$P < 0.001$.

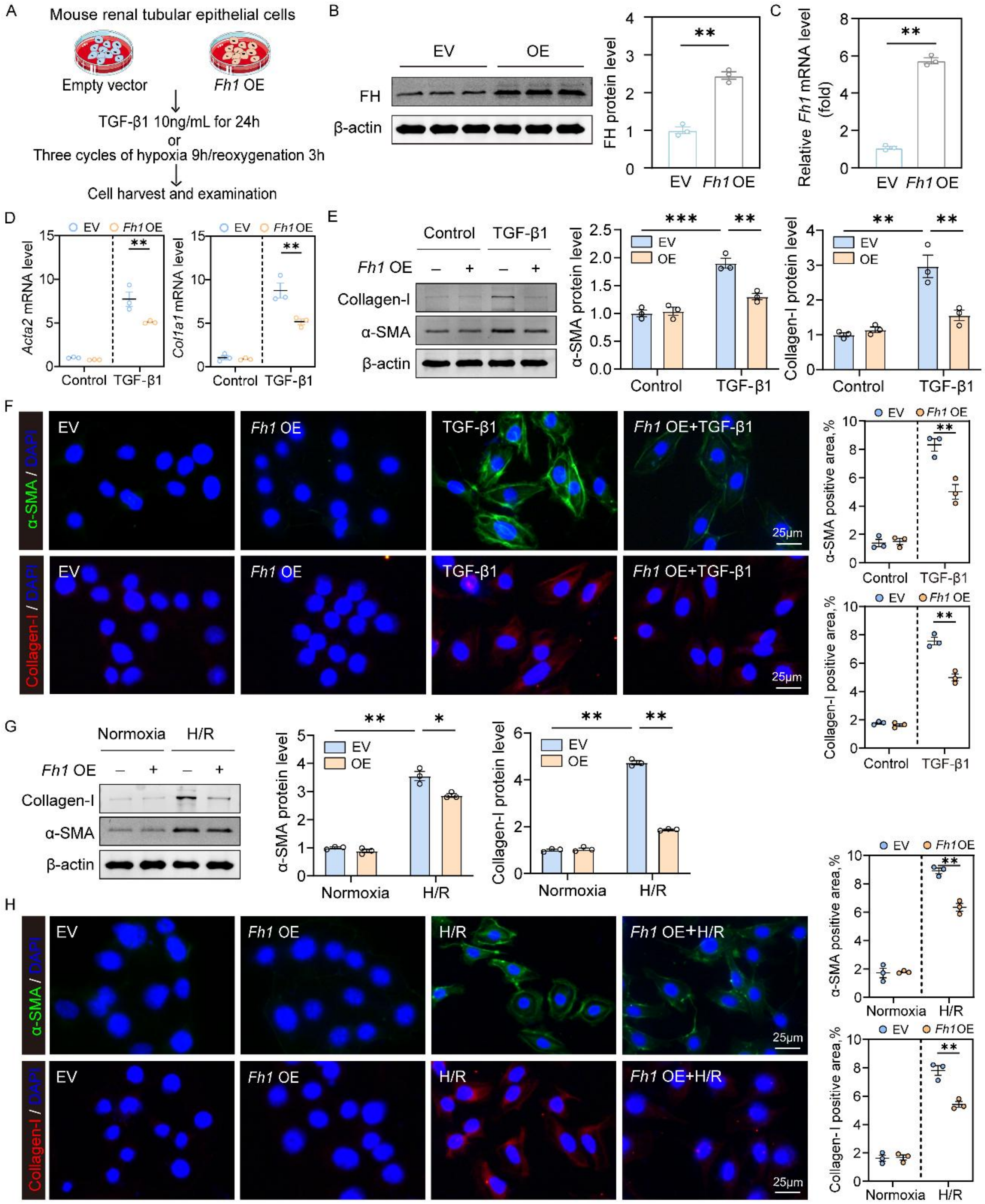

Supplemental Figure 8. FH overexpression attenuates fibrotic responses in TGF-β1– and H/R-treated TECs.

(**A**) Schematic overview of the experimental design. (**B** and **C**) Western blot and qRT-PCR

analyses confirming FH overexpression in EV and *Fh1* OE TECs (n = 3). (**D** and **E**) qRT-PCR and immunoblot analyses of fibrotic markers in EV and *Fh1* OE TECs with or without TGF-β1 treatment (n = 3). (**F**) Representative IF staining for α-SMA and collagen-I in EV and *Fh1* OE TECs with or without TGF-β1 treatment (n = 3). Scale bar, 25 μm. (**G** and **H**) Immunoblot and IF analyses of α-SMA and collagen-I in EV and *Fh1* OE TECs with or without H/R treatment (n = 3). Scale bar, 25 μm. Data are shown as mean ± SEM. Statistical analysis was performed using 2-tailed unpaired Student's *t* test (**B** and **C**) and 2-way ANOVA followed by Tukey's multiple-comparisons test (**D**–**H**). *$P < 0.05$, **$P < 0.01$, and ***$P < 0.001$

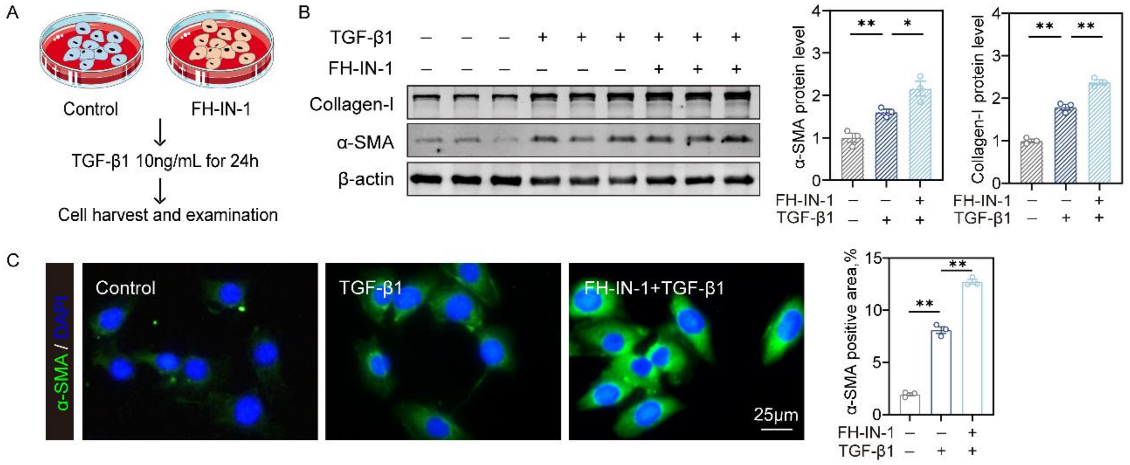

Supplemental Figure 9. Inhibition of FH exacerbates fibrotic responses in TECs.

(**A**) Schematic overview of the experimental design. (**B**) Immunoblot analysis of fibrotic markers in control and FH-IN-1–treated TECs with or without TGF-β1 stimulation (n = 3). (**C**) Representative IF staining for α-SMA in control and FH-IN-1-treated TECs with or without TGF-β1 stimulation (n = 3). Scale bar, 25 μm. Data are shown as mean ± SEM. Statistical analysis was performed using 1-way ANOVA followed by Tukey's multiple-comparisons test (**B** and **C**). *$P < 0.05$, **$P < 0.01$, and ***$P < 0.001$.

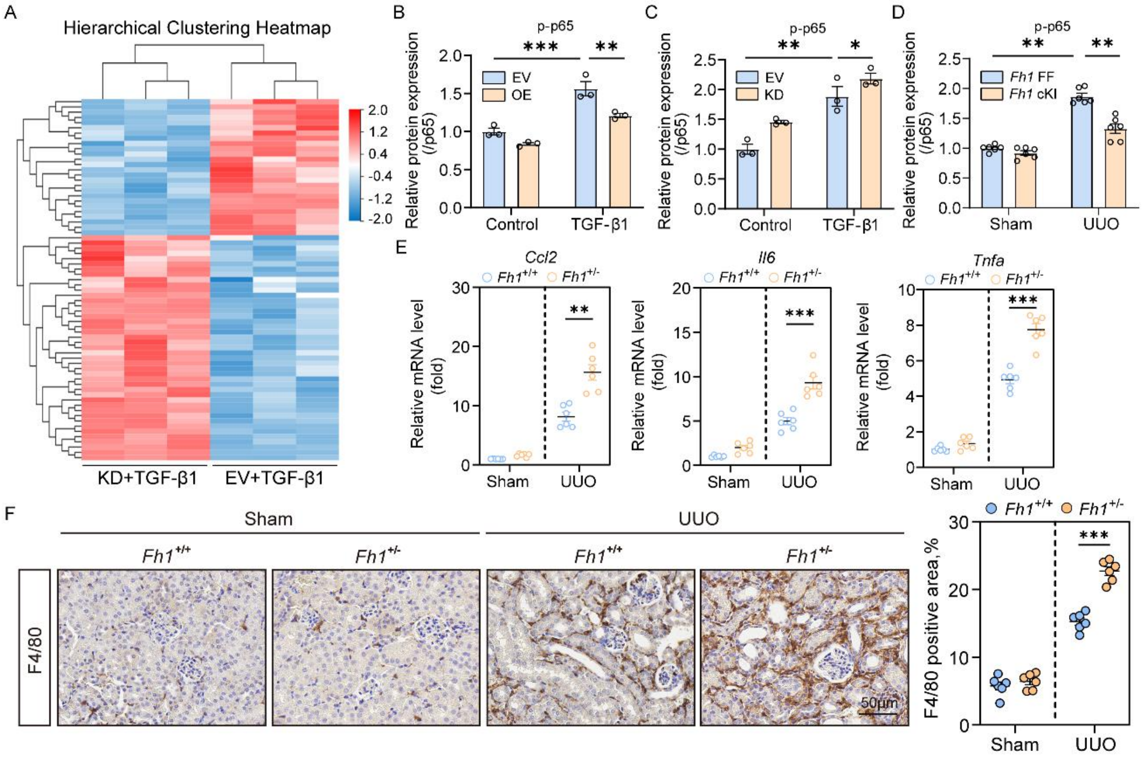

Supplemental Figure 10. FH loss is associated with enhanced renal inflammatory responses.

(**A**) Hierarchical clustering heatmap of inflammation-related genes comparing *Fh1* KD + TGF-β1-treated TECs with EV + TGF-β1 controls. (**B–D**) Densitometric quantification of p-p65 protein levels from the corresponding immunoblots using ImageJ. (**E**) qRT-PCR analysis of inflammatory cytokine mRNA levels in kidneys from *Fh1*$^{+/+}$ and *Fh1*$^{+/–}$ mice after sham surgery or UUO (n = 6). (**F**) Representative IHC staining for F4/80 in kidneys of *Fh1*$^{+/+}$ and *Fh1*$^{+/–}$ mice after sham operation or UUO (n = 6). Scale bar, 50 μm. Data are shown as mean ± SEM. Statistical analysis was performed using 2-way ANOVA followed by Tukey's multiple-comparisons test (**B–F**). *$P < 0.05$, **$P < 0.01$, and ***$P < 0.001$.

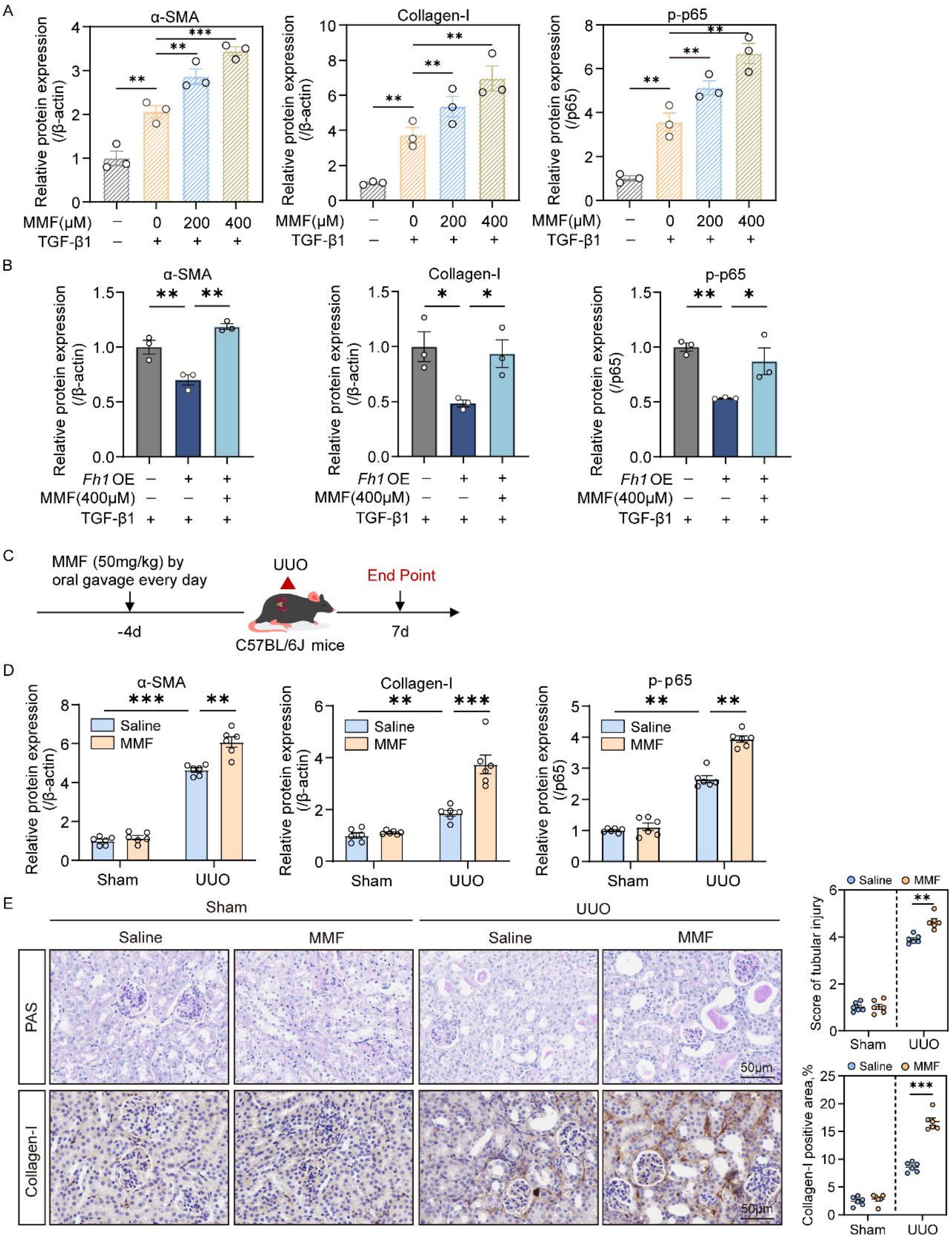

Supplemental Figure 11. Fumarate is associated with enhanced renal inflammation and fibrosis.

(**A**, **B** and **D**) Densitometric quantification of α-SMA, collagen-I, and p-p65 protein levels from the corresponding immunoblots using ImageJ. (**C**) Schematic diagram of the animal experiment design. (**E**) Representative PAS and IHC staining for collagen-I in kidneys from control and MMF-treated mice after sham operation or UUO (n = 6). Scale bar, 50 μm. Data are shown as mean ± SEM. Statistical analysis was performed using 1-way ANOVA followed by Tukey's multiple-comparisons test (**A** and **B**) and 2-way ANOVA followed by Tukey's multiple-comparisons test (**D** and **E**). *$P < 0.05$, **$P < 0.01$, and ***$P < 0.001$.

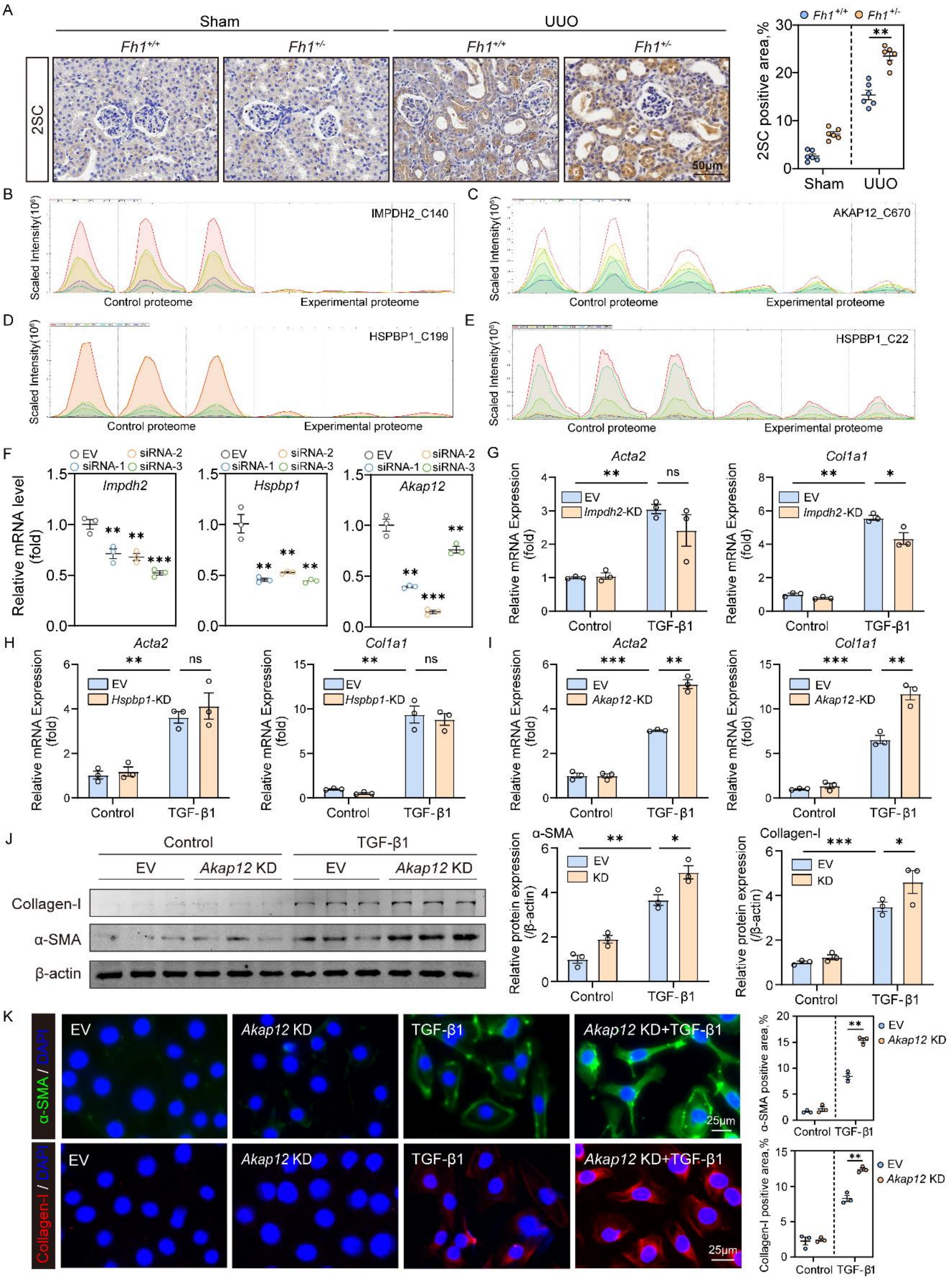

Supplemental Figure 12. AKAP12 is a key downstream effector in FH deficiency–

associated renal fibrosis.

(**A**) Representative IHC staining for 2SC in kidneys from *Fh1*$^{+/+}$ and *Fh1*$^{+/–}$ mice after sham surgery or UUO (n = 6). Scale bar, 50 μm. (**B–E**) Extracted ion chromatograms (XICs) of candidate fumarate-sensitive cysteine sites in control and experimental groups (**F**) qRT-PCR analysis of *Impdh2*, *Hspbp1*, and *Akap12* mRNA levels after siRNA-mediated knockdown (n = 3). (**G–I**) qRT-PCR analysis of *Acta2* and *Col1a1* mRNA expression after knockdown of *Impdh2*, *Hspbp1*, or *Akap12* in TECs with or without TGF-β1 treatment (n = 3). (**J**) Immunoblot analysis of α-SMA and collagen-I protein levels in EV and *Akap12* KD cells with or without TGF-β1 stimulation (n = 3). (**K**) Representative IF staining for α-SMA and collagen-I in EV and *Akap12* KD cells with or without TGF-β1 treatment (n = 3). Scale bar, 25 μm. Data are shown as mean ± SEM. Statistical analysis was performed using 1-way ANOVA followed by Tukey's multiple-comparisons test (**F**) and 2-way ANOVA followed by Tukey's multiple-comparisons test (**A** and **G–K**). **P* < 0.05, ***P* < 0.01, and ****P* < 0.001.

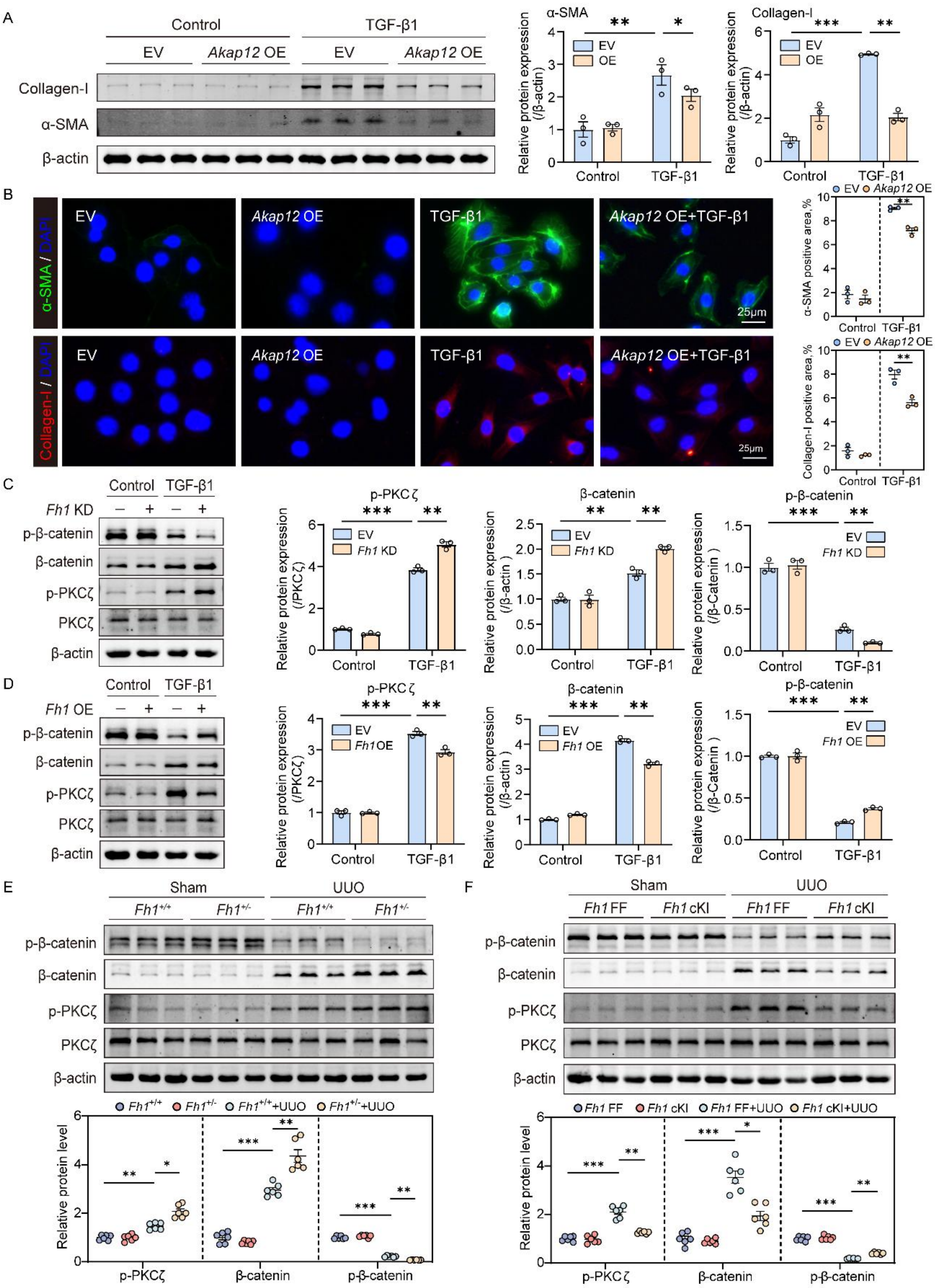

Supplemental Figure 13. Fumarate promotes renal fibrosis through AKAP12

succination and activation of PKCζ/β-catenin signaling.

(**A**) Immunoblot analysis of α-SMA and collagen-I in EV and *Akap12* OE cells with or without TGF-β1 stimulation (n = 3). (**B**) Representative IF staining for α-SMA and collagen-I in EV and *Akap12* OE cells with or without TGF-β1 treatment (n = 3). Scale bar, 25 μm. (**C** and **D**) Immunoblot analysis of p-PKCζ and p-β-catenin in EV and *Fh1* KD or *Fh1* OE cells with or without TGF-β1 treatment (n = 3). (**E**) Immunoblot analysis of p-PKCζ and p-β-catenin in kidneys from $Fh1^{+/+}$ and $Fh1^{+/-}$ mice after sham operation or UUO (n = 6). (**F**) Immunoblot analysis of p-PKCζ and p-β-catenin in kidneys from *Fh1* FF and *Fh1* cKI mice after sham operation or UUO (n = 6). Data are shown as mean ± SEM. Statistical analysis was performed using 2-way ANOVA followed by Tukey's multiple-comparisons test (**A**–**F**). *$P < 0.05$, **$P < 0.01$, and ***$P < 0.001$.

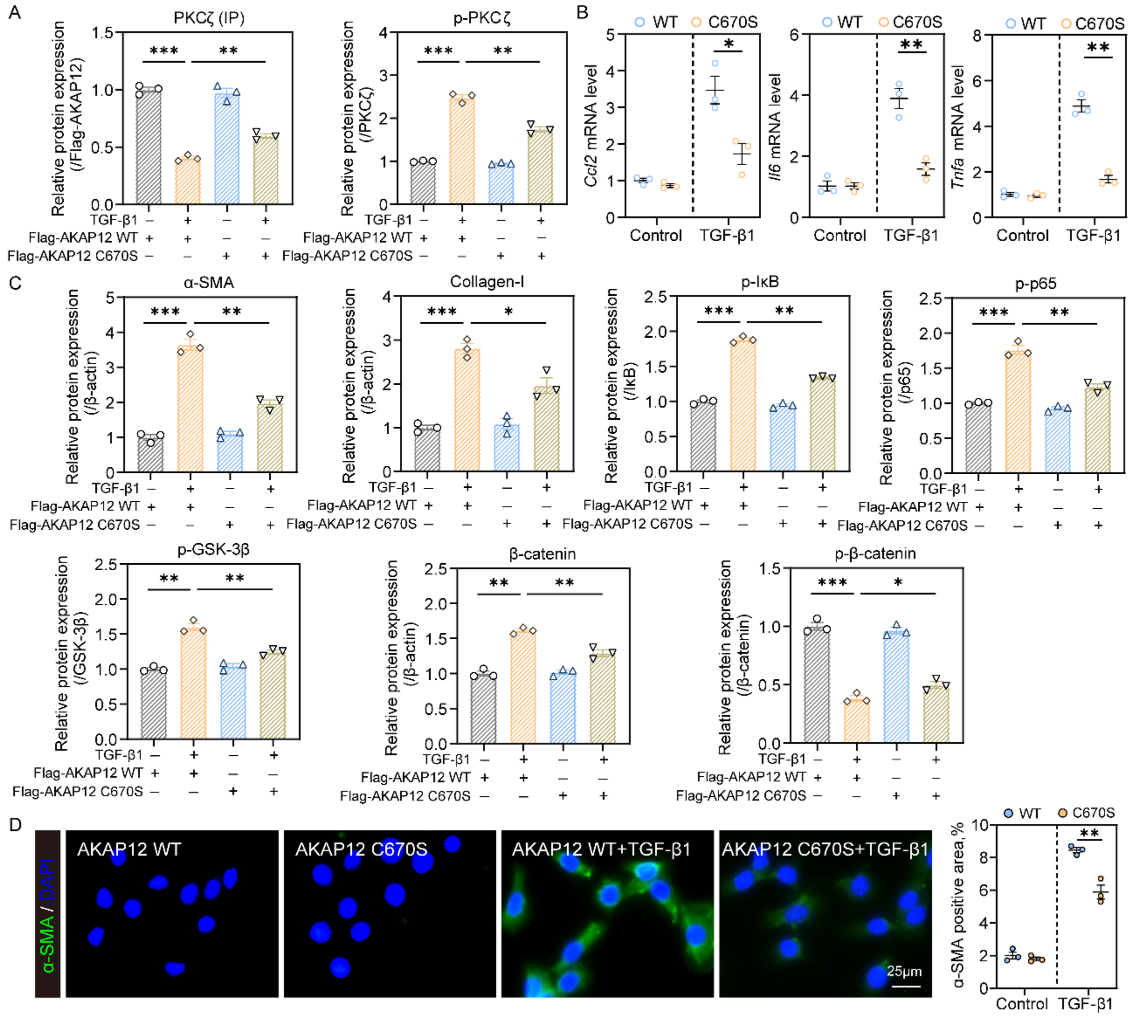

Supplemental Figure 14. AKAP12 C670S mutation attenuates TGF-β1-induced inflammatory and fibrotic responses in TECs.

(**A**) Densitometric quantification of PKCζ and p-PKCζ protein levels from the corresponding immunoblots using ImageJ. (**B**) qRT-PCR analysis of inflammatory gene expression in AKAP12 WT and AKAP12 C670S cells with or without TGF-β1 stimulation (n = 3). (**C**) Densitometric quantification of α-SMA, collagen-I, p-IκB, p-p65, p-GSK-3β, β-catenin, and p-β-catenin protein levels from the corresponding immunoblots using

ImageJ. (**D**) Representative IF staining for α-SMA in AKAP12 WT and AKAP12 C670S cells with or without TGF-β1 treatment (n = 3). Scale bar, 25 μm. Data are shown as mean ± SEM. Statistical analysis was performed using 2-way ANOVA followed by Tukey's multiple-comparisons test (**A**–**D**). $*P < 0.05$, $**P < 0.01$, and $***P < 0.001$.

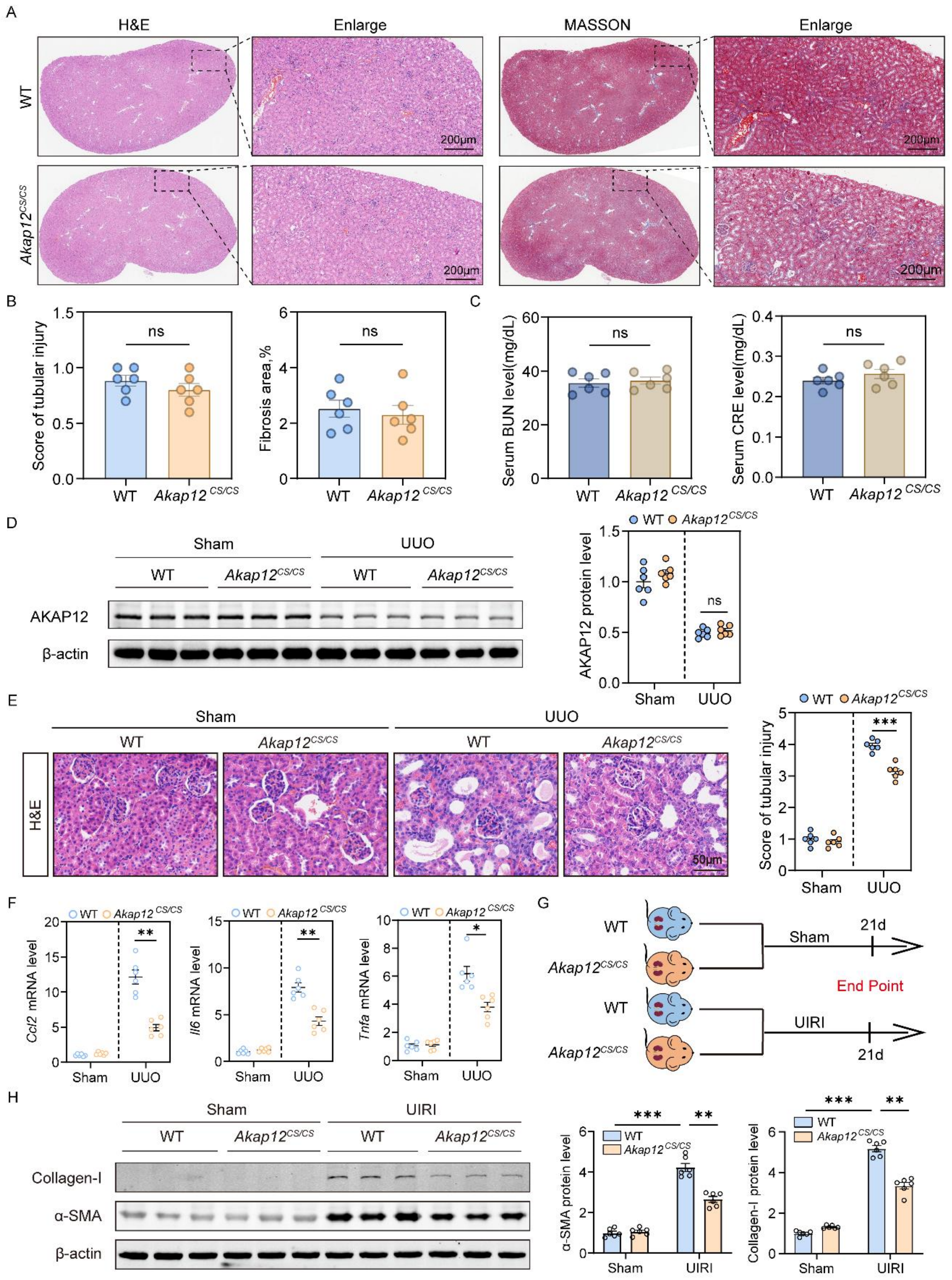

Supplemental Figure 15. ***Akap12*$^{CS/CS}$ mice are protected against UUO- and UIRI-**

induced renal inflammation and fibrosis.

(**A** and **B**) Representative H&E and Masson's trichrome staining of kidneys from WT and *Akap12*$^{CS/CS}$ mice (n = 6). Scale bar, 200 μm. (**C**) Serum BUN and CRE levels in WT and *Akap12*$^{CS/CS}$ mice. (**D**) Western blot analysis of AKAP12 protein levels in kidneys from WT and *Akap12*$^{CS/CS}$ mice after sham operation or UUO (n = 6). (**E**) Representative H&E staining of kidneys from WT and *Akap12*$^{CS/CS}$ mice after sham operation or UUO (n = 6). Scale bar, 50 μm. (**F**) qRT-PCR analysis of inflammatory gene expression in kidneys from WT and *Akap12*$^{CS/CS}$ mice after sham operation or UUO (n = 6). (**G**) Schematic overview of the animal experimental design. (**H**) Western blot analysis of α-SMA and collagen-I protein levels in kidneys from WT and *Akap12*$^{CS/CS}$ mice after sham operation or UIRI (n = 6). Data are shown as mean ± SEM. Statistical analysis was performed using 2-tailed unpaired Student's *t* test (**B** and **C**) and 2-way ANOVA followed by Tukey's multiple-comparisons test (**D**–**F** and **H**). *$P < 0.05$, **$P < 0.01$, and ***$P < 0.001$.

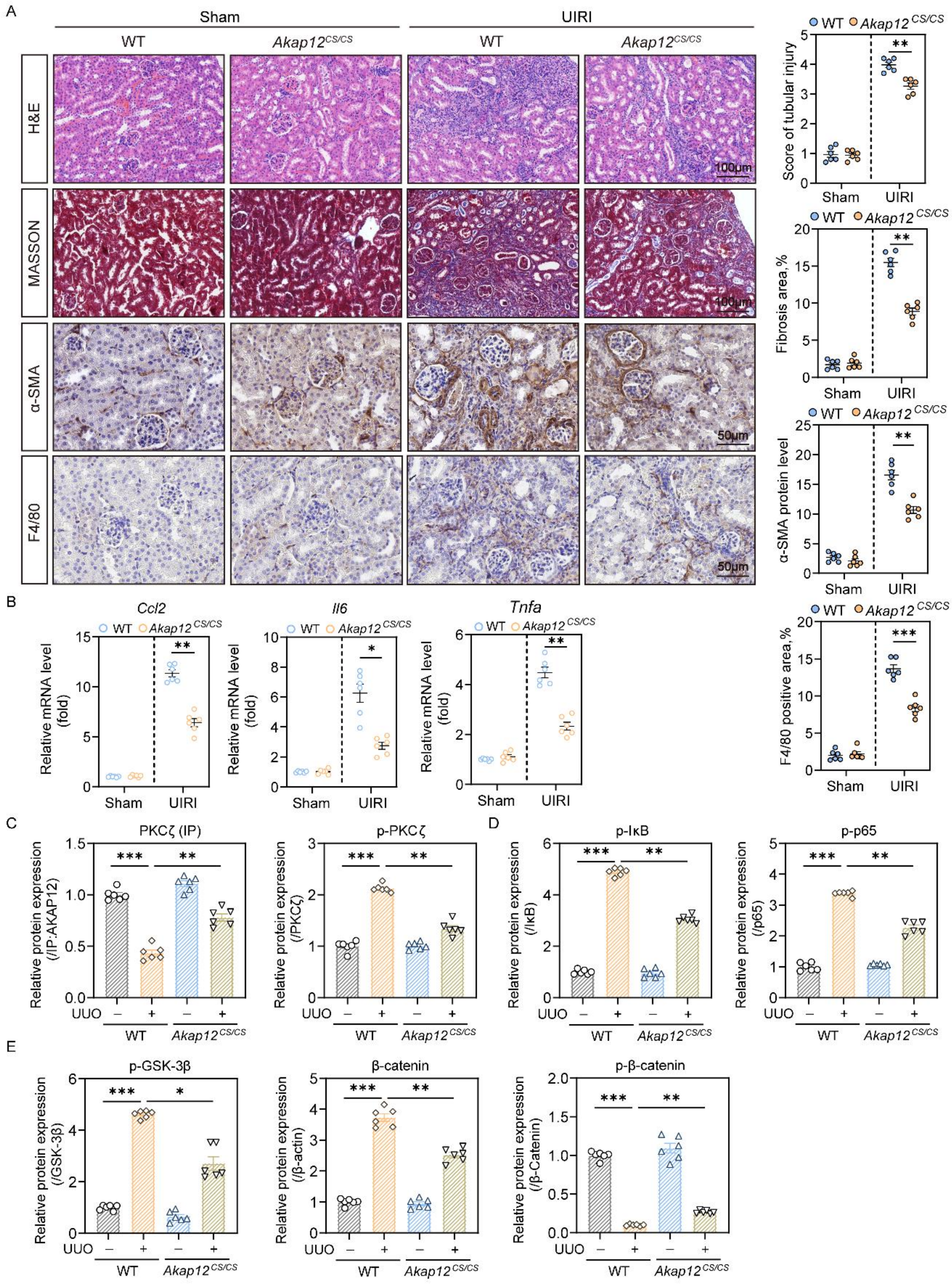

Supplemental Figure 16. ***Akap12$^{CS/CS}$*** **mice show reduced activation of PKCζ/NF-κB**

and PKCζ/β-catenin signaling after renal injury.

(**A**) Representative H&E, Masson's trichrome, and IHC staining for α-SMA and F4/80 in kidneys from WT and *Akap12*$^{CS/CS}$ mice after sham operation or UIRI (n = 6). Scale bar, 50/100 μm. (**B**) qRT-PCR analysis of inflammatory gene expression in kidneys from WT and *Akap12*$^{CS/CS}$ mice after sham operation or UIRI (n = 6). (**C**) Densitometric quantification of PKCζ and p-PKCζ protein levels from the corresponding immunoblots using ImageJ. (**D** and **E**) Densitometric quantification of p-IκB, p-p65, p-GSK-3β, β-catenin, and p-β-catenin protein levels from the corresponding immunoblots using ImageJ. Data are shown as mean ± SEM. Statistical analysis was performed using 2-way ANOVA followed by Tukey's multiple-comparisons test (**A**–**E**). *$P < 0.05$, **$P < 0.01$, and ***$P < 0.001$.

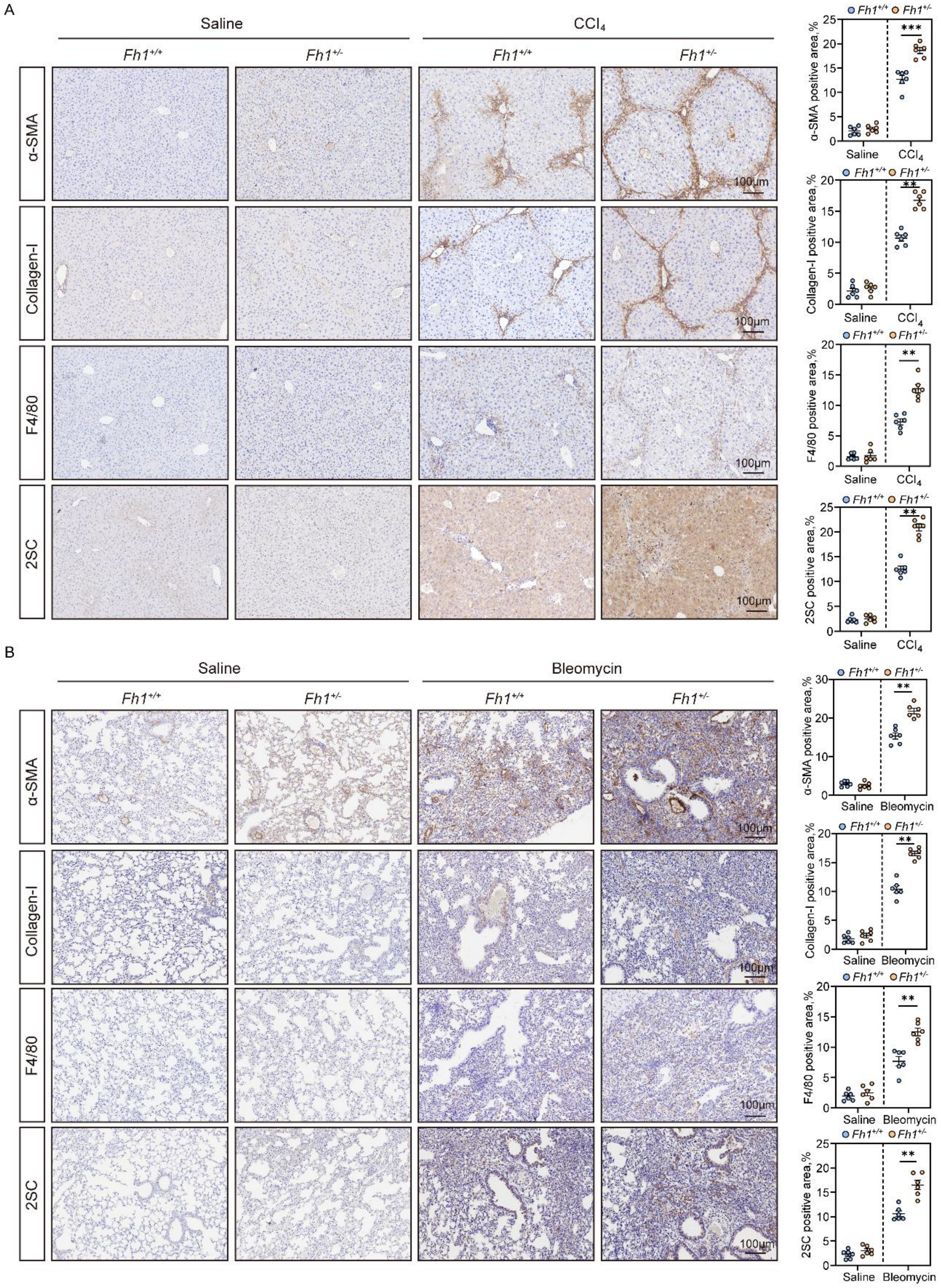

Supplemental Figure 17. Loss of *Fh1* exacerbates liver and pulmonary fibrosis.

(**A**) Representative IHC staining for α-SMA, collagen-I, F4/80, and 2SC in livers from *Fh1*$^{+/+}$ and *Fh1*$^{+/-}$ mice with or without CCl_4-induced fibrosis (n = 6). Scale bars, 100 μm. (**B**) Representative IHC staining for α-SMA, collagen-I, F4/80, and 2SC in lungs from *Fh1*$^{+/+}$ and *Fh1*$^{+/-}$ mice with or without bleomycin-induced fibrosis (n = 6). Scale bars, 100 μm. Data are shown as mean ± SEM. Statistical analysis was performed using 2-way ANOVA followed by Tukey's multiple-comparisons test (**A** and **B**). **P* < 0.05, ***P* < 0.01, and ****P* < 0.001.

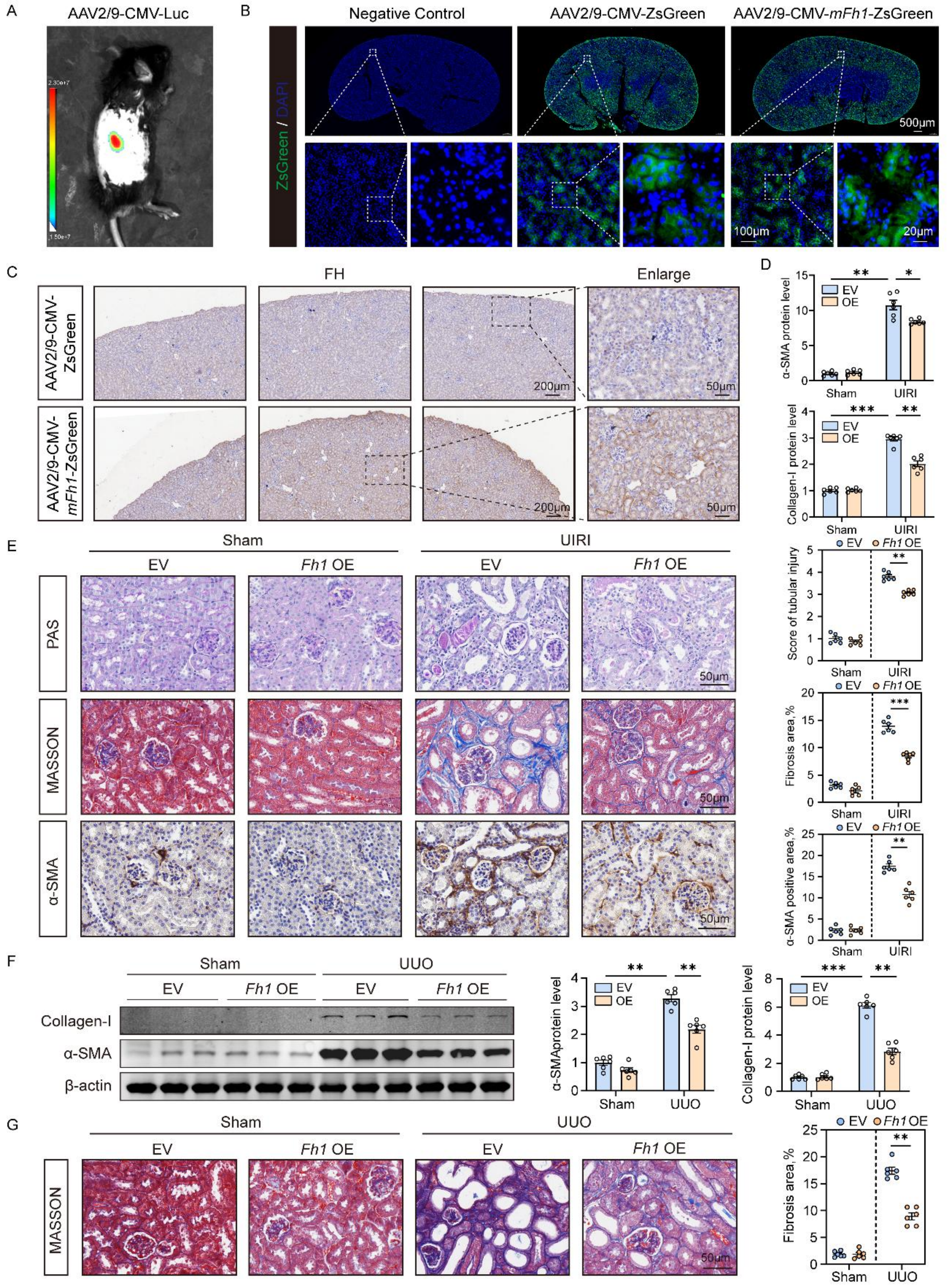

Supplemental Figure 18. Renal *in situ* delivery of AAV2/9-mediated FH overexpression

alleviates renal fibrosis.

(**A**) Representative *in vivo* bioluminescence images of mice injected with AAV2/9-CMV-Luc. (**B**) Representative ZsGreen fluorescence in the renal cortex after injection of AAV2/9-CMV-ZsGreen or AAV2/9-CMV-*mFh1*-ZsGreen. Scale bar: 500 μm, 100 μm, and 20 μm. (**C**) Representative IHC staining for FH in the renal cortex. Scale bar, 200 μm; inset, 50 μm. (**D**) Densitometric quantification of α-SMA and collagen-I protein levels from the corresponding immunoblots using ImageJ. (**E**) Representative PAS, Masson's trichrome, and IHC staining for α-SMA in kidneys from EV and *Fh1* OE mice after sham operation or UIRI (n = 6). Scale bar, 50 μm. (**F**) Immunoblot analysis of α-SMA and collagen-I in kidneys from EV and *Fh1* OE mice after sham operation or UUO (n = 6). (**G**) Representative Masson's trichrome staining of kidneys from EV and *Fh1* OE mice after sham operation or UUO (n = 6). Scale bar, 50 μm. Data are shown as mean ± SEM. Statistical analysis was performed using 2-way ANOVA followed by Tukey’s multiple-comparisons test (**D**–**G**). *$P < 0.05$, **$P < 0.01$, and ***$P < 0.001$.

Supplemental Table 1: Clinical features of patients in this study.

Diagnosis	Sex	Age	Serum creatinine, µmol/L	Serum urea, mmol/L	Serum fumarate, ng/µL	Urine fumarate, ng/µL	eGFR, mL/min/1.73m^2	Sample
Normal	Male	25	NA	NA	21.01384536	2.99177662	104	Serum, urine
Normal	Male	26	NA	NA	20.78170508	6.028566411	112	Serum, urine
Normal	Male	25	NA	NA	22.912366	6.97063056	100	Serum, urine
Normal	Male	26	NA	NA	22.27656845	4.895195987	106	Serum, urine
Normal	Female	25	NA	NA	24.01983655	7.750325157	113	Serum, urine
Normal	Female	23	NA	NA	26.34271548	4.192667029	97	Serum, urine
Normal	Female	24	NA	NA	52.63959412	26.30223364	113	Serum, urine
CKD	Male	35	83	5.58	36.51990983	8.229395205	111	Serum, urine, kidney biopsy sample
CKD	Male	55	52	4.35	47.06971208	10.71798794	109	Serum, urine, kidney biopsy sample
CKD	Female	59	58	6.28	54.75694774	34.54288214	102	Serum, urine, kidney biopsy sample
CKD	Male	56	63	4.15	47.49406982	9.092685926	111	Serum, urine, kidney biopsy sample
CKD	Male	72	70	4.74	37.13204904	18.10499087	95	Serum, urine, kidney biopsy sample
CKD	Female	65	81.3	5.38	55.95165493	16.28677549	69	Serum, urine, kidney biopsy sample
CKD	Male	41	118	5.16	56.08177269	23.57089097	70	Serum, urine, kidney biopsy sample

CKD	Male	44	104	6.77	62.88332394	21.99060287	80	Serum, urine, kidney biopsy sample
CKD	Male	53	102.6	6.65	32.70216895	14.05540187	76	Serum, urine, kidney biopsy sample
CKD	Male	50	105.5	6.18	62.00208022	24.89717625	75	Serum, urine, kidney biopsy sample
CKD	Male	50	175	10.22	78.1927367	31.81475433	41	Serum, urine, kidney biopsy sample
CKD	Female	59	130	14.91	84.04502882	50.94058286	41	Serum, urine, kidney biopsy sample
CKD	Male	73	137	6.6	68.23437514	36.61510116	47	Serum, urine, kidney biopsy sample
CKD	Male	32	197.4	7.72	71.78449071	47.39578598	40	Serum, urine, kidney biopsy sample
CKD	Male	51	212	8.98	76.97289233	38.34650578	32	Serum, urine, kidney biopsy sample
CKD	Male	32	146	35.3	94.93195267	20.0694997	58	Serum, urine, kidney biopsy sample
CKD	Male	84	262	25.42	34.88162203	17.10666042	20	Serum, urine, kidney biopsy sample
CKD	Male	57	348.2	20.66	82.00899961	23.71075392	27	Serum, urine, kidney biopsy sample
CKD	Female	39	370	13.96	85.02238415	14.03771792	18	Serum, urine, kidney biopsy sample
CKD	Female	50	571.3	29.49	65.91593187	24.28949595	5	Serum, urine, kidney biopsy sample

CKD	Female	28	589.5	39.02	23.47275502	17.14524294	8	Serum, urine, kidney biopsy sample
CKD	Female	41	512.1	30.67	90.50650593	55.73932189	9	Serum, urine, kidney biopsy sample
CKD	Female	32	519.4	30.99	101.6714061	49.79917454	7	Serum, urine, kidney biopsy sample
Renal cell carcinoma	Female	53	NA	NA	NA	NA	NA	kidney biopsy sample
Renal cell carcinoma	Male	49	NA	NA	NA	NA	NA	kidney biopsy sample
Renal cell carcinoma	Male	51	NA	NA	NA	NA	NA	kidney biopsy sample
Renal cell carcinoma	Female	72	NA	NA	NA	NA	NA	kidney biopsy sample
Renal cell carcinoma	Female	64	NA	NA	NA	NA	NA	kidney biopsy sample
Renal cell carcinoma	Female	58	NA	NA	NA	NA	NA	kidney biopsy sample
Renal cell carcinoma	Female	77	NA	NA	NA	NA	NA	kidney biopsy sample

Supplemental Table 2. List of siRNA sequences used in this study.

siRNA sequences (mouse)

Terms	sense strand (5'-3')	antisense strand (5'-3')
Fh1 siRNA-1	CGUGUAGAGUUCGACACCUUUTT	AAAGGUGUCGAACUCUACACGTT
Fh1 siRNA-2	CCUCUUACUCUUGGACAGGAATT	UUCCUGUCCAAGAGUAAGAGGTT
Fh1 siRNA-3	GCUAAUGAAUGAGUCUUUAAUTT	AUUAAAGACUCAUUCAUUAGCTT
Klf9 siRNA	CCUCCCAUCUUAAAGCCCAUUTT	AAUGGGCUUUAAGAUGGGAGGTT
Bcl6 siRNA	CCUUAGUGUAAUCAAUCUATT	UAGAUUGAUUACACUAAGGTT
Impdh2 siRNA-1	UCGGAAAGUGAAGAAAUACTT	GUAUUUCUUCACUUUCCGATT
Impdh2 siRNA-2	UGGAAGAGAUCAUGACUAATT	UUAGUCAUGAUCUCUUCCATT
Impdh2 siRNA-3	AGGGAAAGUUGCCCAUUGUTT	ACAAUGGGCAACUUUCCCUTT
Hspbp1 siRNA-1	AGGUGGAGCAGAUGAAGAATT	UUCUUCAUCUGCUCCACCUTT
Hspbp1 siRNA-2	UGGACAAUGCAGCAGAUUUTT	AAAUCUGCUGCAUUGUCCATT
Hspbp1 siRNA-3	AAGUGCAGAAGCUCAAGGUTT	ACCUUGAGCUUCUGCACUUTT
Akap12 siRNA-1	GGAAGAAGUCACUGUUGAATT	UUCAACAGUGACUUCUUCCTT
Akap12 siRNA-2	GCUUCAAGAAGGUAUUUAATT	UUAAAUACCUUCUUGAAGCTT
Akap12 siRNA-3	GGUUGUCAGCCGACUAUGATT	UCAUAGUCGGCUGACAACCTT

Supplemental Table 3. List of qRT-PCR primers sequences used in this study.

Primers for qRT-PCR (mouse)

Terms	Forward primer (5'-3')	Reverse primer (5'-3')
Actb	CCAGCCTTCCTTCTTGGGTA	CAATGCCTGGGTACATGGTG
Fh1	GCTCGAATGACACCTTTCCC	GATGACCTGCGCAAACTCTT
Acta2	GTCCCTCTATGCCTCTGGAC	AAGGAATAGCCACGCTCAGT
Col1a1	TGGAAACCCGAGGTATGCTT	CATTGCATTGCACGTCATCG
Klf9	GCCGCCTACATGGACTTCG	GGTCACCGTGTTCCTTGGT
Bcl6	CCGGCACGCTAGTGATGTT	GCACTGTCTTATGGGCTCTAAAC
Pax3	AGAGAACCCGGGCATGTTTA	CTCAGGATGCGGCTGATAGA
Il6	GACTGATGCTGGTGACAACC	AGACAGGTCTGTTGGGAGTG
Tnfa	CCAACGGCATGGATCTCAAA	CCCTTGAAGAGAACCTGGGA
Ccl2	AACTGCATCTGCCCTAAGGT	CTGTCACACTGGTCACTCCT
Impdh2	ATTAGCGGAGGCACCTCTTAC	CAGTGAAGTCGATATACCCAGGA
Hspbp1	CTCCCTCTTGCGTTGCCTC	ACCCGCAGTAATAGCCATCTG
Akap12	CTGTCTGCCGTCAATGGTGTA	TGAAGCAGGGATCTGTTCGAT
