# Supplementary material for: Fumarate-induced succination of A-kinase anchor protein 12 exacerbates renal inflammation and fibrosis
Source: J Clin Invest. 2026 Jun 30;136(14):e200755. doi: 10.1172/JCI200755 (PMC13367978; doi:10.1172/JCI200755)

Unedited blot and gel images

Figure 1G

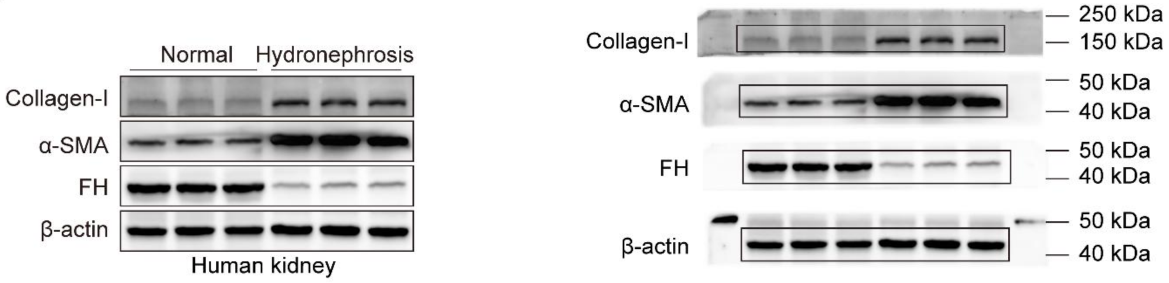

Figure 1H

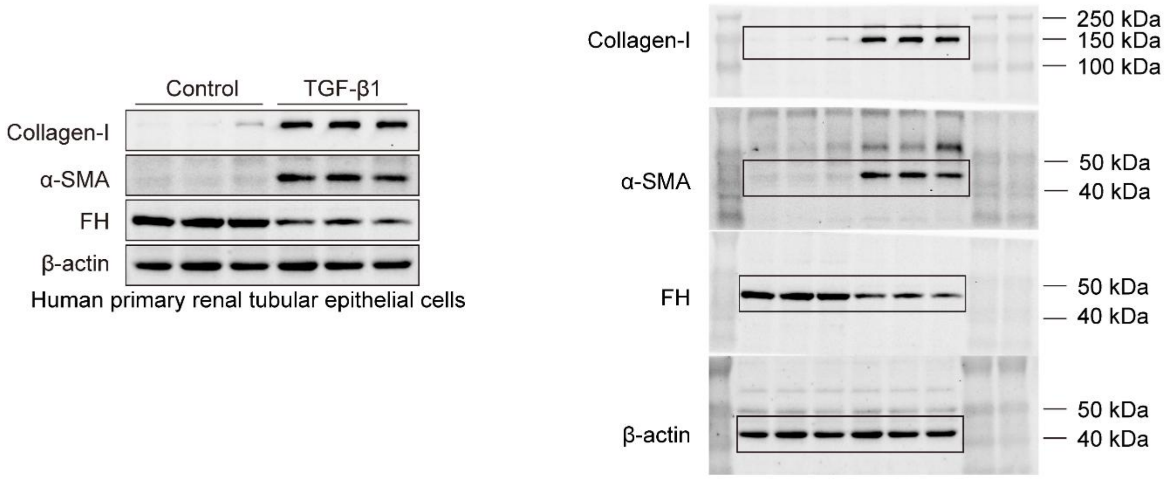

Figure 1I

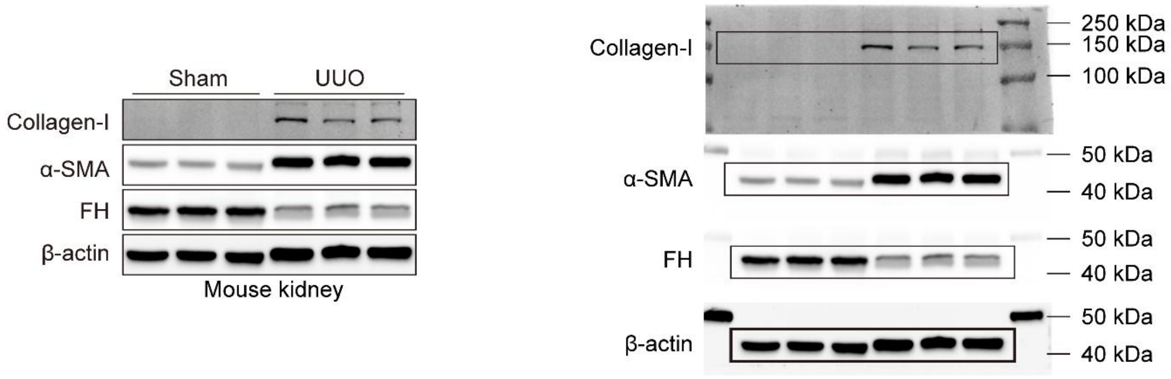

Figure 1J

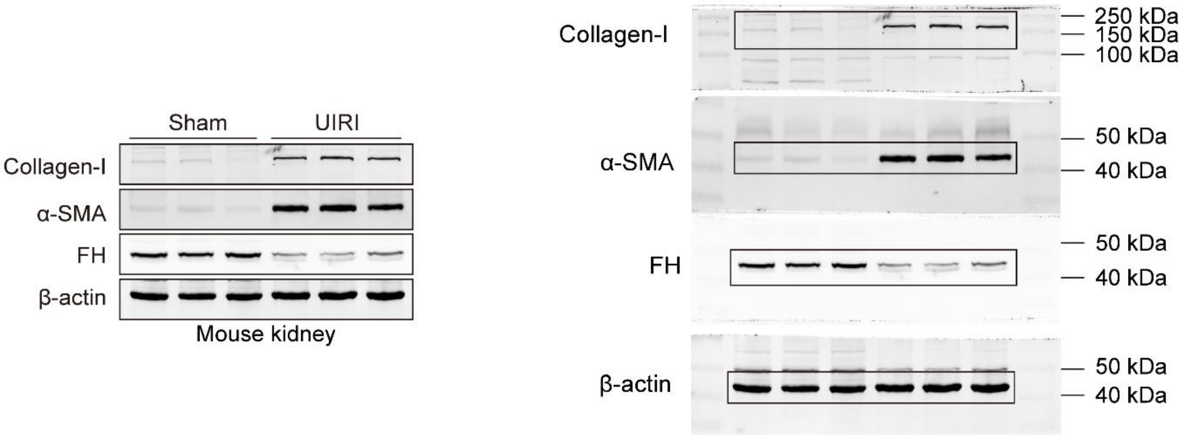

Figure 1K

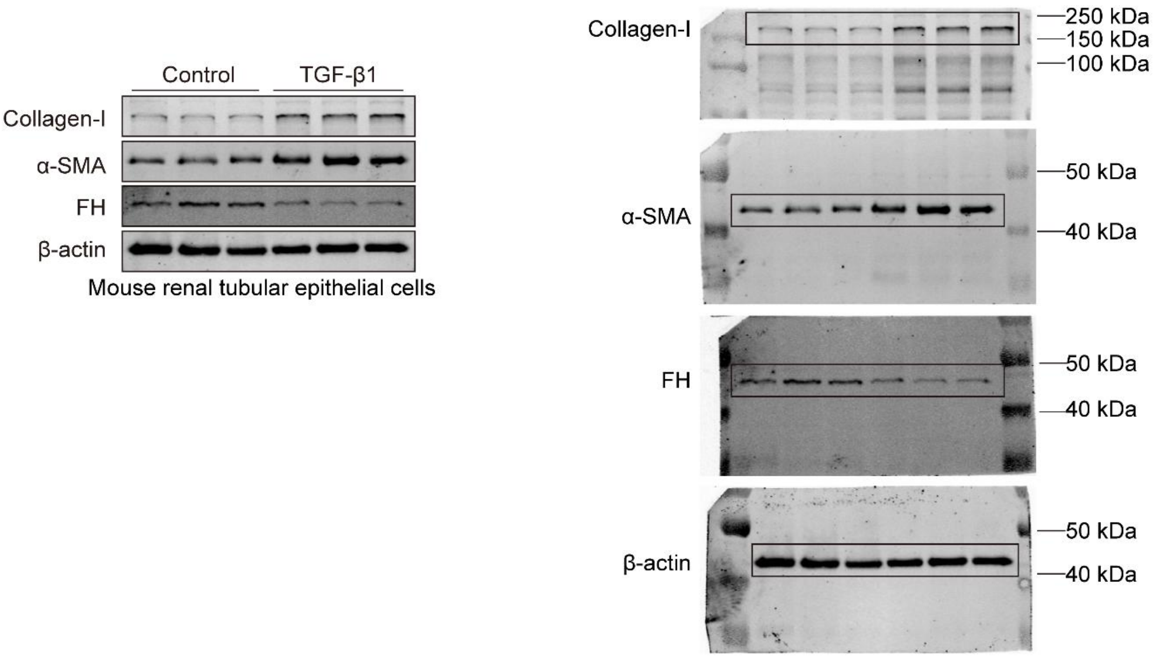

Figure 1L

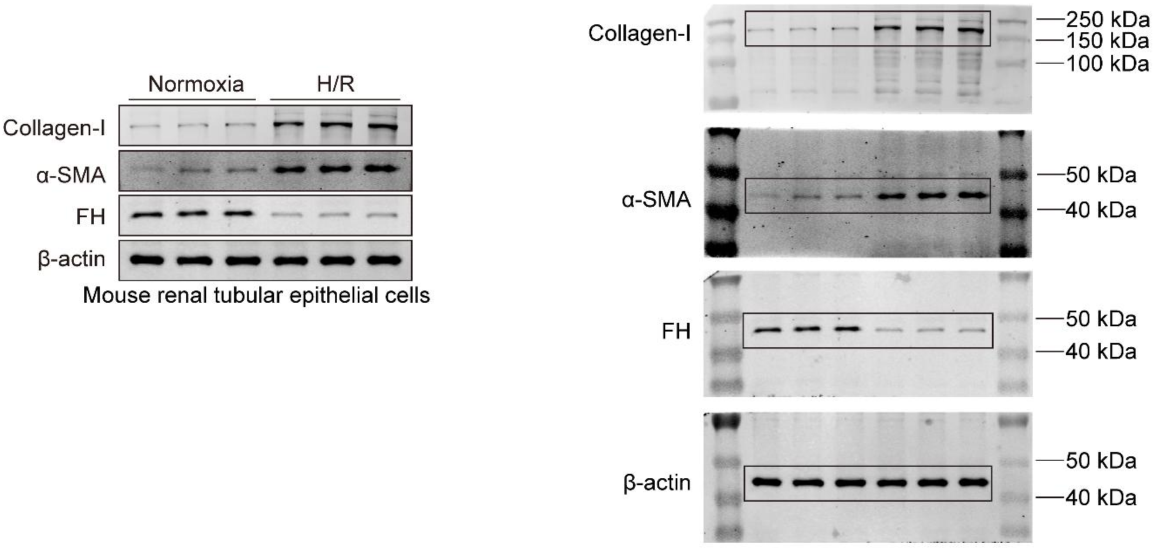

Figure 1O

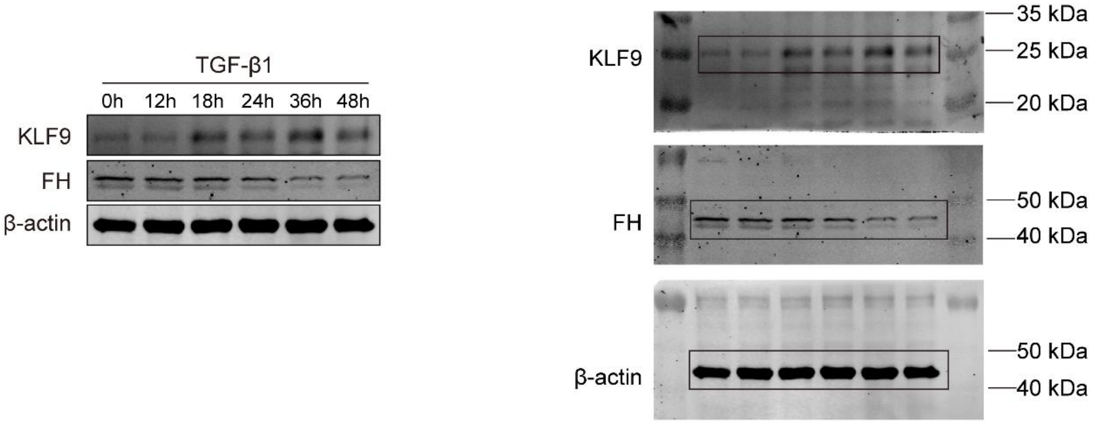

Figure 1P

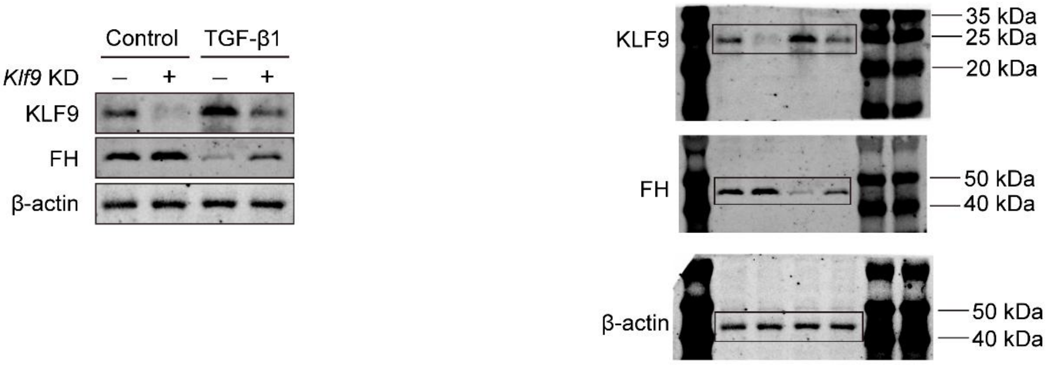

Figure 2B

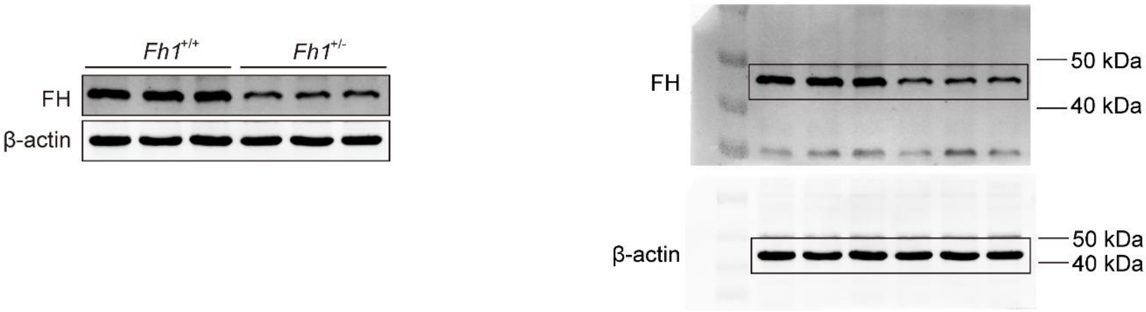

Figure 2E

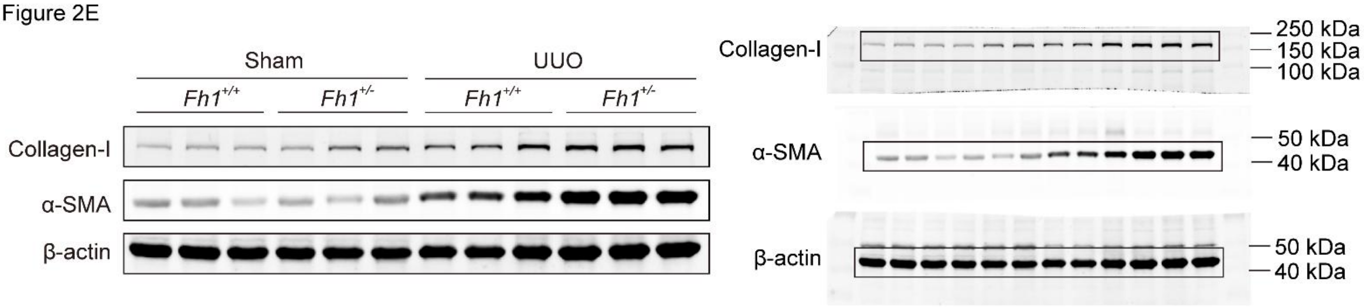

Figure 2H

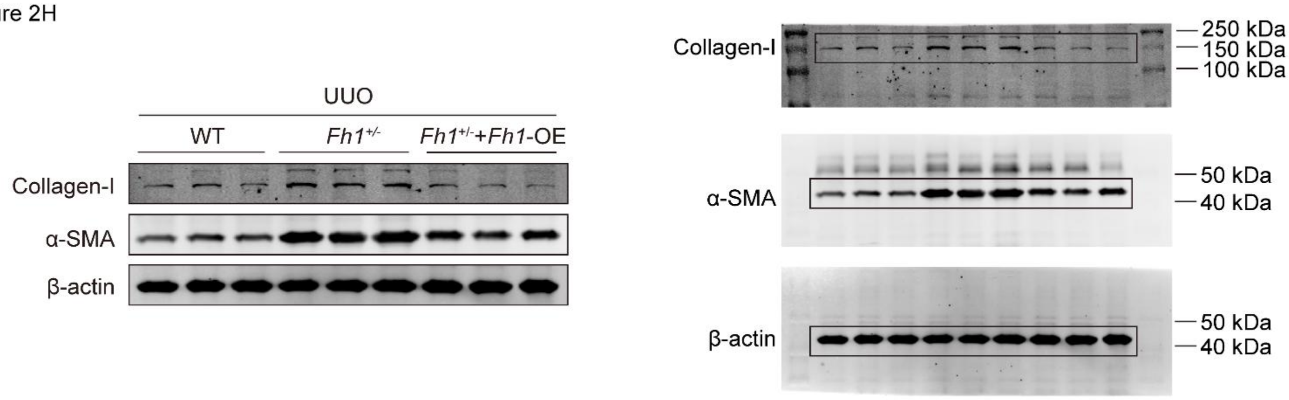

Figure 3E

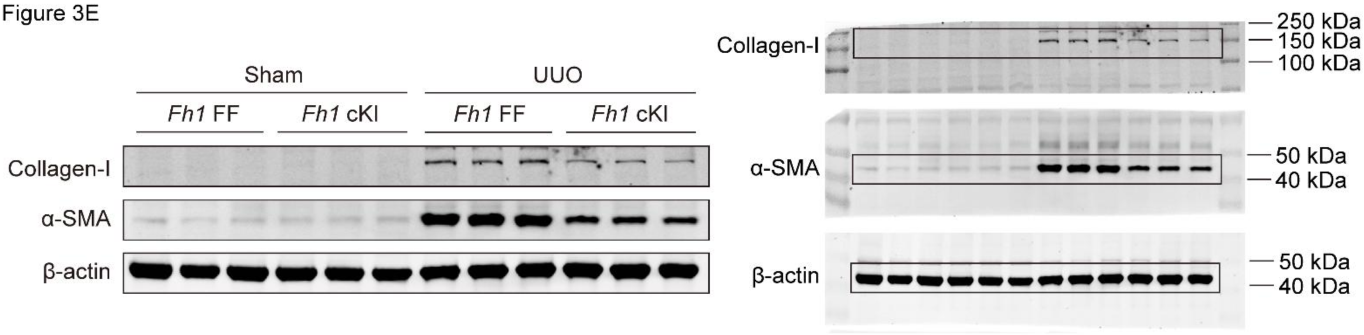

Figure 3H

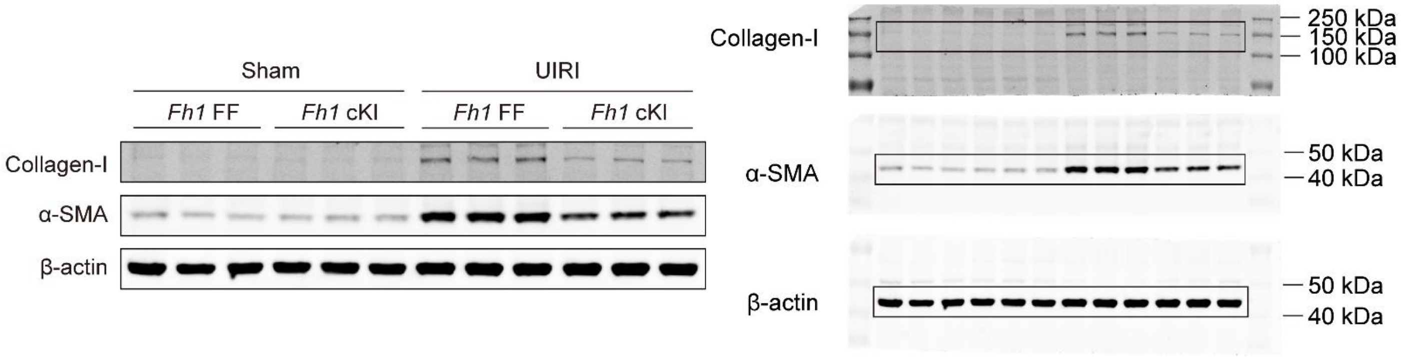

Figure 4H

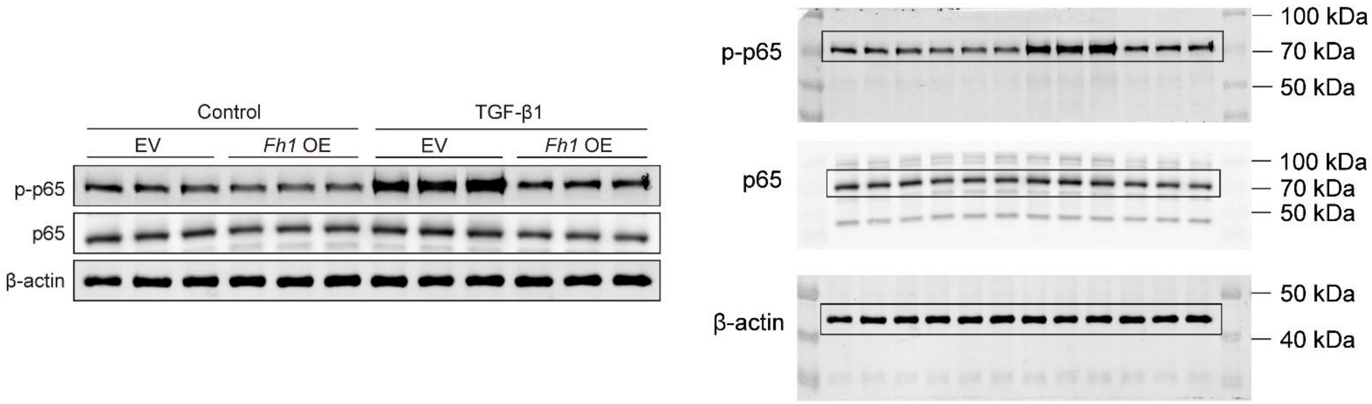

Figure 4I

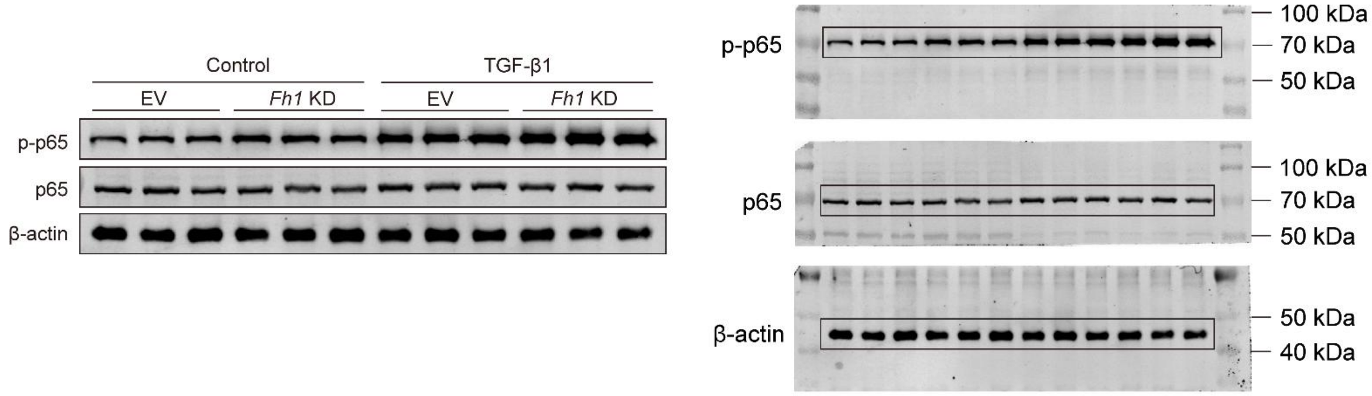

Figure 4J

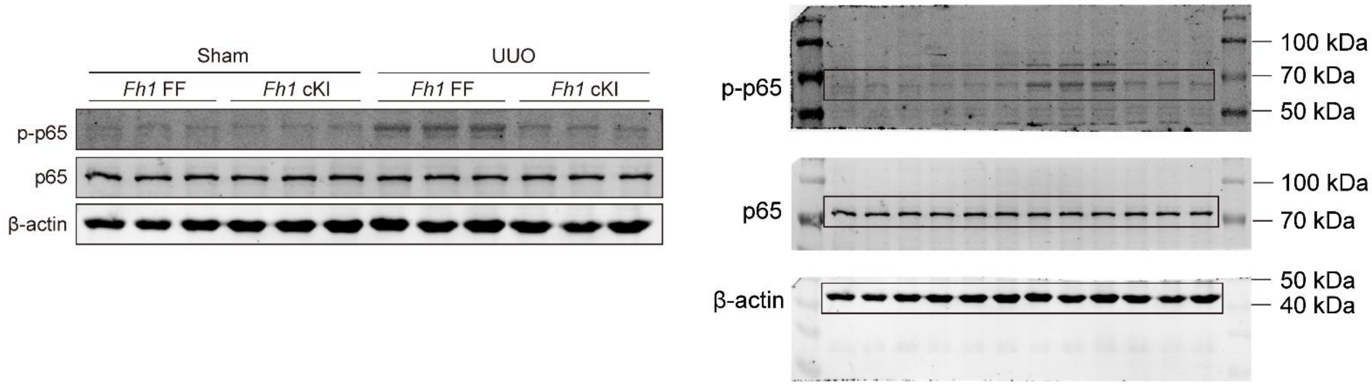

Figure 5D

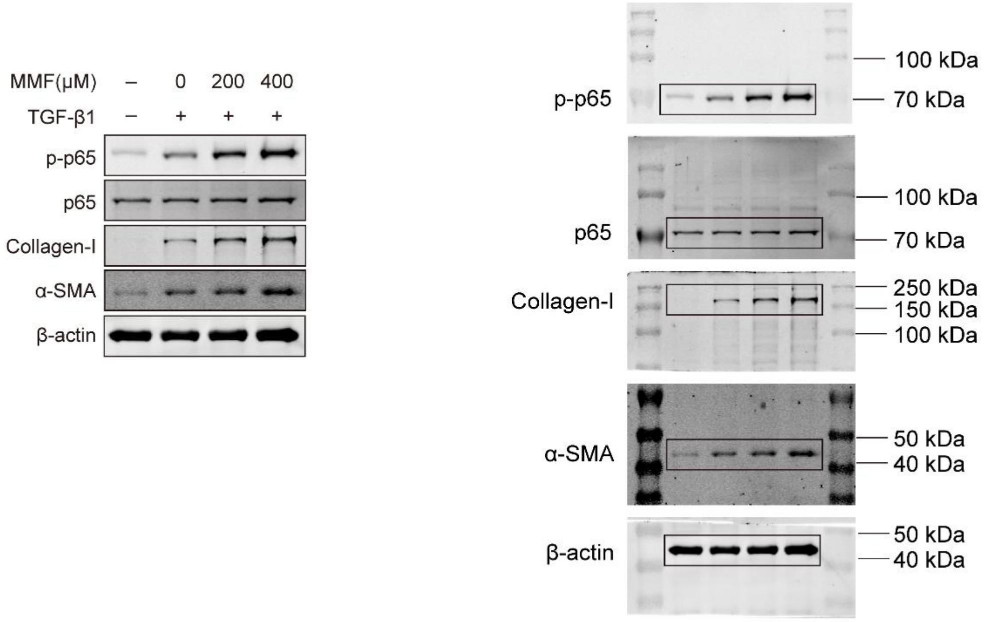

Figure 5F

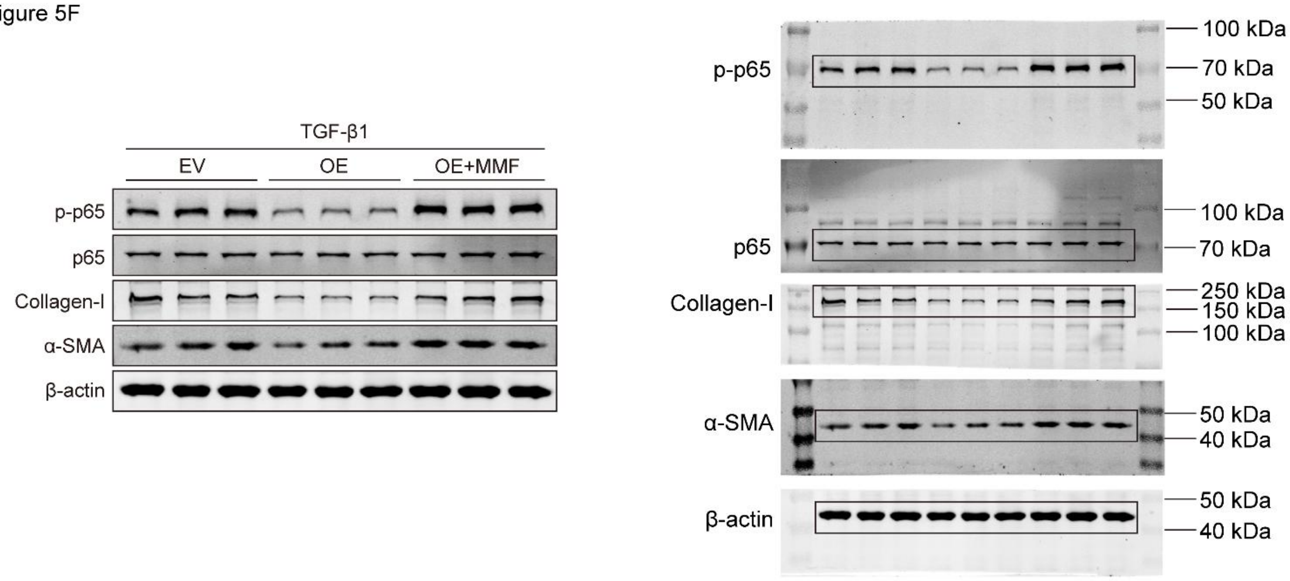

Figure 5I

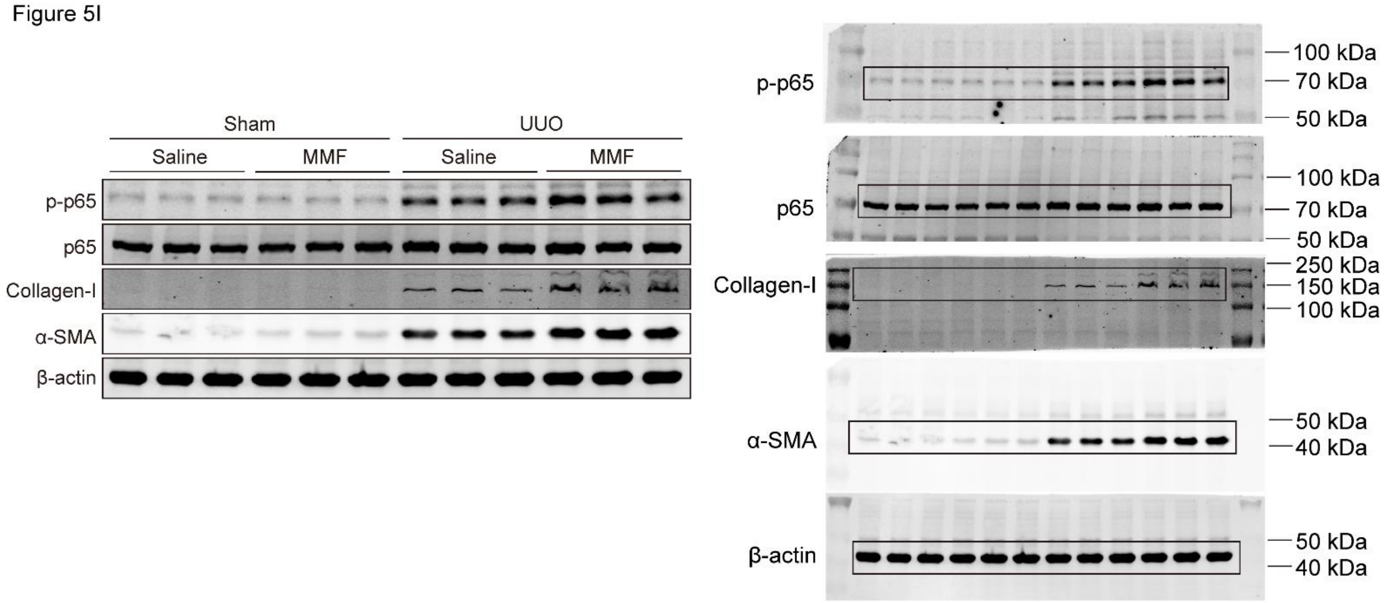

Figure 6E

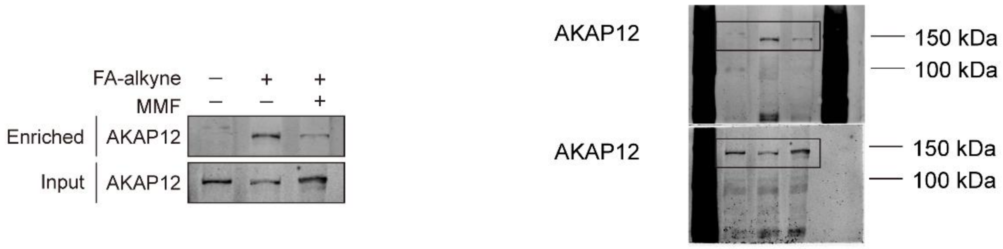

Figure 6F

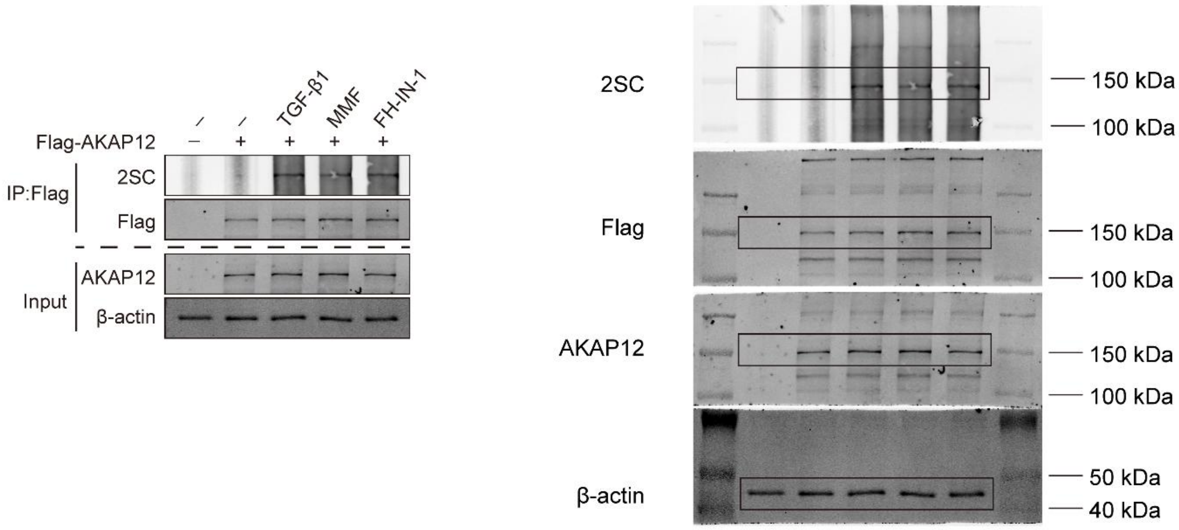

Figure 6H

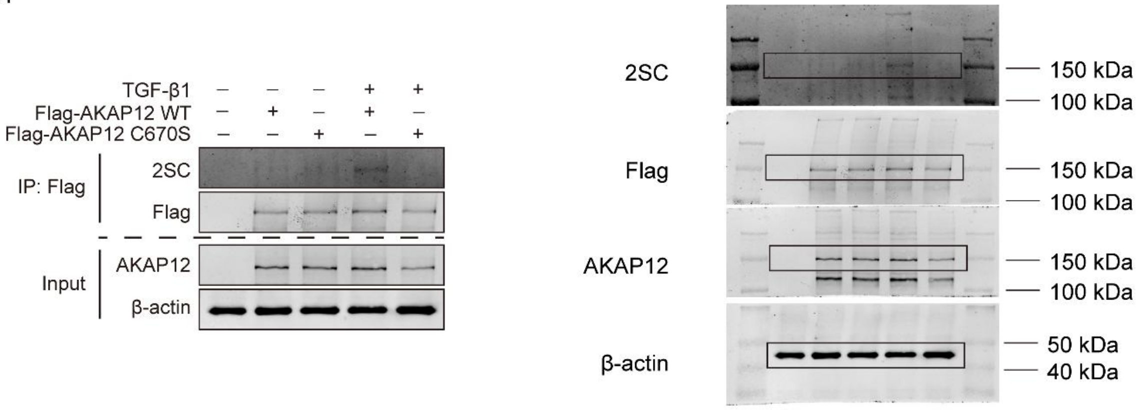

Figure 6I

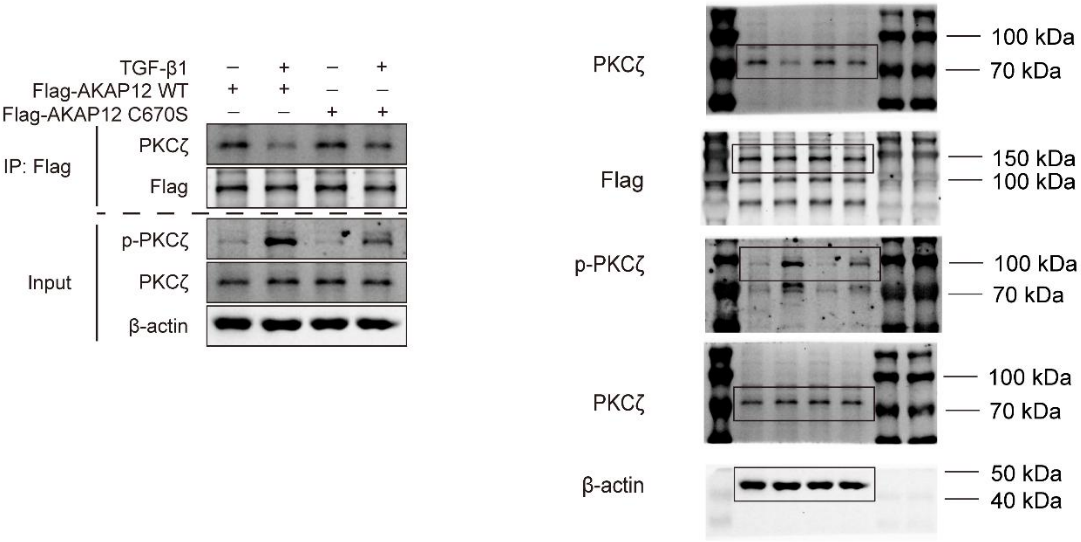

Figure 6J

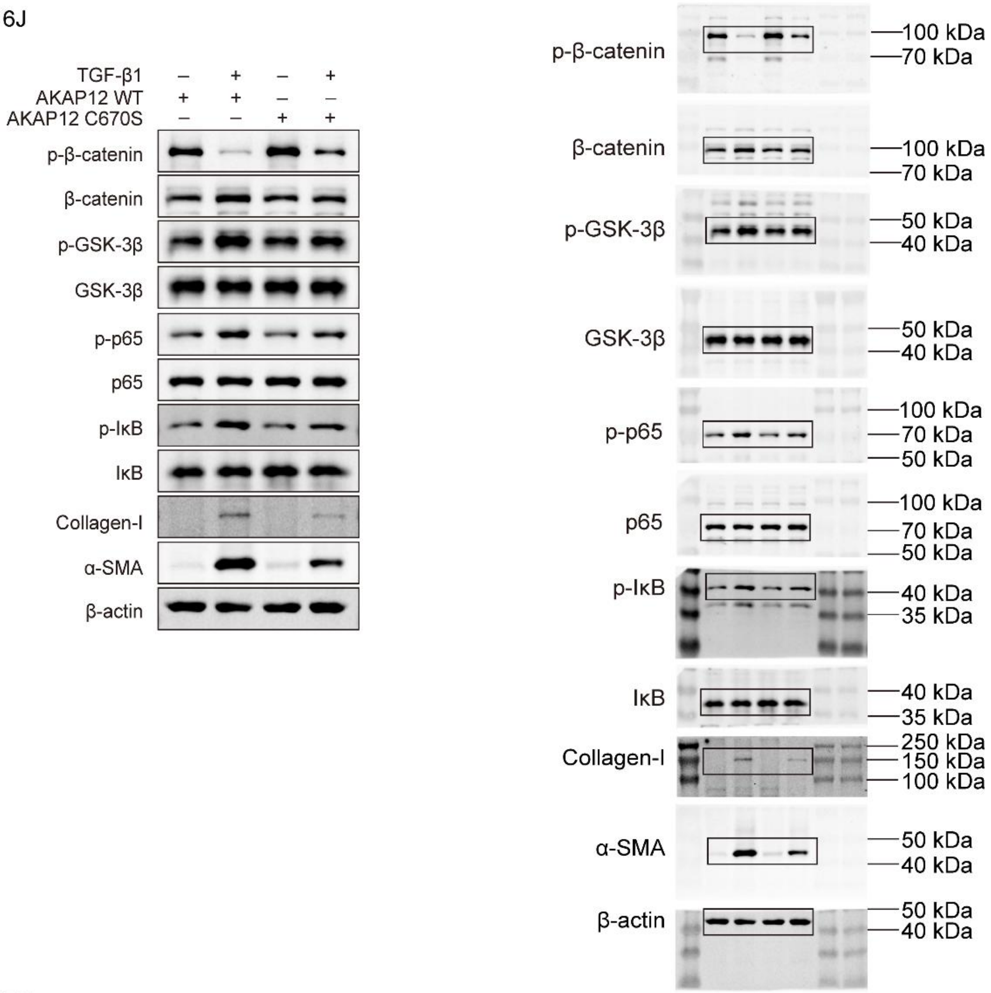

Figure 7E

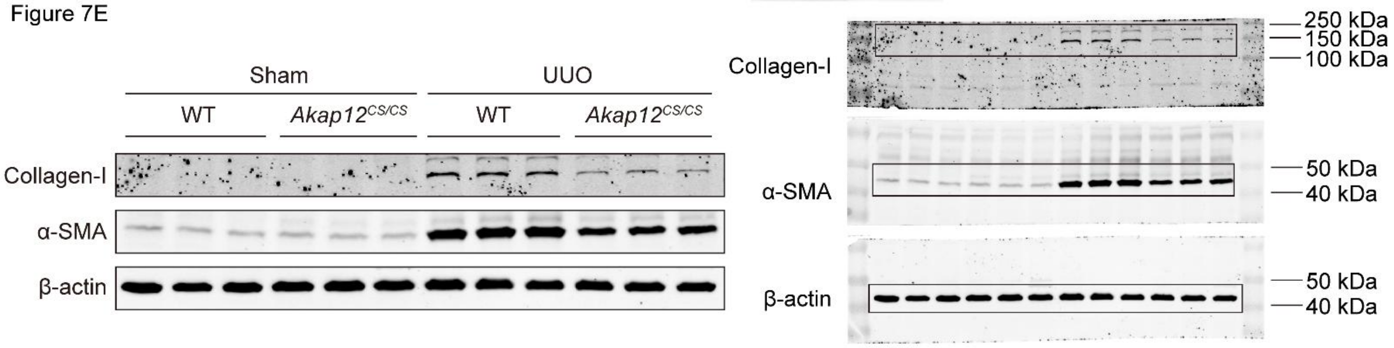

Figure 7F

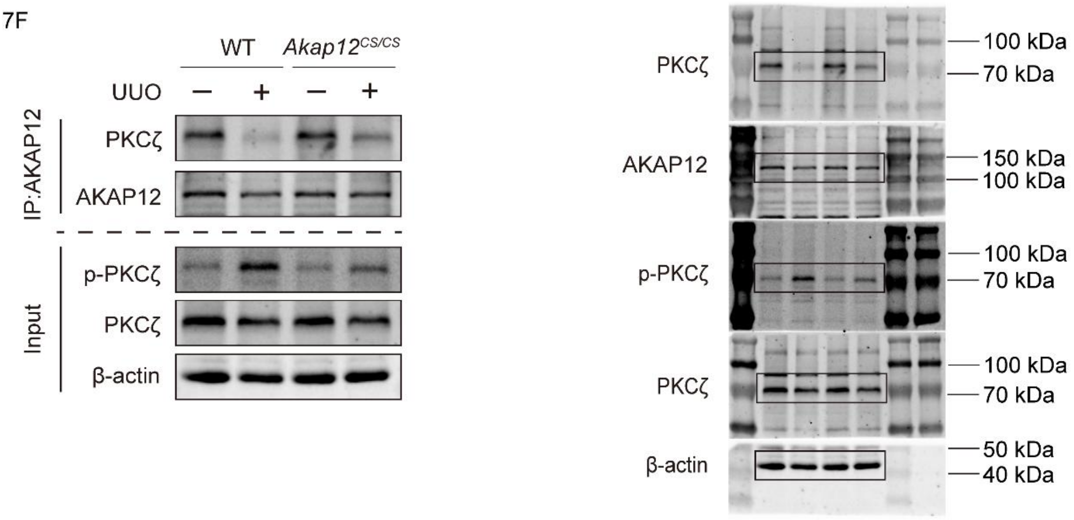

Figure 7G

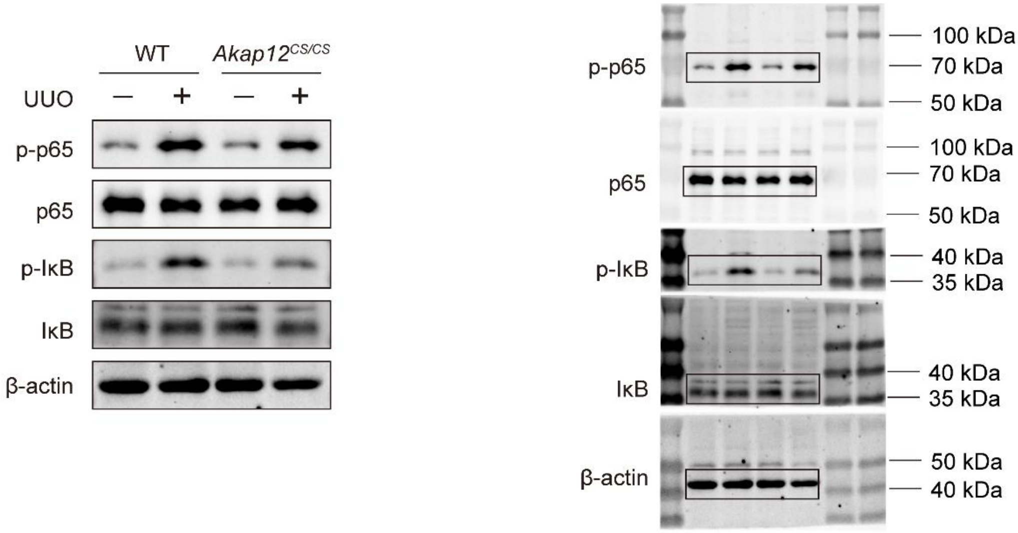

Figure 7H

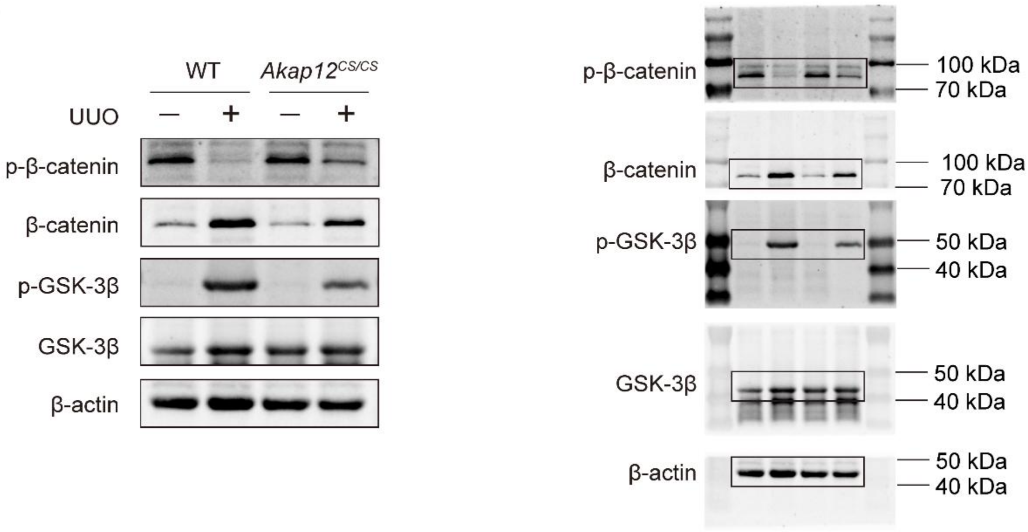

Figure 8F

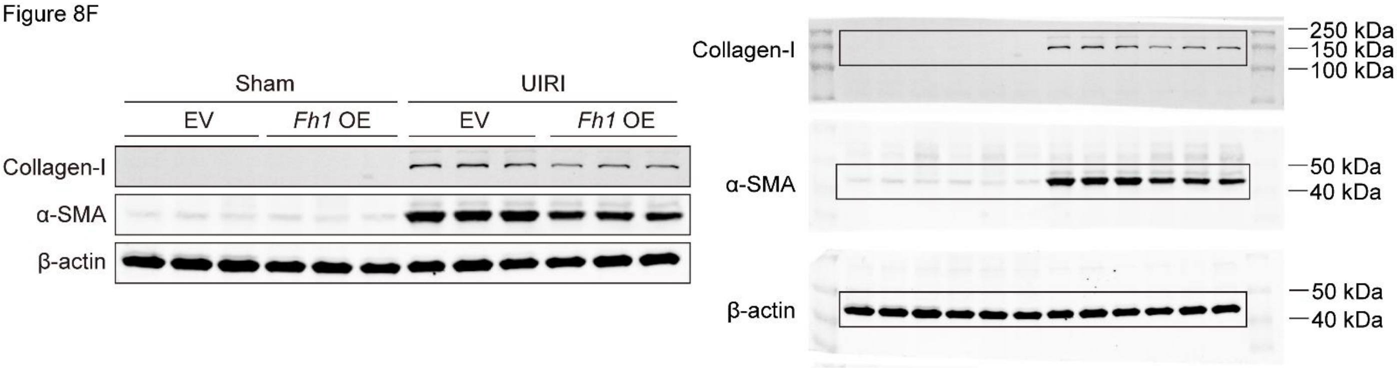

Supplemental Figure 2H

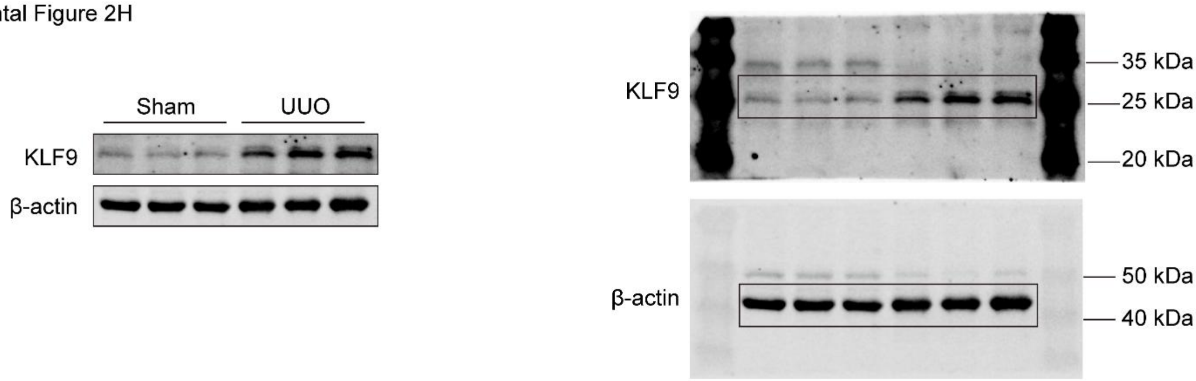

Supplemental Figure 2K

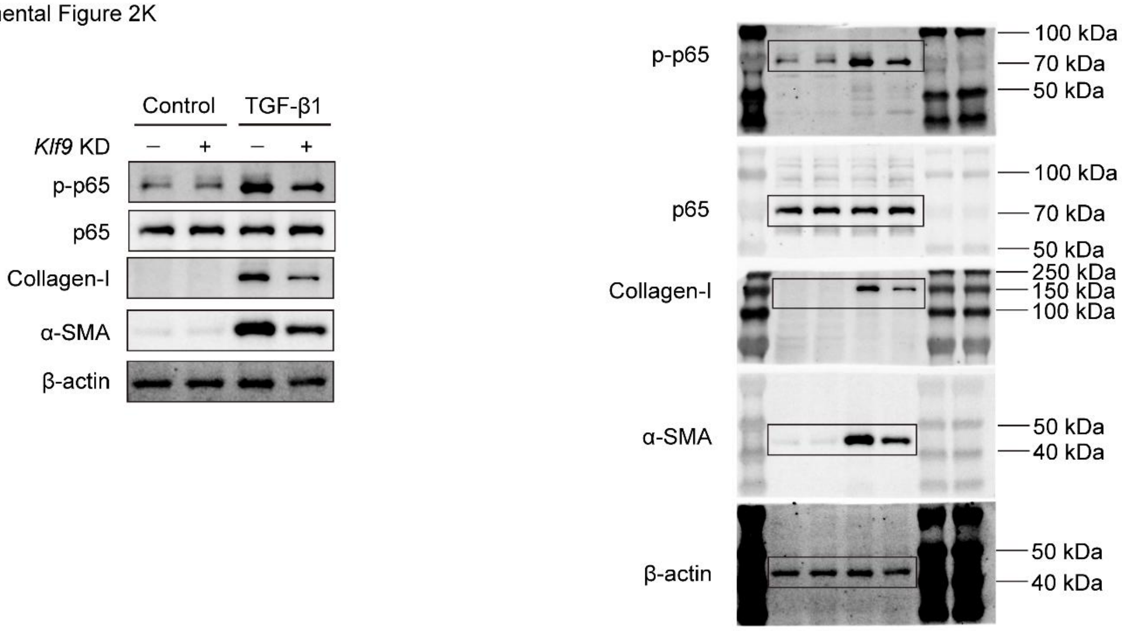

Supplemental Figure 3H

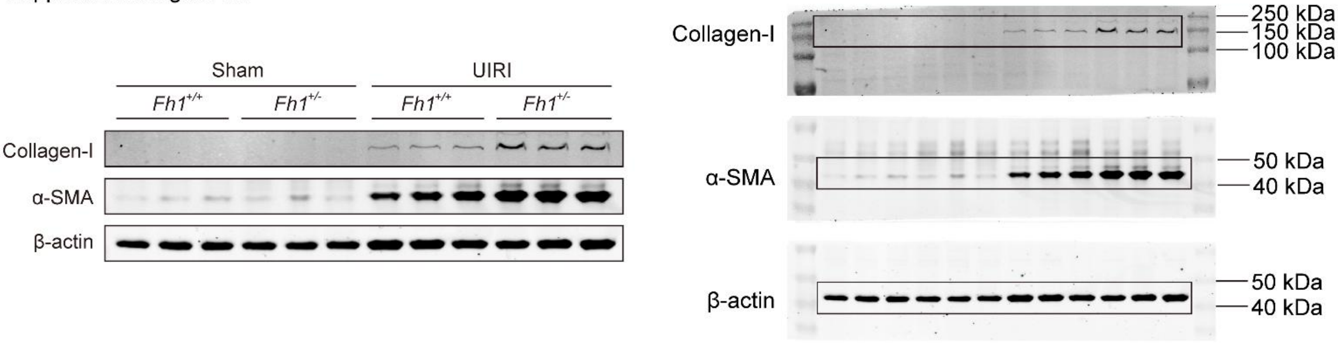

Supplemental Figure 5C

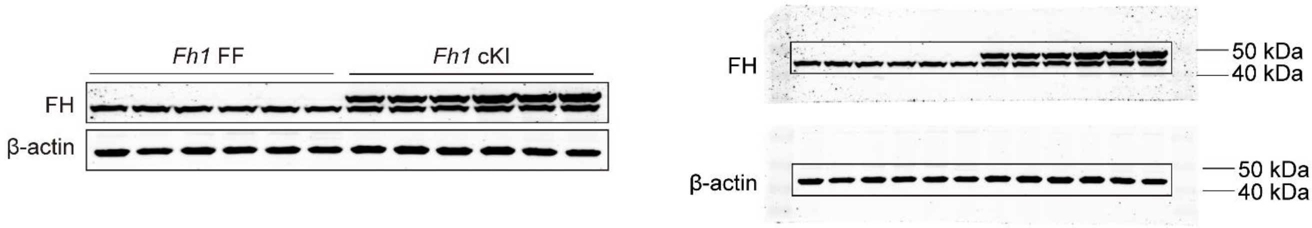

Supplemental Figure 5D

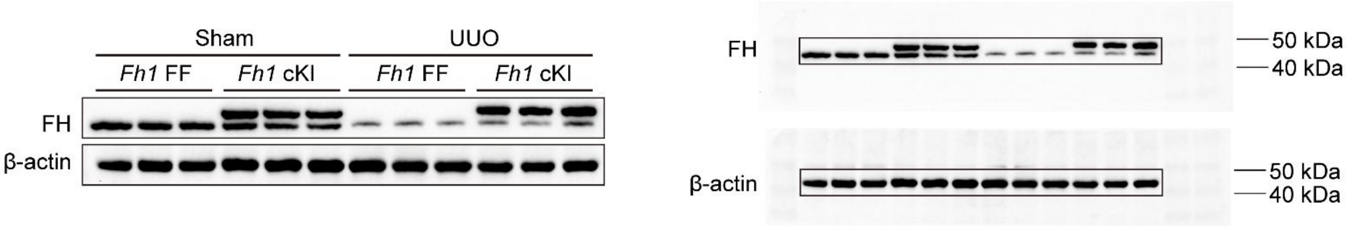

Supplemental Figure 7B

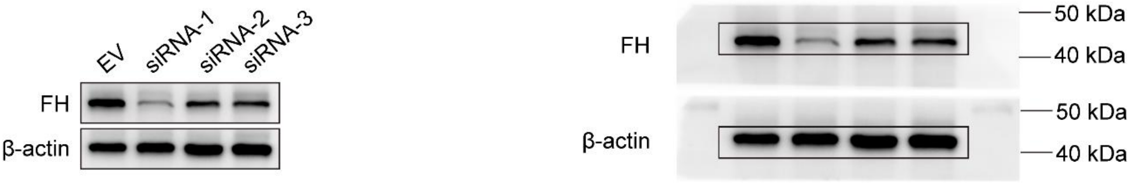

Supplemental Figure 7E

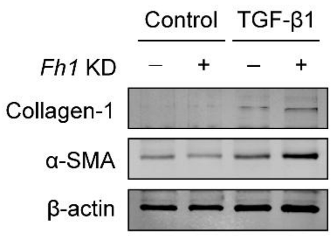

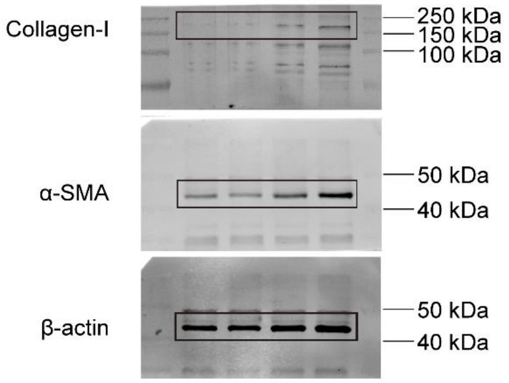

Supplemental Figure 7G

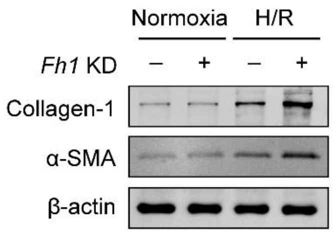

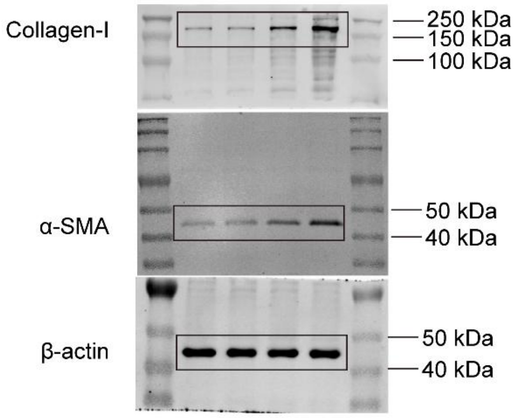

Supplemental Figure 8B

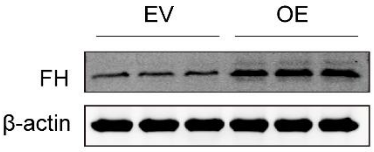

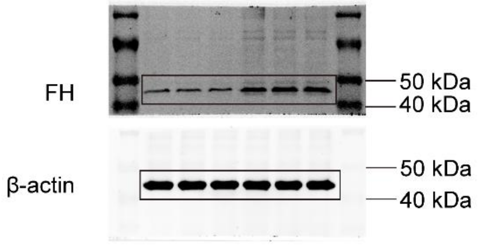

Supplemental Figure 8E

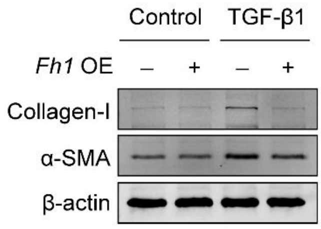

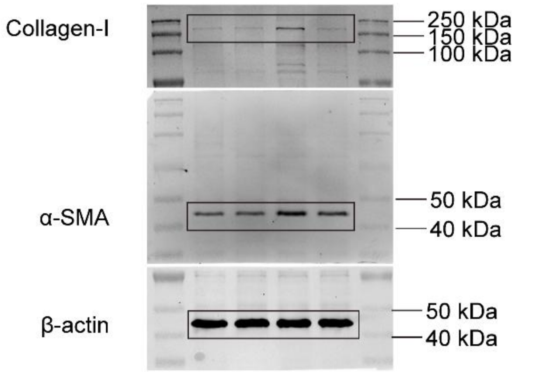

Supplemental Figure 8G

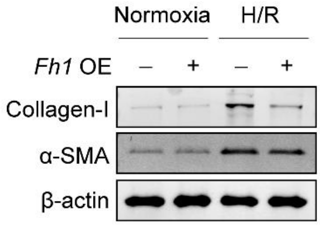

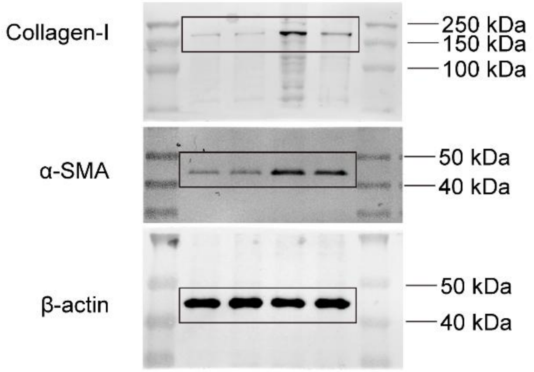

Supplemental Figure 9B

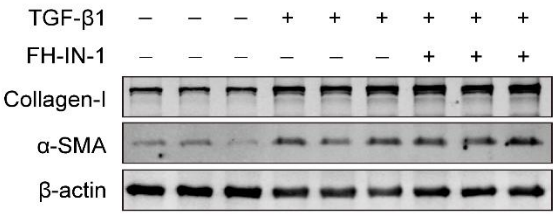

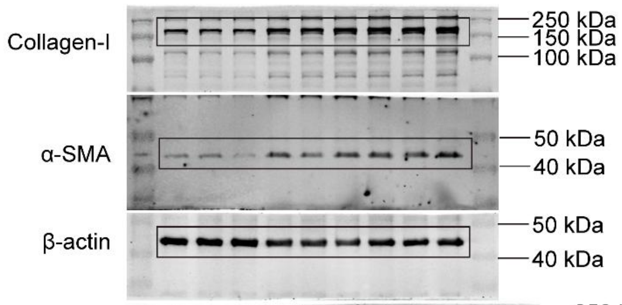

Supplemental Figure 12J

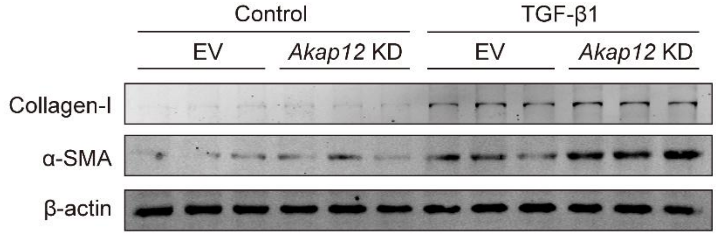

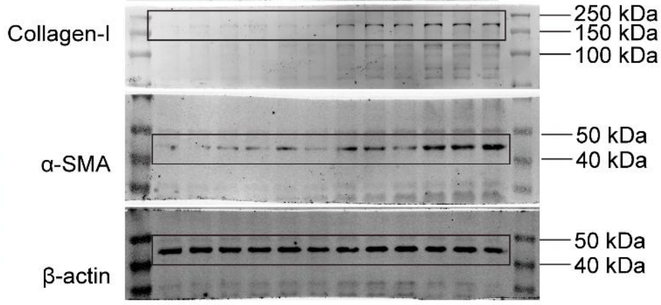

Supplemental Figure 13A

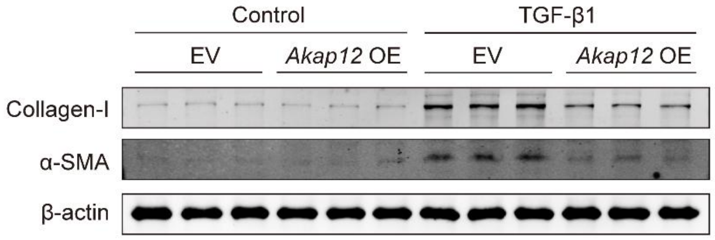

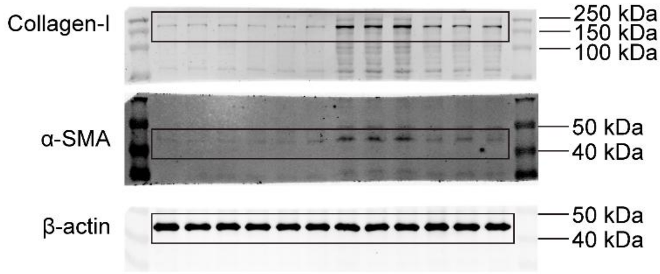

Supplemental Figure 13C

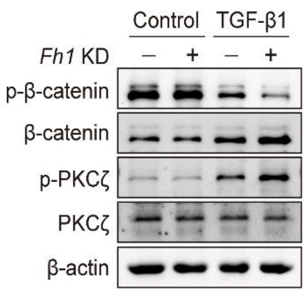

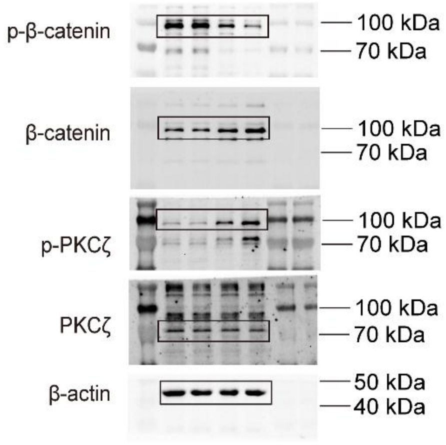

Supplemental Figure 13D

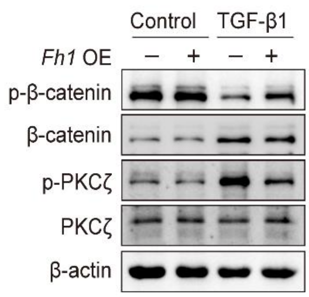

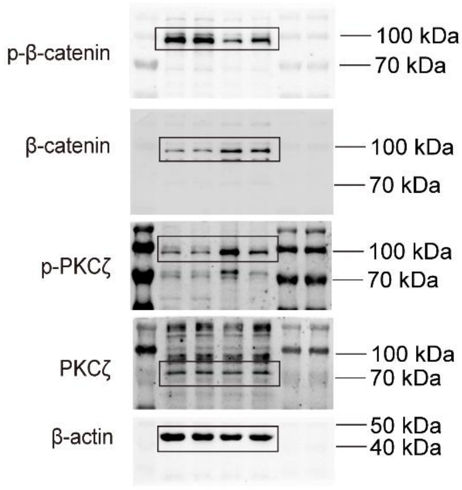

Supplemental Figure 13E

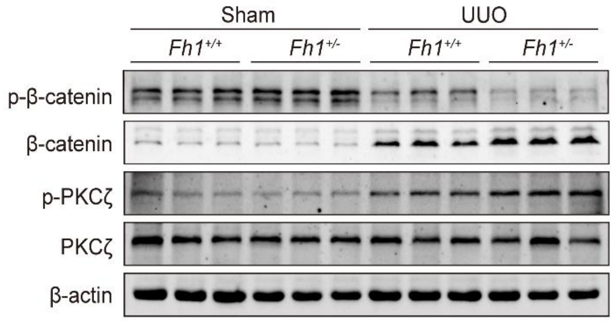

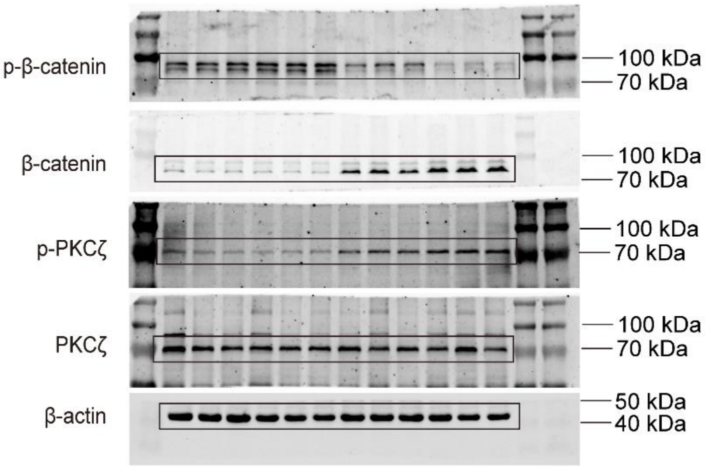

Supplemental Figure 13F

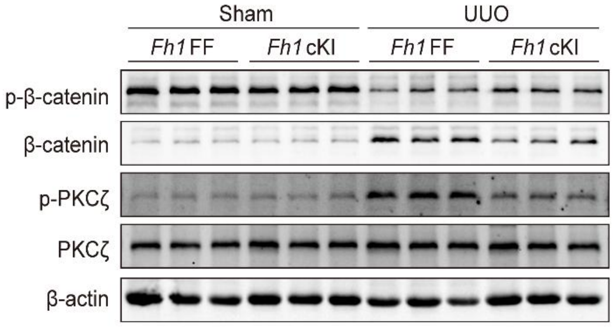

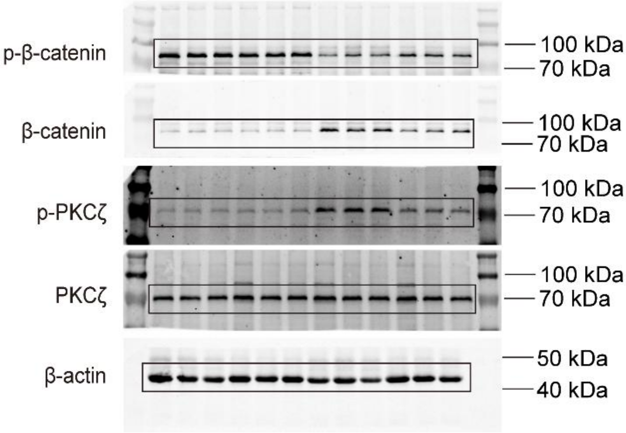

Supplemental Figure 15D

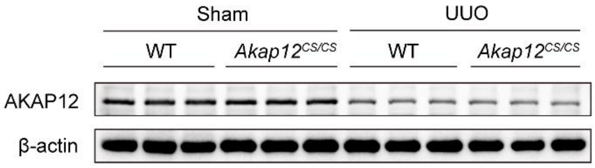

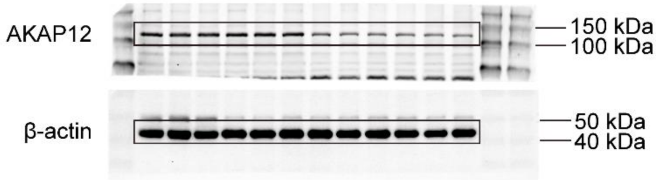

Supplemental Figure 15H

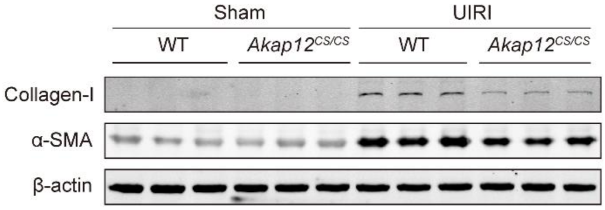

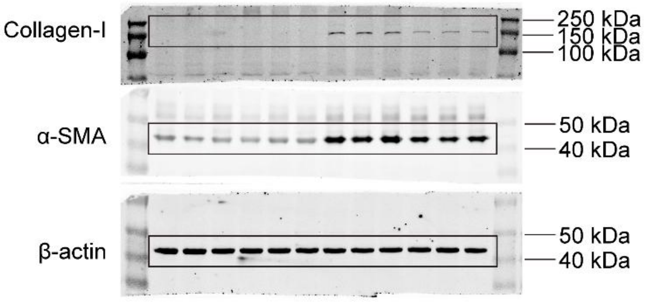

Supplemental Figure 18F

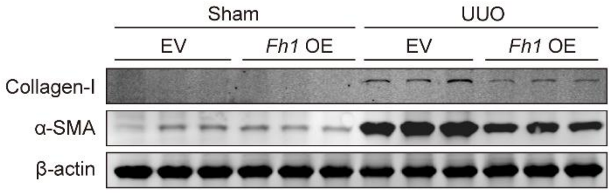

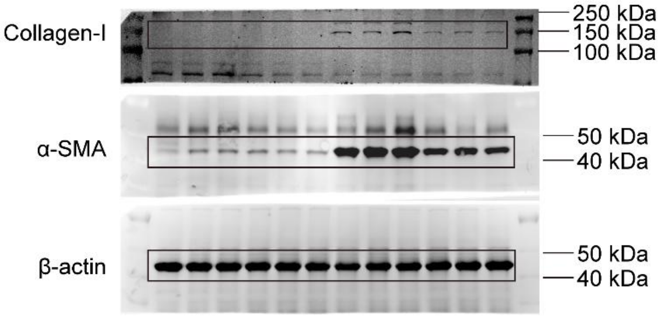

Supplement: Unedited blot and gel images [file jci-136-200755-s075.pdf]
